# Real-time pH imaging of macrophage lysosomes using the pH-sensitive probe ApHID

**DOI:** 10.1016/j.crmeth.2025.101203

**Published:** 2025-10-14

**Authors:** Santiago Solé-Domènech, Pradeep Kumar Singh, Lucy Funes, Cheng-I J. Ma, J. David Warren, Frederick R. Maxfield

**Affiliations:** 1 Department of Biochemistry and Biophysics, Weill Cornell Medicine, New York, NY 10065, USA

**Keywords:** endolysosomal pH, ratiometric imaging, pH sensors, ApHID, dextrans, fluorescence probes

## Abstract

Active endolysosomal pH regulation is essential for optimal enzymatic activity. To measure acidification, pH sensors can be delivered to acidic compartments using labeled dextran polymers or proteins. However, commercial probes have limited sensitivity in the acidic endolysosomal range or their fluorescence undergoes degradation. Herein, we introduce the new pH-sensitive probe *ApHID*, a green-emitting sensor with optimal dynamic range matching the acidity of endosomes and lysosomes. Acid pH indicator dye (ApHID) has a pKa near 5, increasing brightness with acidity, and withstands oxidation and photobleaching. We used ApHID dextrans to measure endolysosomal pH in macrophages and compared it to other commercially available sensors. ApHID reported pH accurately and stably over time in cell culture and was sensitive to subtle variations in organelle acidification in real time. Overall, ApHID circumvents limitations of currently available commercial probes and can provide utility in demanding applications such as intravital imaging of tissues.

## Highlights

• We developed ApHID, a sensor optimized for endolysosomal pH imaging
• ApHID outperforms commercial sensors in sensitivity and photostability
• ApHID detects subtle variations in endosomal pH within cells and acidic compartments
• ApHID-dextran fluorescence stability allows for a simplified pH calibration protocol

## Motivation

Commercially available fluorescent pH sensors have limited capacity to sense late endosomal and lysosomal (LE/Ly) pH. For prolonged fluorescence excitation or intravital imaging of tissues, which are subjected to extensive photobleaching and light scattering, bright probes with robust fluorescence, resistance to chemical modification, and excellent fluorescence dynamic range matching the acidity of acidic endosomes are optimal. With that in mind, we designed a BODIPY-based pH-sensitive probe, called ApHID (acid pH indicator dye), a green-emitting sensor with a pKa of ∼5 that brightens with increasing acidity. The probe can be attached to dextran polymers together with a pH-independent dye for ratiometric imaging, exhibits optimal fluorescence between pH 4.0 and 6.0, and shows robust resistance to chemical and enzymatic degradation and photobleaching.

## Graphical abstract

**Figure:**
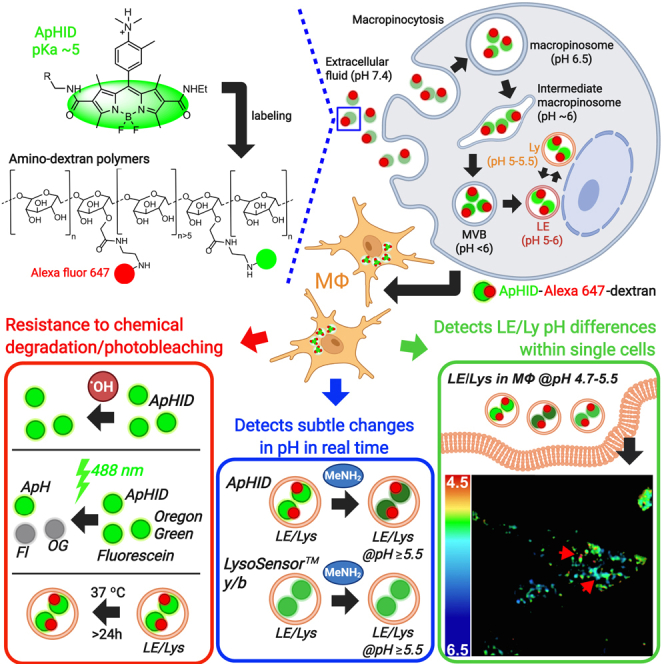

## Teaser

Solé-Domènech et al. present acid pH indicator dye (ApHID)*,* a green-emitting pH sensor with optimal fluorescence dynamic range matching the acidity of endolysosomal compartments. ApHID reports pH stably and detects subtle variations in endolysosomal acidification in real time. ApHID outperforms most commercial sensors and can be useful in demanding applications including intravital tissue imaging.

## Introduction

Mammalian cells use a variety of endocytic mechanisms to internalize small molecules, macromolecules, and particles that are delivered to specific sealed organelles.^1^ Late endosomes and lysosomes (LE/Lys) are membrane-bound vesicles containing more than 60 different hydrolases and more than 100 membrane proteins, which constitute the degradative organelles of the endocytic system.^2^ These organelles have the capacity to tightly regulate their intraluminal pH, which is required for maintaining optimal enzymatic activity.^3^^,^^4^^,^^5^ In most cells, during the 30–60 min following internalization, ligands encounter an increasingly acidic environment ranging from about pH 6 in sorting endosomes to a pH of 4.5–5 in lysosomes.^6^ A main regulator of vesicular pH is the V-ATPase complex, and with other ion transporters, it regulates membrane potential and vesicular pH, as well as organelle function.^7^

Preserving endolysosomal function is indispensable for cellular homeostasis.^8^ Lysosomal dysfunction plays a number of roles in many diseases, including Alzheimer disease (AD) and atherosclerosis, and lysosomal enzymatic deficiencies lead to lysosomal storage disorders such as Tay-Sachs disease and ceroid lipofuscinosis.^9^ LE/Ly membrane permeabilization can be caused by a variety of factors,^10^ and AD’s fibrillar amyloid-beta (Aβ) has been reported to damage LE/Lys and cause enzyme leakage.^11^ During AD pathogenesis, a deficiency in endolysosomal acidification has been shown to block autophagic flux, causing neurons to fill with undigested autophagic cargo, leading to extensive cellular damage.^12^ Also, it has been hypothesized that aging diminishes overall endolysosomal function, which could exacerbate neurodegenerative conditions.^13^ Microglia, the immune cells of the brain, can become activated in parts of the AD brain, causing damage to neurons, while probably inefficiently digesting fibrillar Aβ due to insufficient LE/Ly function.^14^^,^^15^ It is, therefore, important to find tools to visualize LE/Ly compartments and measure their acidification both in cell culture and *in vivo*. New methodology in this direction would aid in the understanding of pathogenic processes, for which our knowledge is still limited.

LE/Ly pH can be quantified in cells by fluorescence microscopy imaging of the compartments labeled with pH-sensitive probes, which can be delivered to the organelles using labeled dextrans or proteins.^1^ Once incorporated into the endocytic system, dextrans or proteins labeled with pH-sensitive probes reach endocytic compartments, where they serve as pH sensors. Organelle pH can be determined with precision by ratiometric imaging, and ratios measured can be interpolated to pH values using a ratio-to-pH calibration prepared using fixed cells. Sorting endosomes have a luminal pH of 5.9–6.0, whereas LE/Lys have a pH of 4.5–5.5.^1^^,^^16^ Hence, to accurately measure pH in these organelles, a probe must be most sensitive to the pH range of 4.0–6.0. We and others have used ratiometric pH imaging of dextrans or proteins labeled with various fluorescence probes to measure endosomal and lysosomal pH in many types of cells.^17^^,^^18^^,^^19^^,^^20^^,^^21^^,^^22^^,^^23^^,^^24^^,^^25^^,^^26^^,^^27^ Moreover, using ratiometric pH imaging we also measured the acidity of extracellular degradative compartments—*lysosomal synapses*—formed by macrophages and microglia toward large extracellular aggregates of low-density lipoprotein or Aβ. This mechanism, termed *digestive exophagy*, constitutes a pathway used by phagocytes to degrade, extracellularly, objects that are too large to be phagocytosed using secreted lysosomal enzymes.^28^^,^^29^

pH imaging can be achieved with a number of commercially-available pH sensors. A most popular sensor, fluorescein, has been used for decades to measure endosomal pH. However, fluorescein has a pKa of 6.5 and its brightness decreases with acidity, which limits its sensitivity in the acidic range of LE/Lys (pH 4.5–5.5). Fluorescein also undergoes rapid photobleaching, which curtails its uses in applications requiring extended imaging. In the past two decades, several pH-sensitive probes were developed commercially, including Oregon Green, pHrodo Green and Red series, and LysoSensor Yellow/Blue (LSyb), among others, with improved fluorescence dynamic range in the acidic spectrum of endosomes (Table S1). Oregon Green has a pKa of 4.7, and its dynamic range between pH 4 and 6 is better than that of fluorescein. However, its brightness also decreases with acidity, limiting its sensitivity in acidic pH. pHrodo Red and pHrodo Green increase brightness with acidity, but their pKa of 6.5, like fluorescein, limits their sensitivity in the pH range of LE/Lys. The pHrodo Deep Red probe, which was developed recently, has a pKa of 5.5 (attached to 70 kDa dextrans) and a good dynamic range between the pH 4.0–6.0 window, but it emits in the far-red spectrum and its brightness is modest, which limits its uses. The pHlys series of probes accumulate rapidly in acidic compartments, and although their structure is not disclosed, this could indicate a weak base nature. Also, ratiometric pH imaging with these sensors requires incubation with pH-sensitive and pH-independent dyes in separate steps, and the probes do not fully colocalize in the same acidic endosomes. Finally, Protonex Green, Magic, BioTracker Orange, and CypHer5E display increasing brightness with acidity, but once again their pKa (>6.5) limits their sensitivity in the acidic range of LE/Lys.

To circumvent these limitations, we designed a new water-soluble pH-sensitive probe, which we have called acid pH indicator dye (ApHID)*.* With a pKa near 5 and an emission maximum at 515 nm, ApHID displays an excellent fluorescence dynamic range within the pH of acidic organelles, and its brightness increases with acidity. ApHID fluorescence is highly resistant to photobleaching, and once incorporated into LE/Ly compartments, its fluorescence is stable and resistant to enzymatic degradation. We used dextrans labeled with ApHID and the pH-independent dye Alexa 647 to measure LE/Ly pH ratiometrically in macrophages, which allowed for identification of subtle pH differences between compartments. We also measured LE/Ly pH comparatively in macrophages using ApHID, fluorescein, or Oregon Green dextrans and obtained virtually identical pH readouts for all probes. Finally, we measured pH under alkalinizing conditions using ApHID and LSyb in parallel. LysoSensor showed limited sensitivity above pH 5, whereas ApHID reported LE/Ly acidity above 5.5 in a reliable manner. Overall, we believe that ApHID constitutes a promising tool that can prove useful in demanding imaging applications such as intravital imaging of tissues.

## Results

### Design of a pH-sensitive probe to measure acidity in endosomes and lysosomes

**Figure 1:**
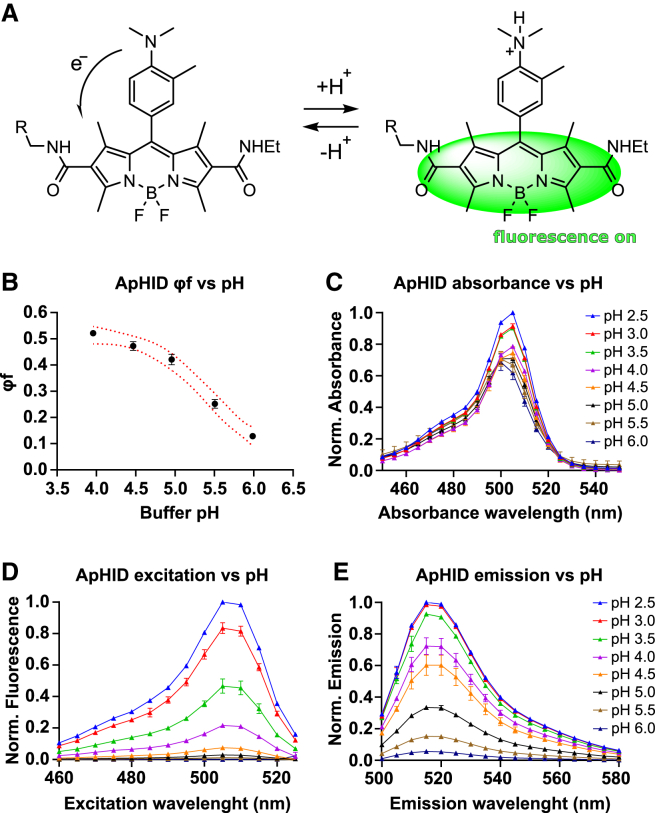
On-off switch and spectroscopic properties of ApHID (A) ApHID structure and on-off fluorescence switch. ApHID contains an aniline moiety acting as an electron donor at alkaline pH, which quenches fluorescence. The group “R” consists of a 10-carbon polyethylene glycol chain (PEG4), which ends in a succinimidyl ester group that confers increased solubility in water and allows for derivatization of molecules containing primary amines such as proteins or amino-dextran polymers. (B) Quantum yield (φf) of the hydrolyzed NHS ester form of ApHID measured in various pH-adjusted buffers, using fluorescein in 0.01 M NaOH (pH 12) as a standard. φf was plotted against pH, yielding a titration that was fit to a 4-component sigmoid with log IC_50_ (pKa) of 5.4. The maximal extinction coefficient and quantum yield of ApHID, measured in pH 3.0 buffer, are 99,710 M^−1^ cm^−1^ and 0.64, respectively. See Table S5 for statistics. (C–E) Absorbance (C), excitation (D), and emission (E) spectra plotted against buffer pH, measured for a 0.04 mg/mL dilution of 10-kDa amino-dextran labeled with ApHID at a 1.6:1 molar ratio, in pH-adjusted buffers. Two independent measurements were completed for each experiment. Geometrical objects and bars indicate averages ±SEM.

ApHID is composed of a BODIPY^30^ core with two flanking amide substitutions and an electrophilic nature (Figure 1A). The BODIPY core is attached to an aniline moiety with electron donor propensity that determines the range of pH sensitivity of the probe, and it can be conveniently modulated by attaching different alkyl groups to the nitrogen of the *N,N*-dialkyl-*o*-toluidine moiety. Based on a previous study by Maeda and collaborators,^31^ we attached two methyl groups to the nitrogen of *N*,*N*-dialkyl-*o*-toluidine moieties (Figure 1A). To provide water solubility to the otherwise insoluble structure, we attached a 10-carbon polyethylene glycol chain (PEG4) to one of the amide groups. The distal end of the PEG4 chain was derivatized with an *N*-hydroxysuccinimidyl ester (NHS) group, allowing for the labeling of proteins and other molecules such as amino-dextrans via reaction with primary amines (group R in Figure 1A).

The quantum yield (QY) for ApHID is 0.64 (measured relative to 5/6-carboxyfluorescein carboxylic acid [fluorescein] as a standard in 0.1 M NaOH), and its extinction coefficient is 99,700 M^−1^ cm^−1^, both measured in pH 3.0 citrate buffer (Table S5). The pKa value of ApHID (hydrolyzed NHS ester form) in solution is 5.4, based on its pH-dependent QY profile (Figure 1B). The UV-visible absorption spectra of ApHID remain similar between pH 4.0 and 6.0 (Figure 1C), but fluorescence emission spectra increase sharply in amplitude with increasing acidity (Figure 1D). Excitation spectra were also pH dependent, with an excitation maximum at 506 nm (Figure 1E).

### ApHID fluorescence and pKa remain stable in the presence of ⋅OH radicals, protein, or salts in solution

We compared the spectroscopic properties of ApHID conjugated to dextrans with those of commercially available pH-sensitive probes, namely, fluorescein, Oregon Green, and the popular probes LSyb, BioTracker Orange, and pHrodo Deep Red. Since LSyb is commercially available attached to 10 kDa dextrans , and since we will be using derivatized dextrans in subsequent experiments, we carried out our measurements using probes attached to polymers. Dextran labeling can be achieved by reacting the NHS ester form of the fluorophores with polymers previously derivatized with primary amines (amino-dextrans, see Table S3).

**Figure 2:**
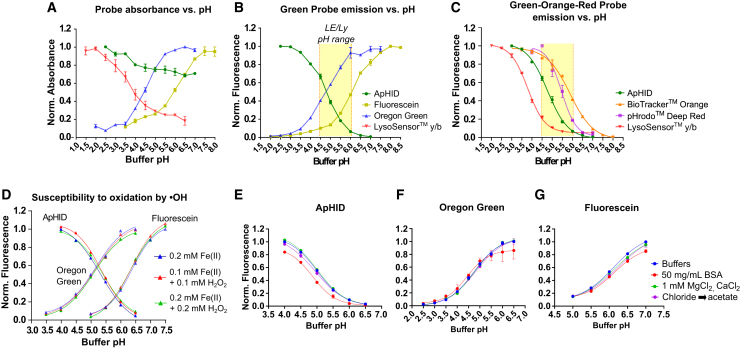
ApHID fluorescence and pKa are not affected by oxidation caused by ⋅OH radicals, protein, or various salt concentrations in solution (A–C) Absorbance (A) and fluorescence emission (B) vs. buffer pH of ApHID and other green- (B) and orange and red-emitting (C) probes measured in solution. Lysosensor yellow/blue and the rest of the probes were attached to 10 kDa (A, B) or 70 kDa (C) amino-dextran polymers and diluted to 0.02 mg/mL in various pH-adjusted buffers. ApHID absorbance decreases monotonically with increasing pH (A), and its emission increases strongly with increasing acidity (B, C). The yellow fields indicate the acidity range of LE/Lys (B, C). See Tables S6–S9 for normalized absorbance and fluorescence vs. buffer pH data, resulting pKa values, and dynamic ranges for each probe. (D) ApHID, Oregon Green, and fluorescein (hydrolyzed NHS ester forms) fluorescence titrations against buffer pH in the presence of hydroxyl radical (⋅OH) generated by mixing ferrous perchlorate (II) and H_2_O_2_ in solution, incubated at 37°C for 20 h. (E–G) ApHID (E), Oregon Green (F), and fluorescein (G) dyes attached to 10 kDa amino-dextrans were diluted to 0.04 mg/mL in pH-adjusted buffers enriched with 50 mg/mL BSA, 1 mM CaCl_2_, and 1 mM MgCl_2_, or sodium acetate (replacing sodium chloride in the buffer), and incubated at 37°C for 20 h. See Tables S10 and S11 for normalized fluorescence vs. buffer pH data and resulting pKa values for all probes and treatments. For all experiments, measurements were repeated twice. Normalized fluorescence intensities were plotted against buffer pH, resulting in various titrations and fit to 4-component sigmoidal curves. In all panels, geometrical objects and bars indicate average ±SEM.

Fluorophores attached to 10 or 70 kDa dextrans were solubilized in buffers with pH adjusted between 1.5 and 8.5 (see STAR Methods), and fluorescence and absorbance were measured and plotted against buffer pH. The resulting absorbance profiles for Oregon Green, fluorescein, and LSyb could be fit to a sigmoidal curve, but ApHID absorbance increased monotonically with increasing acidity (Figure 2A; Table S6). The fluorescence emission profiles for all probes fit a sigmoidal curve (Figures 2B and 2C). ApHID fluorescence at pH 4.0 is almost 13 times greater relative to pH 6.0. Fluorescein and Oregon Green fluorescence increases with alkalinity, being 7 and 4 times brighter at pH 6.0 relative to pH 4.0, respectively (Figure 2B; Tables S7 and S9). LSyb, a commonly used probe to measure LE/Ly pH, is 7 times brighter at pH 4.0 relative to pH 6.0, but its dynamic range within the pH 5.0–6.0 window is very limited (Figure 2C). The orange-emitting probe BioTracker Orange is only 2 times brighter at pH 4.0 relative to pH 6.0 (Figure 2C), whereas pHrodo Deep Red is 5 times brighter and shows good dynamic range between pH 4.0 and 6.0. However, its fluorescence has to be detected in the far-red spectrum, which somewhat limits its versatility (Figure 2C; Tables S8 and S9). By this analysis, ApHID has an excellent dynamic range between pH 4.0 and 6.0 and its fluorescence increases with acidity and can be detected in the green spectrum. This combination is a unique feature when compared with all other probes.

Dextrans derivatized with fluorophores can be used to label and track LE/Lys in cell culture. Depending on the cell type, the probes may be subjected to reactive oxygen species (ROS) generated intracellularly,^32^ which could cause chemical modifications that affect spectroscopic properties. The most reactive ROS species is the hydroxyl radical (⋅OH).^33^ To test the effects of ⋅OH on ApHID, fluorescein, and Oregon Green fluorescence, hydrolyzed NHS ester forms of the probes were added to PBS solutions containing 100 or 200 μM ⋅OH and incubated for 24 h at 37°C. The ⋅OH radicals were generated by the Fenton reaction by mixing ferrous perchlorate, Fe(II), with H_2_O_2_ in solution. As a control, we incubated the probes with ferrous perchlorate in the absence of H_2_O_2._ The intracellular concentration of H_2_O_2_ in various cell types, under physiological conditions, is within the range of 1–10 μM.^34^^,^^35^ However, ROS concentrations in some cancer microenvironments can be as high as 100 μM, depending on the activation status and metabolic state of the cells.^36^ We conducted our assay with conditions that would ensure ⋅OH concentrations above these reported levels. Following reaction, the mixtures were diluted in buffers with pH ranging between 4 and 7.5, and fluorescence was measured and plotted against buffer pH (Figure 2D). Following ROS exposure, only small variations in pKa were observed, indicating that the probes were resistant to oxidation by ⋅OH for at least 24 h (Tables S2 and S10).

Fluorophores reaching acidic compartments might also be sensitive to the high protein^2^ and salt^37^^,^^38^ concentration present in the organelles. To test for this, 10 kDa dextrans labeled with the probes were incubated in buffers containing 50 mg/mL BSA, 1 mM MgCl_2_ and CaCl_2_, or sodium acetate in the absence of sodium chloride for 24 h at 37°C and fluorescence was quantified and plotted against buffer pH thereafter. The probes were generally stable in the presence of salts, but their fluorescence decreased in the presence of BSA (Figures 2E–2G). The pKa of the probes did not register large variations over the various conditions tested (Tables S2 and S11), but it was generally slightly lower when attached to dextrans, relative to its unattached molecular form. This effect has been previously reported for fluorescein.^39^

### ApHID is resistant to photobleaching

**Figure 3:**
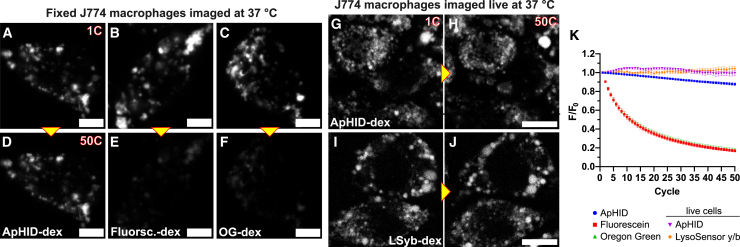
ApHID is highly resistant to laser-induced photobleaching (A–F) Comparative photostability of ApHID (A and D), fluorescein (B and E), and Oregon Green (C and F) in LE/Lys of fixed J774 macrophages, imaged by confocal microscopy. Cells were incubated with 70 kDa amino-dextrans labeled with the various NHS ester probes at 1 mg/mL in DMEM medium overnight, followed by a 4-h chase and fixation in PFA. Fixed cells were imaged in 50 mM TRIS maleate pH 5.0 buffer (ApHID and Oregon Green) or in 1X PBS pH 7.4 buffer (fluorescein) containing membrane-permeant equilibrators. Single-cell planes were irradiated for 50 cycles (0.5 s per pulse) using a 488-nm argon gas laser adjusted to an output of 5 μW with 1-s intervals between cycles. (G–J) Comparative photostability of ApHID (G and H) and LysoSensor yellow/blue (LSyb, I and J) in live J774 macrophages. Cells were incubated with 70 kDa amino-dextrans labeled with NHS-ApHID as described above or with 10 kDa LSyb-dextran at 1 mg/mL in cell medium overnight, followed by a chase, and allowed to equilibrate in a confocal microscope incubation chamber at 37°C in 5% CO_2_ for 1 h prior to imaging. ApHID was excited using a solid-state white laser adjusted to 488 nm, whereas LSyb was excited with a 405-nm solid-state laser, both adjusted to an output of 5 μW. Single planes were irradiated for 50 cycles (0.6 s per cycle) with 8-s intervals between cycles. (K) Fluorescence intensity per dish or well for each probe and cycle (F) was normalized to its initial fluorescence (F_0_) and plotted against irradiation cycle (F/F_0_). The experiments were repeated twice; 2 dishes or wells were measured per condition and experiment, and 3–4 fields were imaged per dish or well. See Table S12 for statistics. For all measurements, geometrical shapes indicate average F/F_0_ for each irradiation cycle ±SEM (most error bars fit within the symbols). Laser output was measured at the front element of the objectives using an external power meter. Pixel dwell time was 33 μs for all experiments. Scale bar: 5 μm in (A–F) and 10 μm in (H–K).

Live cell and intravital fluorescence imaging conducted for extended periods of time benefit from probes that can withstand prolonged photoexcitation while conserving their fluorescence properties. We measured ApHID photostability and compared it to that of fluorescein and Oregon Green in fixed cells. J774 macrophages were incubated overnight with 70 kDa dextrans labeled with the various probes, followed by a 4-h chase to ensure localization in LE/Ly compartments. Cells were then fixed in PFA and incubated at 37°C in 50 mM TRIS maleate pH 5.0 buffer (for ApHID and Oregon Green) or 1X PBS pH 7.4 buffer (for fluorescein) containing the membrane-permeant equilibrators sodium acetate, methylamine, and the ionophores nigericin and monensin to ensure buffer equilibration across membranes. Once equilibrated, cells were imaged in a confocal microscope while being irradiated with a 488-nm argon laser for 0.5 s per cycle (50 cycles in total) with 1-s intervals between irradiation pulses, and images were acquired after each cycle (Figures 3A–3F). Additionally, ApHID photostability was also compared with that of LSyb (attached to 10 kDa dextran, commercially available from Thermo Fisher) in live J774 macrophages. Cells were incubated with dextrans as described above and chased for 4 h, followed by imaging at 37°C in 5% CO_2_ (Figures 3G–3J). For all experiments, laser output was adjusted to yield 5 μW power at the front element of the objectives using an external power meter. Fluorescence intensity for each probe and cycle (F) was normalized to fluorescence on cycle 1 (F_0_) and plotted as F/F_0_ against irradiation time (Figure 3K; Table S12). Following laser irradiation, ApHID fluorescence intensity in fixed cells had decreased by 12%, whereas that of fluorescein and Oregon Green had decreased by 83% and 82%, respectively. Interestingly, both ApHID and LSyb showed no detectable photobleaching when irradiated in live cells (Figure 3G; Table S12). These results indicate that ApHID is highly resistant to laser-induced photobleaching.

### ApHID fluorescence and pKa remain stable with different degrees of amino-dextran derivatization and net charge

**Figure 4:**
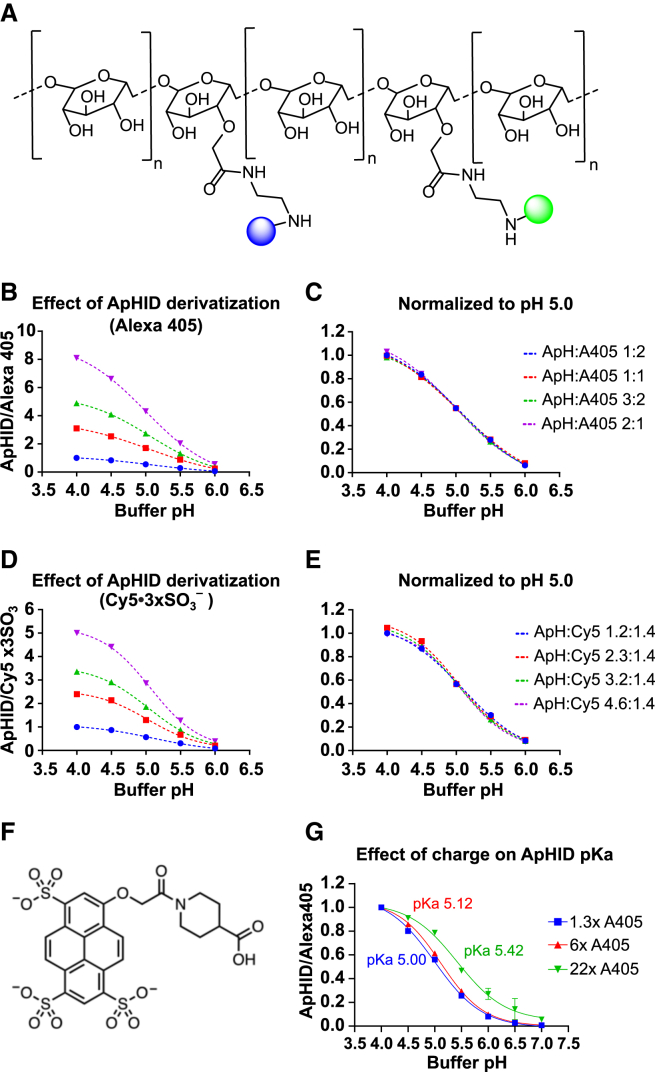
Effect of various amounts of pH-independent fluorophores and dextran charge density on ApHID fluorescence and pKa profiles (A) Representative structure of a dextran polymer containing lysine groups that can be derivatized with pH-sensitive (green spheres) and pH-independent (blue spheres) probes. (B–E) ApHID/Alexa 405 (B and C) and ApHID/Cy5⋅3SO_3_^−^ (D and E) ratios measured for amino-dextrans labeled with various amounts of ApHID, and a constant amount of either pH-independent dye, solubilized at 0.04 mg/mL in pH-adjusted buffers. Ratios were plotted against buffer pH. The titrations were either normalized to the ApHID/pH-independent ratio for the dextran labeled with the least amount of ApHID at pH 4.0 (B and D) or by the ApHID/pH-independent ratio for the dextran with the highest content in ApHID at pH 5.0 (C and E). (F and G) ApHID/Alexa 405 ratios were measured for dextrans labeled with various amounts of Alexa 405 dye and a constant amount of ApHID. Each Alexa 405 molecule carries three negatively charged sulfate groups (F). Fluorescence ratios were plotted against buffer pH, and the various titrations were fit to a 4-component sigmoid from which log IC_50_ (pKa) was calculated. pKa values are indicated next to each titration (G). See Table S13 for normalized fluorescence vs. pH data, ApHID pKa values, and statistics for all conditions. Experiments were repeated three times. Geometrical objects and bars indicate averages ±SEM (most error bars fit within the symbols). Abbreviations: A405, Alexa Fluor 405; Cy5, Cy5⋅3SO_3_^−^; ApH, ApHID.

Amino-dextrans can be derivatized with a variety of pH-sensitive and pH-independent dyes (Figure 4A, green and blue spheres, respectively). Increasing dextran derivatization should increase overall brightness, which would be beneficial for applications with taxing light scattering or when extended imaging times and reduced laser power are required. To test for the effect of dextran derivatization on ApHID fluorescence and pKa, we labeled 70 kDa polymers with different amounts of ApHID and a constant amount of Alexa 405 or Cy5⋅3xSO_3_^–^^40^ (pH-independent) and measured fluorescence against buffer pH in solution. Increasing derivatization with ApHID increases ApHID/Alexa 405 and ApHID/Cy5⋅3xSO_3_^–^ ratios proportionally to their molar ratios (Figures 4B and 4D). However, when normalized to pH 5.0 ratio, the resulting sigmoidal curves are not significantly different from each other (Figures 4C and 4E; Table S13). This indicates that ApHID fluorescence and pKa remain stable at a range of probe concentrations on the dextran. We also tested the effect of dextran net charge on ApHID pKa. To do that, we derivatized dextrans with a constant amount of ApHID and various amounts of Alexa 405, which carries three negatively charged sulfate groups (Figure 4F). Raising the dextran negative charge with Alexa 405 increased ApHID pKa, but only at high negative charge density (Figure 4G; Table S13). These results indicate that ApHID fluorescence and pKa remain stable in a wide range of dextran derivatizations.

### ApHID is not cytotoxic, withstands enzymatic degradation in endolysosomal compartments, and detects small differences in acidification between individual compartments within single cells

Endosomal acidification can be measured using pH-sensitive probes delivered to the target organelles. The use of dextran polymers to label LE/Ly compartments has been widely reported and addressed in the literature. In most cells, dextrans are endocytosed by fluid-phase pinocytosis, trafficked into sorting endosomes within 3–10 min and to LE/Lys within 20–30 min of initial internalization (extensively reviewed in Mukherjee et al.^1^). Additionally, in macrophages, the mannose receptor mediates the uptake of dextran via receptor-mediated endocytosis.^41^ There is a certain degree of dextran exchange between LE/Lys, but the polymers do not escape these organelles. After several hours of incubation with dextrans, more than 80% of vesicles loaded with polymers are positive for Rab7 and LAMP1, which are standard markers of LE/Ly compartments.^42^^,^^43^

**Figure 5:**
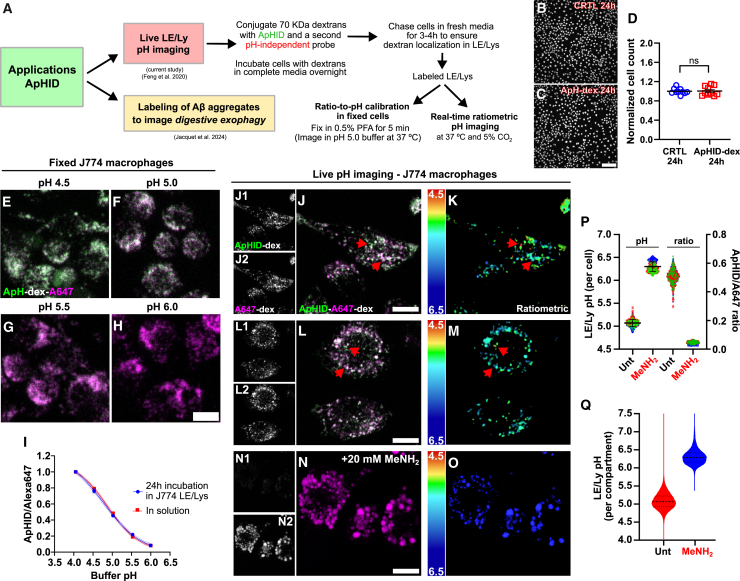
ApHID-dextran is not cytotoxic, withstands endolysosomal enzymatic degradation, and is sensitive to small differences in acidification between individual LE/Ly compartments within single cells (A) ApHID applications and summarized experimental approach to load endolysosomal compartments and measure their pH using amino-dextran polymers. (B–D) Cytotoxicity of ApHID-dextran on J774 macrophages. Cells were incubated with 0.5 mg/mL amino-dextrans (70 kDa) labeled with NHS-ApHID, or left untreated, overnight, followed by a 4-h chase in fresh DMEM medium and staining with Hoechst nuclear marker. Cells were imaged live by confocal imaging at 37°C in 5% CO_2_ (B, C). Cell nuclei were quantified by digital image analysis and normalized to the untreated condition (D). The experiment was repeated 3 times; 23 wells were imaged per experiment (10 wells in total), and 16 fields were acquired per well. The total number of cells per well was calculated by summing the nuclei counts from all imaged fields in each well. Geometric shapes and bars in (D) indicate normalized average cell counts ±SEM. Differences in cell count means between conditions were assessed using the two-tailed unpaired Student’s t test. *p* value shown as *p* > 0.05 (ns). See Table S14 for statistics. (E–I) Calibration confocal microscopy images of LE/Ly compartments in J774 macrophages, loaded with amino-dextrans labeled with NHS-ApHID and NHS-Alexa 647. Cells were incubated with 0.5 mg/mL dextrans overnight and chased for 3 h in fresh DMEM medium the following morning. The cells were then fixed in 0.5% PFA and incubated for 20 min in pH 4.0 and pH 4.5 buffer or 30 min in pH 4.5–6.0 buffers containing membrane-permeant equilibrators at 27°C, followed by confocal imaging (E–H). ApHID/Alexa 647 ratios were calculated for each field and plotted against buffer pH. The resulting titration was fit to a 4-component sigmoidal curve and compared with a titration of the same dextran measured in solution using a spectrophotometer, also at 27°C (I, blue and red lines). The experiment was repeated three times; 2 wells were imaged for each pH-adjusted buffer tested, and 3 fields were acquired per well. Geometrical objects and bars indicate averaged ratios ±SEM (most bars are within the geometrical objects). See Table S15 for statistics. (J–O) Live confocal ratiometric pH imaging of LE/Ly compartments in J774 macrophages. LE/Lys (examples highlighted by arrowheads) were loaded with amino-dextrans labeled with NHS-ApHID and NHS-Alexa 647 and imaged ratiometrically at 37°C in 5% CO_2_ (J, L, and N). Some cells were treated with 20 mM methylamine to alkalinize compartments (N–O). Side panels in (J, L, and N) show ApHID and Alexa 647 channels individually. Pixel-by-pixel pH values were calculated ratiometrically and mapped to a color scale, indicated by adjacent color-coded pH bars (K, M, and O). (P and Q) ApHID/Alexa 647 ratios were calculated and interpolated to pH values using a ratio-to-pH calibration prepared in fixed cells. Ratios and corresponding pH values were plotted per cell (P) or per compartment (violin plot, Q) for each condition. The experiment was repeated three times; 3 wells were imaged per condition, and 4 fields were acquired per well. A total of 603 and 683 cells were quantified for the untreated and methylamine-treated conditions, respectively. Geometric shapes and bars indicate average LE/Ly pH ± SEM. See Table S16 for statistics. Scale bars: 100 μm in (B and C) and 10 μm (elsewhere). MeNH_2_: methylamine.

Figure 5A shows a diagram with the described uses of ApHID and our experimental approach to measure LE/Ly pH using dextrans. In the current study, we used 70 kDa dextrans conjugated with various fluorescent probes to measure LE/Ly pH by confocal ratiometric imaging. We also applied a method that simplifies the preparation of the fluorescence ratio-to-pH calibration required to interpolate fluorescence ratios to pH, a typically cumbersome part of the protocol.

To measure pH in cell culture using ApHID, J774 macrophages were incubated with dextrans labeled with the probe and Alexa 647 (pH-independent) overnight, followed by a 3-h chase in fresh DMEM to ensure LE/Ly colocalization. ApHID-dextran is not cytotoxic, as overnight incubation with labeled polymers did not alter cell viability relative to unlabeled cells (Figures 5B–5D; Table S14). After dextran loading, chase, and fixation in PFA, cells incubated in pH 4.5 buffer showed strong ApHID fluorescence, which decreased with increasing buffer alkalinity (Figures 5E–5I). To quantify ApHID/Alexa 647 ratios in LE/Lys, an intensity threshold was applied to the pH-independent channel (Alexa 647). A mask was then generated and transferred to the pH-dependent channel. Integrated intensity was measured for each masked channel, and pH ratios were calculated for each LE/Ly, cell, or field imaged. ApHID/Alexa 647 ratios measured in fixed cells were plotted against buffer pH, and the resulting titration was fit to a 4-component sigmoidal curve, which matched that obtained for the same dextrans measured in solution, demonstrating ApHID and Alexa 647 resistance to endolysosomal enzymatic degradation (Figure 5I; Table S15).

Next, we measured LE/Ly pH in cell culture. ApHID and Alexa 647 fluorescence in LE/Ly compartments was imaged using confocal microscopy (Figures 5J–5O), and ApHID/Alexa 647 ratios for each cell or LE/Ly compartment were calculated and interpolated to pH (Figures 5P and 5Q) using a ratio-to-pH calibration curve. To prepare the curve, ApHID/Alexa 647 fluorescence ratio was measured in fixed cells incubated in pH 5.0 buffer, and the resulting ratio value was used to generate all subsequent ratios for pH points 3.5–7.4, using titration data previously obtained in solution using a spectrophotometer (as in Figures 2B and 2C; S3; Table S4). 50 mM TRIS maleate pH 5.0 buffer equilibrates well across fixed cell membranes at 37°C without causing significant membrane or cell swelling (Figure S1), but membrane permeation with sodium acetate, methylamine hydrochloride, and monensin^44^^,^^45^ is required for efficient buffer equilibration (Figure S2). This approach is appropriate because the pH-dependent dynamic range of ApHID (and most pH-sensitive probes) measured in cell culture is identical to that measured in solution (Figures 5E–5I). Also, the pH dependence of ApHID remains constant within a wide range of labeling of the dextran polymer (Figure 4). Therefore, dextran batches with different degrees of probe incorporation still show the same pH response. ApHID-reported LE/Ly acidity fell within the range of pH 4.7–5.5 (Figures 5P and 5Q; Table S16), in line with previous findings.^18^^,^^19^^,^^46^ Importantly, ApHID demonstrated small differences in pH between individual compartments within single cells (Figures 5J and 5L and color-coded ratio images in Figures 5K and 5M, arrowheads). A brief treatment with 20 mM methylamine induced rapid alkalinization (Figures 5N–5Q), indicating that, once in the LE/Lys, ApHID is sensitive to changes in pH in real time.

### LE/Ly pH measured in J774 macrophages using ApHID is stable over time and coincides with measurements done using fluorescein and Oregon Green

Next, we wanted to test whether ApHID reports LE/Ly pH as reliably as fluorescein, a well-characterized probe used to measure pH in multiple cell types.^18^^,^^47^ A second popular probe, Oregon Green,^19^^,^^48^ was also tested for comparative purposes. This time, we used a semi-high-throughput methodology; we imaged wells with seeded cells, labeled with the various probes, left untreated or treated with methylamine, using a 20× air objective and faster acquisition settings. This reduced the overall resolution of the images but significantly increased data acquisition and speed capabilities.

**Figure 6:**
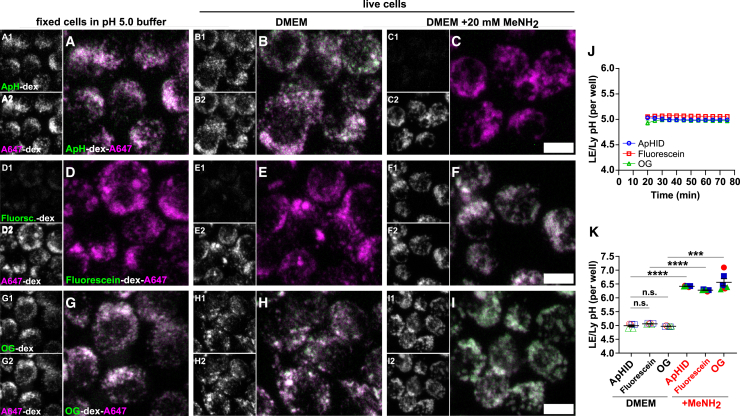
Endolysosomal pH reported by ApHID in J774 macrophages matches that reported by fluorescein and Oregon Green (A–I) Confocal micrographs of LE/Ly compartments loaded with dextrans in J774 macrophages. Cells were incubated with 0.5 mg/mL amino-dextrans overnight and chased for 3 h in fresh DMEM medium the following morning, followed by confocal imaging. The polymers were labeled with NHS-ApHID (A–C), NHS-fluorescein (D–F), or NHS-Oregon Green (G–I) and NHS-Alexa 647 (pH-independent). Some cells were fixed and imaged in 50 mM TRIS maleate pH 5.0 buffer containing membrane-permeant equilibrators, for each probe (A, D, and G). Cells imaged live at 37°C in 5% CO_2_ were either left untreated (B, E, and H) or briefly treated with 20 mM methylamine to alkalinize compartments (C, F, and I). Side panels in (A–I) show fluorescence channels for all probes individually. (J) Endolysosomal pH measured over time. Probe fluorescence was measured every 5 min for 75 min, and calculated fluorescence ratios were interpolated to pH and plotted against incubation time for each probe. To interpolate ratios to pH, we used a calibration prepared in fixed cells imaged in pH 5.0 buffer containing membrane-permeant equilibrators. (K) Endolysosomal pH reported by each probe and treatment after 1 h equilibration at 37°C in 5% CO_2_. Fluorescence ratios were interpolated to pH as described above. All experiments were repeated three times; 2 wells were imaged for each condition, and 4 fields were acquired per well. Geometrical objects and bars indicate averaged pH ± SEM (most error bars fit within the symbols). In (K), differences in LE/Ly pH means between conditions were assessed using the unpaired one-way ANOVA followed by Dunnett’s multiple comparison test with 95% confidence interval. *p* values shown as *p* > 0.05 (ns); *p* ≤ 0.001 (∗∗∗), *p* ≤ 0.0001 (∗∗∗∗). See Tables S17 and S18 for statistics on pH measurements. Scale bars: 10 μm. OG, Oregon Green; A647, Alexa Fluor 647; ApH, ApHID; MeNH_2_, methylamine.

J774 macrophages were loaded overnight with 70 kDa dextrans labeled with the pH sensors and Alexa 647, followed by a 3-h chase in fresh DMEM. As expected, the fluorescein signal at pH 5.0 (Figure 6D) or in living cells (Figure 6E) was substantially weaker relative to that of ApHID (Figures 6A and 6B), whereas that of Oregon Green (Figures 6G and 6H) was comparable. When 20 mM methylamine was added to the media, ApHID signal decreased dramatically (Figure 6C), but fluorescein and Oregon Green became substantially brighter (Figures 6F and 6I). This is consistent with the pH-dependent fluorescence properties reported for these probes (Figure 2B), which reflect the acidic nature of LE/Lys.

Next, we compared LE/Ly pH reported by each probe in living cells using confocal microscopy. Probe fluorescence was measured every 5 min over the course of 75 min. pH-sensitive/Alexa 647 ratios were calculated and interpolated to pH using a ratio-to-pH calibration curve for each probe as described earlier (Figure S3; Table S1). LE/Ly pH was stable over time independently of the reporting probe (Figure 6J; Table S17). The averaged LE/Ly pH reported by ApHID after 1 h equilibration at 37°C was within 0.1 pH units of that reported by fluorescein and Oregon Green, and differences in the average pH reported by the probes were not statistically significant. Methylamine treatment rapidly alkalinized LE/Ly pH to ∼6.3–6.5, which was detected by all sensors (Figure 6K; Table S18).

In summary, ApHID reports virtually identical LE/Ly pH values as fluorescein and Oregon Green, and the reported acidity was found to be stable over time when imaged in equilibrated, constant temperature conditions. This validates the use of ApHID for ratiometric pH imaging of acidic vesicles in cell culture.

### ApHID reports LE/Ly pH in a stable manner over time and is sensitive to subtle increases in alkalinization in real time

**Figure 7:**
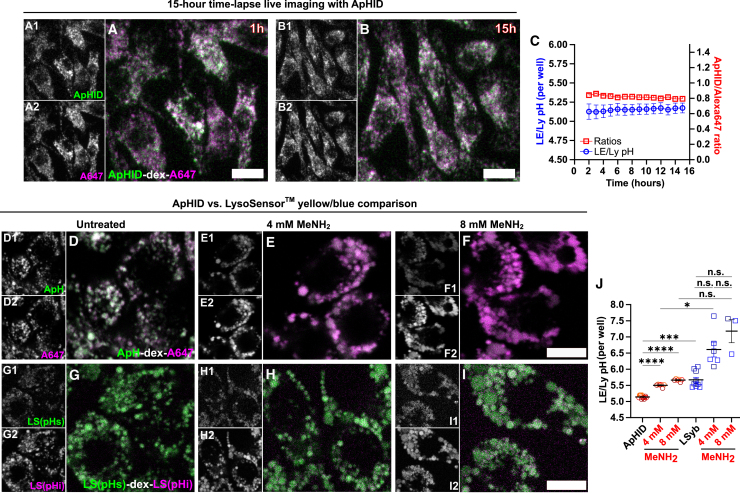
ApHID reports endolysosomal pH in stable manner over prolonged periods of time and is sensitive to subtle alkalinization in real time (A–C) LE/Ly compartments in primary mouse bone marrow-derived macrophages loaded with amino-dextrans tagged with NHS-ApHID and NHS-Alexa 647. Cells were equilibrated inside a confocal microscope incubation chamber at 37°C with 5% CO_2_ for 1 h and imaged every 30 min to 1 h for 15 h. Fluorescence images at 1 h (A) and 15 h (B) are shown. Side panels show fluorescence channels individually. ApHID/Alexa 647 ratios and corresponding interpolated LE/Ly pH values were plotted against recording time (C). See Table S19 for statistics. (D–J) Confocal micrographs of J774 macrophage LE/Lys loaded with either 70 kDa ApHID-Alexa 647 dextrans (D–F) or 10 kDa LSyb dextrans (G–I). Side panels show fluorescence channels individually. Some cells were treated with modest concentrations of methylamine to induce subtle LE/Ly alkalinization (E and F, H and I). LSyb was excited with a 405 nm solid-state laser. Its pH-sensitive (pH-s) fluorescence band was recorded between 500 and 600 nm, whereas its pH-independent (pH-i) fluorescence band was recorded between 410 and 494 nm. ApHID/Alexa 647 and LSyb pH-s/pH-i fluorescence ratios and corresponding interpolated LE/Ly pH values were plotted for each condition (J). Experiments were repeated 3 times; 2–4 wells were imaged for each experiment, and 3–6 fields were acquired per well. For all experiments, fluorescence ratios were interpolated to pH using a calibration prepared in fixed cells. Geometrical objects and bars indicate averaged LE/Ly pH ± SEM per well (most error bars fit within the symbols). In (J) differences in pH means between conditions were assessed using the unpaired one-way ANOVA followed by Dunnett’s multiple comparison test with 95% confidence interval. *p* values shown as *p* > 0.05 (ns); *p* ≤ 0.05 (∗), *p* ≤ 0.001 (∗∗∗), *p* ≤ 0.0001 (∗∗∗∗). See Table S20 for statistics. Scale bars: 10 μm. ApH, ApHID; A647, Alexa Fluor 647; MeNH_2_, methylamine.

To confirm ApHID stability in acidic compartments, we measured LE/Ly pH reported by the probe in a primary cell type over an extended period of time. Primary murine bone marrow-derived macrophages were loaded with 70 kDa amino-dextrans labeled with ApHID and Alexa 647 and imaged for 15 h in a confocal microscope incubation chamber at 37°C in 5% CO_2_ (Figures 7A and 7B). ApHID/Alexa 647 ratios and interpolated LE/Ly pH remained stable over the course of the experiment (Figure 7C; Table S19). This demonstrates that ApHID does not undergo endolysosomal enzymatic degradation and reports pH in a stable manner over prolonged periods of time in primary cells.

To further contextualize ApHID, we used it to measure LE/Ly pH alongside a second commonly used pH sensor, LSyb (Figures 7D–7I). LSyb allows for dual ratiometric pH imaging without the need for a second pH-independent probe.^49^ We recorded LSyb pH-sensitive fluorescence between 500 and 600 nm and acquired its pH-independent signal between 410 and 494 nm. Some cells were treated with modest concentrations of methylamine in order to induce subtle LE/Ly pH alkalinization (Figures 7E and 7F, 7H and 7I). ApHID/Alexa 647 and LSyb pH-sensitive/pH-independent fluorescence ratios for all conditions were interpolated to pH (Figure 7J). ApHID detected LE/Ly alkalinization of 0.4 pH units following 4 mM methylamine treatment relative to untreated cells, and a subtle pH increase of 0.16 pH units following 8 mM treatment relative to 4 mM methylamine treatment. However, LSyb reported a LE/Ly pH of 5.67 in untreated cells, which was 0.4 pH units higher relative to ApHID. This might be due to 10 kDa dextrans being localized to endosomal compartments other than LE/Lys. Furthermore, some LSyb pH-sensitive/pH-independent ratios measured in cells treated with methylamine yielded unusually elevated pH values or could not be interpolated (Figure 7J; Table S20). This is most likely due to LSyb-poor dynamic range within the pH 5.0–6.0 window (see Figure 2C).

Overall, these results show that ApHID is able to report LE/Ly pH in a stable manner over prolonged periods of time in primary cells. Importantly, ApHID detects and reports subtle variations—within 0.2 pH units—in LE/Ly acidification induced by methylamine, in real time.

## Discussion

Herein, we report the preparation and characterization of the pH-sensitive probe ApHID*.* The chemical design of ApHID confers the molecule with robust photostability and optimal spectroscopic properties to measure pH in the acidic range of LE/Lys. ApHID contains a PEG4 chain terminated in an NHS reactive group, which increases solubility and allows for the derivatization of proteins and other molecules containing primary amines. In addition, ApHID is a weak base, whereas fluorescein, Oregon Green, and many other pH-sensitive probes are carboxylic acids. ApHID’s fluorescence increases with increasing acidity, whereas fluorescein and Oregon Green become dimmer. Also, ApHID’s dynamic range between pH 4.0 and 6.0 is superior to that of most other probes we compared it with. Overall, these properties allow for more accurate pH measurements in LE/Lys. Importantly, the pKa of ApHID and its pH-dependent fluorescence remain stable when exposed to high amounts of salt and protein as well as to ⋅OH radicals at concentrations substantially higher than those found in acidic organelles. Additionally, once incorporated into macrophage LE/Ly compartments, ApHID fluorescence remained stable for extended times, demonstrating resistance to enzymatic degradation.

It is possible to derivatize amino-dextrans with various degrees of ApHID along with pH-independent fluorophores without altering ApHID’s fluorescence properties. This is advantageous when working with difficult imaging applications such as intravital imaging, which benefits from bright markers that can withstand sustained excitation. Interestingly, in derivatized dextrans, the pKa of ApHID can be modulated by altering the ionic environment on the polymers. We found that increasing the dextran negative charge by derivatizing it with anionic molecules such as Alexa 405 increased ApHID’s pKa. This is most likely due to the stabilizing effect played by the nearby negative charge, which favors protonation of the aniline group in the ApHID core. By the same principle, adding positive charge to the polymer backbone should disfavor aniline protonation, thus requiring higher acidity to activate ApHID fluorescence and ultimately lowering ApHID’s pKa. This demonstrates that ApHID pH sensitivity can be finely tuned by modulating amino-dextran charge density, which might be advantageous when pH sensing of different acidity ranges is required.

We demonstrated that ApHID dextrans can be used to measure the pH of macrophage LE/Ly compartments in cell culture. Dextrans have been used for more than 50 years to label LE/Ly compartments. In 1978, Ohkuma and Poole measured LE/Ly pH for the first time using ratiometric fluorescein-dextran.^47^ Several subsequent studies on endosomal acidification used dextrans.^18^^,^^19^^,^^46^ We have used ApHID previously to measure LE/Ly pH in transfected HEK293 cells expressing TMEM106B mutants, using a different methodology.^17^ In the present study, we validated our measurements by multiple comparisons with other pH-sensitive probes. Differences between LE/Ly pH measured with ApHID, fluorescein, and Oregon Green were minimal and not statistically significant. The pH reported by ApHID stayed within 0.1 pH units of that reported by the other probes, indicating that they are effectively equivalent.

ApHID was sensitive to LE/Ly pH above 5.0. However, LSyb was not able to report more alkaline pH accurately, most likely due to its limited dynamic range in that pH window. Therefore, pH sensing above 5.0 using LSyb should be approached cautiously. Moreover, LSyb reported an LE/Ly pH 0.4 pH units above that reported by ApHID in untreated cells, suggesting that 10 kDa dextrans might colocalize with endosomal compartments other than LE/Lys.

Finally, a typically cumbersome aspect of pH assay protocols is the preparation of the fluorescence ratio-to-pH calibrations. Acidic buffers damage cellular membranes, and fluorescence ratios measured in fixed cells show substantial variability, which manifests in imprecise calibration curves. To circumvent that limitation, we prepared calibrations using a simplified procedure. Fluorescence ratios were measured in fixed cells incubated in pH 5.0 buffer only, and these ratios were used to build a full calibration with all subsequent ratio values corresponding to pH points 3.5–7.4 generated using titration data previously obtained in solution using a spectrophotometer. This sped up the calibration procedure and reduced calibration error, which manifested in accurate pH interpolation.

In summary, we believe that ApHID circumvents a number of limitations presented by most commercially available pH-sensitive probes. ApHID’s spectroscopic properties, pH dependence between pH 4.0–6.0, as well as its resistance to oxidation, enzymatic degradation, and photobleaching make it optimal for measuring LE/Ly pH in a variety of cell types. We believe that our methodology will prove useful in demanding imaging applications such as intravital imaging of tissues.

### Limitations of the study

Cellular acidic compartments can be imaged live using nanosensors^50^ or genetically encoded biosensors.^51^^,^^52^ The latter ensures purely lysosomal localization but requires expressing the reporters in cell culture or in animal models. Dextrans labeled with pH-sensitive probes can be used to label acidic compartments in virtually any cell type without the need for genetic manipulation. Labeling of polymers with probes and loading of LE/Ly compartments can be achieved within 2 days; 70 kDa dextrans remain inside LE/Lys for days and can be used to monitor pH for extended periods of time before being exocytosed. However, when compared with genetically encoded sensors purely expressed in lysosomes, a fraction of dextrans will accumulate in late endosomes too and report pH from a somewhat mixed endolysosomal population. Lysosomes are constantly fusing with late endosomes in a process mediated by endocytic Ca^2+^.^53^ Therefore, from a degradative point of view, LE/Lys can be regarded as a unified compartment. Nevertheless, for applications requiring pure lysosomal sensing, genetically encoded reporters may be preferable. Finally, our simplified ratio-to-pH calibration method relies on the use of dextran polymers, which are resistant to oxidation and chemical modification, and has not been validated for proteins or other biomolecules that could be labeled with ApHID.

## Resource availability

### Lead contact

Further information and requests for resources and reagents should be directed to and will be fulfilled by the lead contact, Dr. Santiago Solé-Domènech (sas2068@med.cornell.edu).

### Materials availability

Further information and requests for acidic pH indicator dye (ApHID) should be directed to and will be fulfilled by the lead contact upon request.

### Data and code availability

• Confocal microscopy stacks of images (Figures 5, 6, 7, S1, and S2) and all associated data analysis spreadsheets, as well as all supporting supplementary data, are stored at Weill Cornell Institutional Data Repository for Research (WIDRR) and will be made available by the lead contact upon request.
• This paper does not report original code.
• Any additional information required to reanalyze the data reported in this work paper is available from the lead contact upon request.

## Acknowledgments

This work was supported by the 10.13039/100007625Cure Alzheimer's Fund grant CAF-211540-02 and 10.13039/100000002NIH grants RF1-AG078244 and R01-HL093324. S.S.D. was supported by the Swedish Research Council International Postdoctoral Fellowship number 637-2013-503/D0050301 and the Leon Levy Foundation Fellowship in Neuroscience. The authors are grateful to Weill Cornell Chemistry Core for synthesizing ApHID, Raksha Narendra for assistance with cell culture and dextran derivatization, Warren Zipfel at Cornell University for guidance on quantum yield measurements, and Diane Del Valle at Mount Sinai for advice in preparing the graphical abstract.

## Author contributions

Conceptualization, F.R.M., S.S.-D., P.K.S., and J.D.W.; methodology, S.S.-D., P.K.S., F.R.M., L.F., J.D.W., and C.J.M.; validation, S.S.-D. and P.K.S.; investigation, S.S.-D., P.K.S., and C.-I.J.M.; resources, F.R.M., S.S.-D., J.D.W., C.-I.J.M., and L.F.; data curation, S.S.-D. and P.K.S.; writing – original draft, S.S.-D., F.R.M., and J.D.W.; writing – review & editing, S.S.-D., F.R.M., and J.D.W.; visualization, S.S.-D.; supervision, F.R.M. and J.D.W.; project administration, F.R.M., S.S.-D., and J.D.W.; funding acquisition: F.R.M., S.S.-D., and J.D.W.

## Declaration of interests

The chemical synthesis and uses of the pH-sensitive probe ApHID have been included and described in a pending patent application, for which S.S.-D., P.K.S., J.D.W., and F.R.M. are co-inventors.

## STAR★Methods

### Key resources table

**Table:** 

REAGENT or RESOURCE	SOURCE	IDENTIFIER
**Chemicals, peptides, and recombinant proteins**		

Sodium hydroxide	Sigma-Aldrich	S5881
Sodium phosphate monobasic anhydrous	Sigma-Aldrich	S8262
Calcium chloride dihydrate	Sigma-Aldrich	C5080
Magnesium chloride hexahydrate	Sigma-Aldrich	102510804
Sodium acetate anhydrous	Sigma-Aldrich	58750
Sodium phosphate dibasic heptahydrate	Sigma-Aldrich	59390
Citric acid anhydrous	Sigma-Aldrich	C4540
Sodium citrate trisodium salt dihydrate	Sigma-Aldrich	S4641
Trizma Maleate	Sigma-Aldrich	T3128
Trizma Base	Sigma-Aldrich	T1503
Trizma Hydrochloride	Roche	10812846001
Sodium bicarbonate	Sigma-Aldrich	S5761
HEPES	Sigma-Aldrich	H3375
Sodium chloride	Sigma-Aldrich	S9625
Potassium chloride	Sigma-Aldrich	P5405
Iron (II) perchlorate hydrate	Sigma-Aldrich	334081
Hydrogen peroxide 30% solution	VWR	BDH7690-3
Bovine serum albumin	Sigma-Aldrich	A2153
Paraformaldehyde 32% solution	Electron Microscopy Sciences	15714-S
Monensin Sodium Salt	Sigma-Aldrich	M5273
Nigericin Sodium Salt	Sigma-Aldrich	N7143
Methylamine Hydrochloride	Sigma-Aldrich	M0505
*N*-[(Dimethylamino)-1*H*-1,2,3-triazolo-[4,5-*b*]pyridin-1-ylmethylene]-*N*-methylmethanaminium hexafluorophosphate *N*-oxide (HATU)	Sigma-Aldrich	445460
Ethylamine (2M in THF)	Sigma-Aldrich	395072
NH_2_-PEG_4_-COOH	Sigma-Aldrich	QBD10244
EDC	Sigma-Aldrich	39391
*N*-hydroxysuccinimide	Sigma-Aldrich	130672
Sodium sulfate, anhydrous	Sigma-Aldrich	239313
Dimethylformamide	Sigma-Aldrich	227056
Methanol	Sigma-Aldrich	439193
Triethylamine	Sigma-Aldrich	471283
Dichloromethane	Sigma-Aldrich	650463
Dimethylsulfoxide-*d*_*6*_	Sigma-Aldrich	151874
Tetrahydrofuran	Sigma-Aldrich	401757
Acetonitrile	Sigma-Aldrich	AX0156
Formic Acid	Sigma-Aldrich	5330020050
NHS Alexa 405	Thermo Fisher	A30000
NHS Alexa 647	Thermo Fisher	A20106
NHS Cy5⋅3xSO_3_^–^ (Cy5 SE TRI SO3)	AstaTech	44193
NHS Acidic pH Indicator Dye (ApHID)	Custom-made	N/A
NHS 5/6-carboxyfluorescein	Thermo Fisher	46410
NHS Oregon Green	Thermo Fisher	O6147
LysoSensor™ yellow/blue 10 KDa	Thermo Fisher	L22460
TFP ester pHrodo™ Deep red	Thermo Fisher	P35359
NHS BioTracker™ Orange	Sigma-Aldrich	SCT214
Amino Dextran polymer 10 kDa	Thermo Fisher	D1860
Amino Dextran polymer 70 kDa	Thermo Fisher	D1862
Amino Dextran polymer 70 kDa	Fina Biosolutions	AD70x33
Dubbelco’s Modified Eagle Medium	Corning	15-013-CV
Dubbelco’s Modified Eagle Medium (no phenol red)	Corning	90-013-PB
Fetal Bovine Serum (FBS)	Gemini	100–106
l-glutamine	Gibco	25030081
d-(+)-glucose	Sigma-Aldrich	G7021
Sodium pyruvate	Sigma-Aldrich	S8636
Penicillin-Streptomycin	Thermo Fisher	15140163

**Deposited data**		

Raw and analyzed data	This paper	WCM Institutional Data Repository for Research (WIDRR)

**Experimental models: Cell lines**		

J774A.1 Murine macrophages	ATCC	J774A.1 (TIB-67)
Primary murine bone-marrow derived macrophages	Laboratory of Dr. Frederick Maxfield	N/A

**Experimental models: organisms/strains**		

C57BL/6J	Jackson Laboratories	000664

**Software and algorithms**		

MetaMorph v.6.7.1 for Windows	Molecular Devices	www.moleculardevices.com
GraphPad Prism v.10.3.1 for Windows	GraphPad Software	www.graphpad.com
FIJI (ImageJ v.1.54f) for Windows	Schindelin et al.^54^	https://imagej.net/software/fiji
Inkscape v.1.2.2.	Inkscape Project	https://inkscape.org
ChemDraw v. 23.1.2.	Revvity Signals Software	https://revvitysignals.com/products/research/chemdraw
Mnova v. 14.3.2	Mestrelab Research	https://mestrelab.com/main-product/mnova

**Other**		

3.5 KDa Side-A-Lyzer dialysis cassettes	Thermo Fisher	66330
20 KDa Side-A-Lyzer dialysis cassettes	Thermo Fisher	66003
Ultra-pure distilled water	Hydro Services	PicoPure3 System
Filtration cups, 0.2 μm aPES (0.5 L)	Thermo Fisher	595–3320
Filtration cups, 0.2 μm aPES (1 L)	Thermo Fisher	597–4520
Spectrophotometer/plate reader	Molecular Devices	SpectraMax M3
384-well polystyrene microplates	Corning	3746
Gravity convection oven (Isotemp)	Fisher Scientific	15-103-0503
Confocal microscope	Zeiss	LSM 880
Confocal microscope	Leica	Stellaris
Power Meter	Coherent	LaserMate Q

### Experimental model and study participant details

#### J774A.1 murine macrophage cell culture

J774A.1 murine macrophages (ATCC TIB-67) were grown in Dulbecco’s Modified Eagle’s Medium (DMEM) containing 4.5 g/L glucose and 1 mM sodium pyruvate (Corning 15-013-CV), with 10% fetal bovine serum (FBS, Gemini BenchMark FBS 100–106), 4 mM L-glutamine (Gibco 25030081), and 1% penicillin-streptomycin (Thermo Scientific 15140163) in an incubator at 37°C with humidified atmosphere and 5% CO_2_. Cells were passed at a subcultivation ratio of 1:5 every 2–3 days.

#### Murine bone marrow-derived macrophage extraction and culture

Murine bone marrow-derived macrophages (BMMs) were cultured as described previously with minor modifications.^55^^,^^56^ Briefly, bone marrow was extracted and spun out of sterilized femurs from 8 to 12 weeks old wildtype adult C57BL/6J male mice, into cold DMEM at 8,000 x g. 3 x 10^6^ bone marrow cells were resuspended and differentiated for 7 days in a non-treated 10 cm culture dish with DMEM medium supplemented with 10% (v/v) heat-inactivated FBS, 1% (v/v) penicillin/streptomycin, 2 mM L-glutamine and 20% (v/v) L-929 cell conditioned medium in a humidified atmosphere (5% CO_2_) at 37°C.

#### Mouse models

Wild-type adult mice (Jackson Laboratories, stock# 000664) were used in this study to prepare BMMs. All mice were maintained on a C57BL/6J background. Mice were housed for harem breeding when necessary (one male, two females) and maintained in 12-h dark/light cycle sterile ventilated cages with access to food and water *ad libitum* at Weill Cornell Medicine animal facilities. All animal experiments were conducted in compliance with the Institutional Animal Care and Use Committee of Weill Cornell Medicine.

### Method details

#### Preparation of reagents and dextrans

See Table S3 for a list of dextrans, concentrations, fluorophore labeling, and reaction conditions used for each experiment described below.

##### Dextran derivatization with fluorophores

Polymers of 10 KDa (Thermo Fisher D1860) or 70 kDa amino-dextrans (Thermo Fisher D1862 or Fina Biosolutions AD70x33) were solubilized at various concentrations in sterile 0.1 M NaHCO_3_ buffer adjusted to pH 8.3 and reacted with the following *N*-hydroxysuccinimidyl esters (NHS): NHS-ApHID (custom-made), NHS-5/6-carboxyfluorescein (NHS-fluorescein, Thermo Fisher 46410), NHS-Oregon Green (Thermo Fisher O6147), NHS-BioTracker Orange (Sigma-Aldrich SCT214), TFP-pHrodo Deep red (Thermo Fisher P35359), NHS-Alexa 405 (Thermo Fisher A30000), NHS-Cy5⋅3xSO_3_^–^ (AstaTech 44193) or NHS-Alexa 647 (Thermo Fisher A20106) at various polymer:dye molar ratios (see Table S3) for 1–2 h at room temperature (RT) with constant rotation. LysoSensor™ yellow/blue attached to 10 kDa dextrans was commercially available (Thermo Fisher L22460). To examine the effect of various degrees of ApHID derivatization or the presence of negatively charged fluorophores on pKa we used 70 kDa amino-dextrans from Fina Biosolutions, reacted with a fixed amount of NHS-Alexa 405 or NHS-Cy5⋅3xSO_3_^–^, followed by reaction with varying molar ratios of NHS-ApHID. For studies on the effect of charge density on ApHID pKa, the polymers were reacted with NHS-ApHID first, followed by aliquoting and reaction with various amounts of NHS-Alexa 405 to add negative charge density to the polymers. For ratiometric pH imaging experiments in live cells, we used 70 kDa amino-dextrans from Thermo Fisher, reacted with NHS-ApHID, NHS-fluorescein or NHS-Oregon Green and NHS-Alexa 647 at 4:3 molar ratio. Following reaction, dextrans were purified by extensive dialysis in 3.5 KDa or 20 KDa cutoff Side-A-Lyzer dialysis cassettes (Thermo Fisher 66330 and 66003) against 1X PBS.

##### Measuring fluorophore incorporation into amino-dextrans by absorbance

When measuring ApHID incorporation, dextrans were diluted in 25 mM citric acid, 25 mM sodium citrate buffer adjusted to pH 3.0 and absorbance was read at 502 nm. When measuring labeling with fluorescein or Oregon Green, the dextrans were diluted in pH 7.4 PBS buffer and absorbance was read at 490 nm. When measuring labeling with Alexa 405, Cy5⋅3xSO_3_^–^, or Alexa 647, the dextrans were diluted in PBS buffer and absorbance was read at 402, 666, or 655 nm, respectively. All dextrans were diluted to 0.02 mg/mL in buffers. Measurements were done using the quartz cuvette reader on a SpectraMax M3 spectrophotometer (Molecular Devices). The extent of dextran labeling was lower than the polymer:dye ratio of the reaction mixture; dye incorporation efficiencies ranged from 25 to 50% (see Table S3).

##### Buffers for measurements in solution

Dextrans were solubilized in buffers with pH adjusted between 1.5 and 8.5. To prepare the buffers, buffer salts were added to 0.66X PBS as follows: *pH 1.5 to 3.5 buffer:* 25 mM citric acid plus 25 mM sodium citrate; *pH 4.0 to 5.5:* 50 mM TRIS-maleate; *pH 6.0-7.0 buffer:* 50 mM sodium phosphate monobasic anhydrous; *pH 7.5:* 25 mM TRIS hydrochloride plus 25 mM sodium phosphate dibasic; *pH 8.0-8.5:* 50 mM TRIS base. Buffer pH was measured using an Orion Star A211 pH meter (Thermo Fisher), and acidity was adjusted by adding 1–3 N HCl or 1–10 N NaOH dropwise. The resulting ionic strength of the buffer solutions was approximately 150 mM. After preparation, the buffers were filtered through a 0.4 μm filter membrane and stored at 4°C.

##### Buffers and cell media for measurements in fixed and live cells

Fixed cells were imaged in buffers prepared by adding buffer salts to 0.13X PBS containing 10% FBS. The buffers also contained 40 mM sodium acetate, 40 mM methylamine hydrochloride and 40 μM monensin as membrane-permeant equilibrators (to ensure buffer equilibration across membranes). The pH of the complete buffer solutions was adjusted as described above. The resulting ionic strength of the solutions was approximately 150 mM. Buffers were filtered and stored at 4°C. Live cells were imaged in DMEM without phenol red (Corning 90-013-PB) with 10% FBS, 2.2 g/L sodium bicarbonate (Sigma S5761), 4 mM L-glutamine, 1 mM sodium pyruvate (Sigma-Aldrich S8636) and 1% penicillin-streptomycin.

#### pH-dependent absorbance measurements in solution

To measure pH-dependent absorbance spectra for ApHID, 10 kDa amino-dextrans derivatized with the probe were diluted to 0.04 mg/mL in pH-adjusted buffers (see preparation of buffers above) and loaded into 384-well flat clear-bottom black polystyrene microplates (Corning, 3746). Three wells were loaded per pH buffer condition and dye. The microplates were centrifuged at 3700 rpm for 1 min, followed by absorbance spectra acquisition between 400 and 600 nm at RT using a SpectraMax M3 spectrophotometer. Absorbance was read from the top of the plate. To record pH-dependent absorbance titrations for all probes, derivatized 10 kDa amino-dextrans were diluted to 0.02 mg/mL in pH-adjusted buffers, followed by absorbance reading at 500 nm (ApHID); 495 nm (fluorescein); 505 nm (Oregon Green); and 381 nm (LysoSensor yellow/blue). For both measurements, absorbance measured from buffers alone was subtracted as blank.

#### pH-dependent fluorescence measurements in solution

To record pH-dependent emission spectra for ApHID, 10 kDa amino-dextrans derivatized with the probe were diluted to 0.04 mg/mL in pH-adjusted buffers and loaded into 384-well microplates. ApHID was excited at 480 nm, and emission spectra were collected between 500 and 600 nm using a SpectraMax M3 spectrophotometer. Fluorescence was read from the bottom of the plate. To record pH-dependent emission titrations, 10 kDa or 70 kDa amino-dextrans labeled with the various probes were diluted to 0.02 mg/mL in pH-adjusted buffers, followed by excitation and fluorescence measurement at ex/em 500/520 nm (ApHID); 490/525 nm (fluorescein); 505/525 nm (Oregon Green); 381/450 nm (LysoSensor™ yellow/blue,pH-independent) and 381/521 nm (pH-sensitive); 532/565 nm (BioTracker™ Orange); 626/655 nm (pHrodo™ Deep red); 402/425 nm (Alexa 405); 620/666 nm (Cy5⋅3xSO_3_^–^) and 620/655 nm (Alexa 647). Emission measured from buffers alone was subtracted as blank. Measurements were repeated at least twice and carried out at RT.

#### Quantum yield and extinction coefficient measurements in solution

ApHID quantum yield (φf_unknown_) was determined following a previously published protocol.^57^ Stock solutions of NHS-ApHID and NHS-fluorescein were prepared in 1X PBS and allowed to hydrolyze overnight at RT. Four series of dilutions of ApHID (each in a different buffer) were prepared in buffers adjusted to pH 3.5 to 6.0. Four dilutions of fluorescein, with a known quantum yield (φf_standard_) of 0.95, were prepared in 0.1 M NaOH as a standard reference. For each dilution and buffer, absorbance and emission spectra were recorded in the range of 400–550 nm and 500–600 nm, respectively, using 1 cm quartz cuvettes and the cuvette reader in a SpectraMax M3 spectrophotometer. Measurements in buffers alone were subtracted as blank. Integrated fluorescence was plotted against integrated absorbance for each dilution and buffer, and the curves were fit to a linear trend. Measurements were repeated twice. Quantum yields for ApHID, for each buffer pH, were calculated using the equation below:

$$\varphi_{f_{unknown}}=\varphi_{f_{standard}}\left(\frac{m_{unknown}}{m_{standard}}\right)$$

Where φf refers to quantum yield, and *m*_*unknown*_ and *m*_*standard*_ are the slopes of the resulting linear regressions for ApHID and fluorescein dilutions, respectively.

To determine the extinction coefficient (ε) of ApHID, several dilutions of a hydrolyzed NHS-ApHID stock were prepared in pH 3.0 citric acid, sodium citrate buffer, and their absorbance at 492 nm was measured in quartz cuvettes using a SpectraMax M3 spectrophotometer. Absorbance measurements in buffers alone were subtracted as blank. The corrected absorbance was fit to Beer’s law, A = ε·b·c, where A is the absorbance, ε is the molar extinction coefficient, b is the path length of the cuvette and c is the concentration. Measurements were repeated twice.

#### Hydroxyl radical (⋅OH) generation and oxidation assay in solution

500 μM NHS-ApHID, NHS-fluorescein, and NHS-Oregon Green solutions were prepared in 1X PBS and allowed to hydrolyze overnight. The probes were then diluted into 10 μM aliquots in 1X PBS and refrigerated in ice. Stock solutions of 50 mM Fe(OCl)_2_ (Sigma-Aldrich 334081) and 50 mM H_2_O_2_ (VWR, 30% aqueous stock, BDH7690-3) were prepared in ultra-pure water and immediately added to the fluorophore aliquots to a final concentration of 100 or 200 μM. Plastic tubes containing the aliquots were vortexed for 10 s, sealed in paraffin, and incubated at 37°C in a convection oven with constant rotation for 20 h. At the end of the incubation time, the aliquots were diluted 1:200 in buffers with pH adjusted between 3.5 and 7.5 and loaded into 384-well microplates. Fluorescence was measured for each probe and buffer at RT (see ex/em wavelenghts above) using a SpectraMax M3 spectrophotometer. The experiment was repeated twice.

#### Effect of salt and protein on probe fluorescence and pKa in solution

10 kDa amino-dextrans derivatized with the various NHS-probes were diluted to 0.02 mg/mL in pH-adjusted buffers additionally supplemented with either 1 mM CaCl_2_ and 1 mM MgCl_2_, sodium acetate (replacing sodium chloride), or 50 mg/mL bovine serum albumin (BSA). pH-adjusted buffers lacking sodium chloride were prepared using a 0.66X PBS base for which sodium chloride had been replaced by sodium acetate. Buffers containing 50 mg/mL BSA were prepared in 1X PBS and adjusted to pH 2.5–7.0. Dextran dilutions in buffers were vortexed and loaded into 384-well microplates, sealed using paraffin and incubated at 37°C in a convection oven for 20 h. After incubation, probe fluorescence was measured at RT (see ex/em wavelenghts above) using a SpectraMax M3 spectrophotometer. The experiment was repeated twice.

#### ApHID cytotoxicity assay in cell culture

*J774 macrophages were seeded* at 30,000 cells/well in 96-well plates with transparent polymer bottoms (Cellvis, P96-1.5P) and incubated with 70 kDa dextrans from Thermo Fisher labeled with NHS-ApHID and NHS-Alexa 647 at a dextran concentration of 0.5 mg/mL in complete DMEM media, or left untreated overnight. In the morning, cells were chased for 4 h in fresh DMEM media and incubated with 1 μg/mL Hoechst 33342 (Cayman Chemical, 15547) in complete DMEM inside a confocal microscope incubation chamber at 37°C and 5% CO_2_ for 1 h followed by confocal microscopy imaging. The experiment was repeated three times; 3–4 wells were imaged per experiment and condition and 16 fields were acquired per well.

#### Photostability studies in J774 macrophages using confocal microscopy

ApHID, fluorescein, and Oregon Green were compared in fixed cells. J774A.1 murine macrophages were seeded in 35-mm dishes with central 7 mm diameter glass bottom imaging chambers coated with poly-d-lysine at 10,000 cells per chamber and allowed to settle for 1–2 h at 37°C. Once settled, cells were incubated with 70 kDa amino-dextrans from Thermo Fisher labeled with NHS-ApHID, NHS-fluorescein, or NHS-Oregon Green at a final concentration of 1 mg/mL in complete DMEM overnight. The following morning, cells were washed once and chased for 3 h in fresh DMEM, followed by 5 min fixation in 0.5% paraformaldehyde (PFA) and three washes in 1X PBS. Immediately prior to imaging, cells labeled with ApHID or Oregon Green dextrans were incubated in TRIS maleate pH 5.0 buffer, and cells labeled with fluorescein dextran were incubated in pH 7.4 1X PBS buffer, for 5 min at 37°C inside a confocal microscope incubation chamber. The buffers were supplemented with 40 mM methylamine hydrochloride, 40 mM sodium acetate, 2.5 μM nigericin and 2.5 μM monensin as membrane-permeant equilibrators to facilitate buffer equilibration across cell membranes. Following buffer equilibration, cells were immediately imaged by confocal microscopy at 37°C (see “photobleaching study in fixed cells” in the “confocal microscopy” section). The experiment was repeated twice. 2 dishes per fluorophore condition were imaged, and 4 fields were acquired for each dish. Additionally, ApHID and LysoSensor yellow/blue (LSyb) photostabilities were compared in live cell culture. J774 macrophages were seeded at 40,000 cells/well in Cellvis 96-well plates and incubated with 70-kDa amino-dextran labeled with NHS-ApHID or 10-kDa LSyb-dextran at 0.5 or 2 mg/mL in complete DMEM overnight, respectively, followed by a 4 h chase in the morning. Prior to imaging, the media in the plates was replaced by complete DMEM without phenol red and the plates were allowed to equilibrate in a confocal microscope incubation chamber for at least 1 h at 37°C and 5% CO_2_, followed by confocal imaging (see “photobleaching study in live cells” in the “confocal microscopy” section). Experiments were repeated three times. Two wells were imaged per condition, and three fields were acquired per well.

#### Ratiometric imaging of buffer equilibration kinetics vs. pH in fixed J774 macrophages

J774A.1 murine macrophages were seeded in Cellvis 96-well plates at 40,000 cells per well and allowed to settle for 1–2 h in the incubator at 37°C. Once settled, cells were incubated with 70 kDa amino-dextrans from Thermo Fisher labeled with NHS-ApHID and NHS-Alexa 647 at a dextran concentration of 0.5 mg/mL in complete DMEM overnight. The following morning, cells were washed twice and chased in fresh DMEM for at least 3 h. Next, the cells were fixed in 0.5% PFA for 5 min and washed 3x in 1X PBS, and the plate was then transferred to a confocal microscope incubator and allowed to equilibrate at 27°C for at least 20 min followed by image acquisition (time 0). After that, the PBS buffer was carefully removed from the wells using a 200 μL pipette followed by the addition of 200 μL of buffers with pH adjusted to 4.0, 4.5, 5.0, 5.5, and 6.0 (see main STAR Methods section) supplemented with 10% FBS, 40 mM methylamine hydrochloride, 40 mM sodium acetate, and 40 μM monensin (buffers had been pre-warmed to 27°C prior to addition to cells). The cells were allowed to equilibrate in the buffers for 5 min, and regions of interest were then acquired every 5 min for a period of 2 h. 2 wells were imaged for each buffer condition, and 3 fields were acquired per well. The experiment was repeated three times. ApHID/Alexa 647 ratios were calculated as described in the “digital image analysis” section of the STAR Methods and plotted against incubation time for each buffer condition. Data were fit to rectangular hyperbolae for visualization purposes.

#### Ratiometric imaging of buffer equilibration kinetics vs. monensin concentration in J774 macrophages

Cells were seeded in 96-well plates and incubated with 70 kDa amino-dextrans labeled with NHS-ApHID and NHS-Alexa647 at a dextran concentration of 0.5 mg/mL in complete DMEM overnight. The following morning, cells were chased for 4 h in complete DMEM media and fixed in 0.5% PFA for 5 min and washed 3x in 1X PBS. The plate was then transferred to a confocal microscope incubation chamber and allowed to equilibrate at 37°C for at least 20 min followed by image acquisition (time 0). After that, the PBS buffer was carefully removed from the wells using a 200 μL pipette followed by the addition of 200 μL of 50 mM TRIS maleate pH 5.0 buffer supplemented with 10% FBS, 40 mM methylamine hydrochloride, 40 mM sodium acetate, and 5, 40, or 80 μM monensin, pre-warmed at 37°C prior to addition. The cells were allowed to equilibrate in the buffers for 6 min, and regions of interest were acquired every 5 min for a period of 40 min. Two wells were imaged for each buffer condition, and 3 fields were acquired per well. The experiment was repeated three times. ApHID/Alexa 647 ratios were calculated as described in the “digital image analysis” section of the STAR Methods and plotted against incubation time for each buffer condition. Data were fit to rectangular hyperbolae for visualization purposes.

#### Fluorescence ratios-to-buffer pH calibration in fixed J774 macrophages

J774A.1 murine macrophages were seeded in Cellvis 96-well plates at 40,000 cells per well and allowed to settle for 1–2 h in the incubator at 37°C. Once settled, cells were incubated with 70 kDa amino-dextrans from Thermo Fisher labeled with NHS-ApHID and NHS-Alexa 647 at a dextran concentration of 0.5 mg/mL in complete DMEM overnight. The following morning, cells were washed twice and chased in fresh DMEM for at least 3 h. Next, the cells were fixed in 0.5% PFA for 5 min and washed 3x in 1X PBS, and the plate was then transferred to a confocal microscope incubation chamber and allowed to equilibrate to 37°C for at least 20 min, followed by image acquisition (time 0). After that, the PBS buffer was carefully removed from the wells using a 200 μL pipette and 200 μL of buffers with pH adjusted to 4.0, 4.5, 5.0, 5.5, and 6.0 (see main STAR Methods section) were added. The buffers were supplemented with 10% FBS, 40 mM methylamine hydrochloride, 40 mM sodium acetate and 40 μM monensin and had been pre-warmed to 37°C prior to addition. The cells were allowed to equilibrate in the buffers for 20 min (pH 4.0 and pH 4.5) or at least 30 min (pH 5.0–6.0) followed by image acquisition. Two wells were imaged for each buffer condition, and 3 fields were acquired per well. The experiment was repeated three times.

#### Live ratiometric LE/Ly pH imaging of macrophage cell lines

J774A.1 murine macrophages or bone marrow-derived macrophages (BMMs) were seeded in Cellvis 96-well plates at 40,000 cells per well and allowed to settle for 1–2 h in the incubator at 37°C. Once settled, cells were incubated with 70 kDa amino-dextrans from Thermo Fisher labeled with NHS-ApHID, NHS-fluorescein or NHS-Oregon Green (pH-sensitive), and NHS-Alexa 647 (pH-independent) at a dextran concentration of 0.5 mg/mL in complete DMEM overnight. For side-by-side pH imaging using ApHID or LysoSensor yellow/blue (LSyb), J774 macrophages were incubated with 70 kDa amino-dextrans labeled with NHS-ApHID and NHS-Alexa 647 at 0.5 mg/mL or 10 kDa LSyb-dextran at 2 mg/mL in complete DMEM media. The following morning, cells were washed twice and chased in fresh DMEM for at least 2 h, followed by 1 h equilibration in DMEM without phenol red. Plates were then transferred to a confocal microscope incubation chamber and allowed to equilibrate at 37°C with 5% CO_2_ for at least 20 min prior to imaging. Some cells were treated with 4, 8 or 20 mM methylamine hydrochloride concentrations for 10 min prior to imaging to induce LE/Ly alkalinization. For continuous imaging experiments, regions of interest were acquired from each well every 5, 30 or 60 min, for 1 h–15 h. For pH calibration, two wells for each fluorophore pair were fixed in 0.5% PFA for 5 min and washed three times in 1X PBS, followed by incubation in 50 mM TRIS maleate pH 5.0 buffer supplemented with 10% FBS, 40 mM methylamine hydrochloride, 40 mM sodium acetate and 40 μM monensin, at 37°C for 20–30 min inside a confocal microscope incubation chamber, and imaged immediately after. The resulting fluorescence ratios corresponding to pH 5.0 for each probe were used to generate full calibrations using titration data previously acquired in solution using a spectrophotometer (see pH-dependent fluorescence measurements in solution section above). pH imaging experiments were repeated 2–3 times. Two to three wells were imaged for each condition and probe pair, and four fields were acquired for each well.

#### Confocal microscopy

##### Ratiometric imaging of buffer equilibration kinetics

Imaging of fixed J774 macrophages was done using a Stellaris confocal microscope (Leica Systems) with a 40× air objective (0.95 NA) and the pinhole adjusted to 1 Airy unit. ApHID and Alexa 647 were excited using a white light solid-state laser adjusted to 495 nm and 650 nm, respectively. Fluorescence was detected using high-sensitivity silicon-based HyD detectors with a spectral window adjusted to collect light between 500-550 nm and 660–720 nm for ApHID and Alexa 647, respectively. Stacks of images with 1.5 μm separation in the vertical axis were acquired for each field imaged.

##### ApHID cytotoxicity assay in cell culture

J774 macrophages stained with Hoechst were imaged live in complete DMEM without phenol red using a Leica Stellaris confocal microscope equilibrated at 37°C with 5% CO_2_. Images were acquired using a 20× air objective (0.75 NA) with the pinhole adjusted to 1 Airy unit. Hoechst 33342 was excited using a 405 nm solid-state laser, and fluorescence was detected using a high-sensitivity silicon-based HyD detector with a spectral window adjusted to collect light between 415 and 500 nm. Stacks composed of 20 images with 1 μm separation in the vertical axis were acquired for each field imaged.

##### Photobleaching study in fixed cells

J774 macrophages were imaged using an LSM 880 confocal microscope (Zeiss) at 37°C. Fluorophores were excited using a 35 mW 488 nm argon laser with a digital power output adjusted to 30%, yielding 5 μW of power at the front element of the 40× objective (1.30 NA) used for imaging, as determined with an external laser power meter. Single cell planes were irradiated for 0.5 s per cycle (50 cycles in total) with 1 s intervals between irradiation pulses. Fluorescence was detected using a high-sensitivity Zeiss GaAs detector with a spectral window adjusted to collect light at 500–550 nm. Pixel dwell time was 0.33 μs.

##### Photobleaching study in live cells

J774 macrophages were imaged with a Stellaris confocal microscope. Images were acquired using a 20× air objective (0.75 NA) with the pinhole adjusted to 1 Airy unit. ApHID was excited using a white light solid-state laser adjusted to 488 nm whereas LysoSensor yellow/blue (LSyb) was excited using a 405 nm solid-state laser. The power output was adjusted to 5 μW for both lasers using an external laser power meter. Single cell planes were irradiated for 0.6 s per cycle (50 cycles in total) with 8 s intervals between irradiation pulses. Fluorescence was detected using high-sensitivity silicon-based HyD detectors with a spectral window adjusted to collect light between 500 and 600 nm for both probes. Stacks of images with 1 μm separation in the vertical axis were acquired for each field imaged. Pixel dwell time was 0.33 μs.

##### Live ratiometric LE/Ly pH imaging of macrophage cell lines

Imaging of fixed cells or live cells was done with a Stellaris confocal microscope (Leica). Images were acquired using the 20× (0.75 NA) or 63× (1.4 NA) air objectives with pinhole adjusted to 1 Airy unit. ApHID, fluorescein and Oregon Green were excited using a white light solid-state laser adjusted to 495 nm, and Alexa 647 was excited with the laser adjusted to 650 nm. LSyb was excited using a 405 nm solid-state laser. In pH assays comparing ApHID, fluorescein and Oregon Green, fluorescence was detected using high-sensitivity silicon-based HyD detectors with a spectral window adjusted to collect light between 500-550 nm and 660–720 nm for all green-emitting dyes and Alexa 647, respectively. In experiments comparing ApHID and LSyb, pH-dependent fluorescence was acquired between 500 and 600 nm for both probes. For LSyb, pH-independent fluorescence was acquired between 410 and 494 nm. Stacks of images with 0.75–1.5 μm separation between planes in the vertical axis were acquired for each field imaged.

#### Chemical synthesis and materials processing

Unless otherwise stated, all commercially available materials were purchased from Sigma-Aldrich and were used without further purification. The key resources table includes a list of reagents and equipment used in ApHID’s synthetic procedures. The Methods S1 includes the chemical structures of the known precursors as well as those of the new compounds synthesized in this study. When necessary, solvents and reagents were dried prior to use, using standard protocols. All non-aqueous reactions were carried out in oven-dried glassware under an atmosphere of Argon. ^1^H and ^13^C NMR spectra were acquired on a Bruker Avance III HD spectrometer at 500 MHz for ^1^H and 125 MHz for ^13^C and are included in Data S1. Chemical shifts are expressed in parts per million downfield from tetramethylsilane (TMS), using either TMS or the solvent resonance as an internal standard (TMS, ^1^H: 0 ppm; chloroform, ^13^C: 77.0 ppm; DMSO-d_6_, ^1^H: 2.5 ppm; ^13^C: 39.5 ppm). Data are reported as follows: chemical shift, multiplicity (s = singlet, d = doublet, t = triplet, q = quartet, m = multiplet, br = broad), integration, and coupling constant. LC-MS analysis using a Waters I-Class ACQUITY UPLC system equipped with an ACQUITY Photodiode Array (PDA), a Waters SQD2 mass spectrometer, and a Waters ACQUITY BEH C18 column (1.7 μm, 2.1 × 100 mm). The solvent system consisted of 0.1% formic acid in water (solvent A) and 0.1% formic acid in acetonitrile (solvent B). Flow was set to 0.3 mL/min, and a gradient of 5%–95% solvent B was applied over a period of 3 min. The total run time was 4 min. Eluents were detected using a PDA at a wavelength of 254 nm. Mass data were obtained in both positive and negative electrospray mode at a cone voltage of 30 V. HPLC purifications were performed using a Waters AutoPure HPLC/MS system equipped with XBridge OBD prep C18 5μm (19 × 150 mm) column and SQD2 mass spectrometer.

##### Synthesis of PKS8324

PKS8323 (2.00 g, 2.98 mmol, 2⋅TEA salt) was dissolved in DMF (20 mL), and the solution was cooled to 0°C. *N*-[(dimethylamino)-1*H*-1,2,3-triazolo-[4,5-*b*]pyridin-1-ylmethylene]-*N*-methylmethanaminium hexafluorophosphate *N*-oxide (HATU) (1.13 g, 2.98 mmol) was added to the solution at 0°C. After stirring for 5 min at 0°C, ethylamine (2M in THF, 9.0 mmol, 4.5 mL) was added, and the mixture was allowed to warm to room temperature slowly. The reaction yielded a mixture of the starting material, the desired product, and the diamide. The mixture was purified using a CombiFlash (silica gel) with a gradient of 0–10% methanol (0.5% Et_3_N) in DCM (0.5% Et_3_N), yielding the product (5 eq. triethylamine by NMR; 2.47 g, 83%) as a red solid. ^1^H NMR (500 MHz, DMSO-*d*_*6*_) δ 1.05 (t, *J* = 7.2 Hz, 3H), 1.43 (s, 3H), 1.66 (s, 3H), 2.31 (s, 3H), 2.51 (s, 3H), 2.69 (s 3H), 2.71 (s, 6H), 3.14–3.22 (m, 2H), 7.09 (d, *J* = 8.0 Hz, 1H), 7.10 (s, 1H), 7.17 (d, *J* = 8.0 Hz, 1H), 8.12 (t, *J* = 5.5 Hz, 1H), 10.34 (br, 1H).

##### Synthesis of PKS8325

PKS8324 (2.47 g, 2.47 mmol, 5 eq TEA) and HATU (971 mg, 2.55 mmol) were dissolved in DMF (15 mL) under an argon atmosphere. The solution was cooled to 0°C, and NH_2_-PEG_4_-COOH (645 mg, 2.43 mmol) was added. After stirring for 5 min at 0°C, Triethylamine (4.87 mmol, 680 μL) was added. The reaction mixture was allowed to warm to room temperature and stirred overnight. After completion of the reaction, the solvent was evaporated, and the mixture was purified using a CombiFlash (silica gel) with a gradient of 0–30% methanol (0.5% Et_3_N) in DCM (0.5% Et_3_N)] to give the product (1.21 g, 67%) as a red-brown solid. ^1^H NMR (500 MHz, DMSO-*d*_*6*_) δ 1.05 (t, *J* = 7.2 Hz, 3H), 1.43 (s, 3H), 1.44 (s, 3H), 2.32 (s, 3H), 2.34 (t, *J* = 6.6 Hz, 2H), 2.50 (s, 6H), 2.71 (s, 6H), 3.16–3.19 (m, 2H), 3.22–3.25 (m, 2H), 3.29–3.35 (m, 14H), 3.56 (t, *J* = 6.6 Hz, 2H), 7.07 (d, *J* = 8.1 Hz, 1H), 7.08 (s, 1H), 7.18 (d, *J* = 8.1 Hz, 1H), 8.11 (t, *J* = 6.0 Hz, 1H), 8.13 (t, *J* = 5.3 Hz, 1H).

##### Synthesis of PKS8326

PKS8325 (1.21 g, 1.65 mmol) and EDC (575 mg, 3.00 mmol) were dissolved in DCM (50 mL) under an argon atmosphere. The solution was cooled to 0°C, and *N*-hydroxysuccinimide (259 mg, 2.25 mmol) was added. The reaction mixture was allowed to warm to room temperature slowly and stirred at room temperature overnight. The reaction mixture was diluted with water and extracted with dichloromethane. The organic layer was washed with saturated brine solution, dried over anhydrous Na_2_SO_4,_ and evaporated. The crude was purified using a CombiFlash (C-18 column) with a gradient of 0–100% acetonitrile in water. The fractions with the product were combined, the acetonitrile was evaporated, and the mixture was frozen and lyophilized to give the product (750 mg, 55%) as a red-brown solid. ^1^H NMR (500 MHz, DMSO-*d*_*6*_) δ 1.05 (t, *J* = 7.2 Hz, 3H), 1.43 (s, 3H), 1.45 (s, 3H), 2.32 (s, 3H), 2.50 (s, 6H), 2.71 (s, 6H), 2.82 (brs, 4H), 2.93 (t, *J* = 6.0 Hz, 2H), 3.16–3.21 (m, 2H), 3.46–3.54 (m, 16H), 3.70 (t, *J* = 6.0 Hz, 2H), 7.07 (d, *J* = 8.1 Hz, 1H), 7.08 (s, 1H), 7.18 (d, *J* = 8.1 Hz, 1H), 8.05–8.10 (m, 2H). ^13^C NMR (125 MHz, DMSO-*d*_*6*_) δ 12.7, 12.7, 13.2, 14.7, 18.4, 25.1, 25.2, 25.4, 31.6, 33.6, 38.6, 43.6, 47.5, 65.2, 68.8, 69.4, 69.7, 69.7, 69.8, 118.6, 125.6, 126.6, 129.8, 130.0, 130.0, 130.5, 132.1, 140.5, 140.7, 144.9, 153.7, 153.8, 153.9, 163.3, 163.6, 167.3, 170.1, 172.8.

### Quantification and statistical analysis

#### Digital image analysis

Digital image analysis and quantification was done using FIJI (ImageJ)^54^ version 1.54f for Windows (https://fiji.sc/) and MetaMorph version 6.7.1 for Windows (Molecular Devices, San Jose, California USA, www.moleculardevices.com).

##### Photostability studies in J774 macrophages

Stacks of images for each acquired field were corrected for background intensity by subtracting the 5^th^ percentile intensity value for each image in the stack. To quantify fluorescence signal per field, a sum projection for each stack (sum of each image in the stack) was generated, and total integrated intensity (F) per field was measured. Fluorescence was normalized to the intensity corresponding to the first irradiation cycle (F_0_) and plotted as F/F_0_ against cycle time.

##### Cytotoxicity study in J774 macrophages

Stacks of images for each acquired field were corrected for background intensity by subtracting the 5^th^ percentile intensity value for each image in the stack. To quantify cell nuclei, a sum projection for each stack was generated, and nuclei were detected and counted using the ‘Count Nuclei’ function in Metamorph applied to the Hoechst staining channel. Approximate min and max widths for individual nuclei were set to 5 and 30 μm, respectively, and the threshold intensity above the local background was set between 50 and 200 gray levels.

##### Live ratiometric LE/Ly pH imaging of macrophage cell lines

Stacks of images for each acquired field were corrected for background intensity by subtracting the 5^th^ percentile intensity value for each image in the stack. To quantify pH per field, a sum projection for each stack was generated, and fluorescence signal from labeled compartments was selected using an intensity threshold applied to the pH-independent channel (Alexa 647 or LysoSensor yellow/blue’s pH-independent channel). A mask was then generated and transferred to the pH-dependent channel (green fluorescence). Integrated intensity was measured for each masked channel, and pH-sensitive/pH-independent ratios were calculated for each field. To quantify pH per LE/Ly, the fluorescence signal from labeled compartments was selected using an intensity threshold applied to the pH-independent channel, for each individual plane. The internally thresholded objects function in MetaMorph was used to identify individual objects corresponding to labeled compartments. Two adjacent objects would be separated when the peak intensities of their Gaussian distribution differed by at least 50%. Dissected objects were analyzed using the integrated morphometry analysis function, with *object area* filter set between 10 and 1500 calibrated units in order to discard objects smaller than LE/Lys (e.g., single pixels), and *outer radius distance* filter set between 0 and 1000 calibrated units. The filtered integrated intensity for the pH-sensitive and pH-independent channels was measured for each dissected object, and their ratio values were calculated. Ratios calculated for each field, cell or object were interpolated to pH values using ratio-to-pH calibration curves. To prepare the curves, fixed J774 macrophages loaded with derivatized dextrans were incubated in 50 mM TRIS maleate pH 5.0 buffer and imaged as described earlier. pH 5.0 fluorescence ratios were calculated per field or per object and used to generate all subsequent ratios corresponding to pH 3.5–7.4 buffers using titration data for the same dextrans measured in solution using a spectrophotometer. The resulting ratio-to-pH calibrations were fit to 4-component sigmoidal curves.

##### Color-coded ratio image generation based on ApHID-Alexa 647 ratiometric pH imaging

To generate color-coded, pixel-by-pixel ratiometric pH images, background-corrected stacks (see previous sections above) were thresholded using an intensity threshold applied to the Alexa 647 channel. A mask was then generated, which was applied to both the Alexa 647 and ApHID channels, so that only fluorescence corresponding to acidic compartments was conserved. Next, a Gaussian filter (7x7 pixels) was applied to all images. A 24-bit ratio image was generated with the filtered ApHID and Alexa 647 channels using the “ratiometric image” module in MetaMorph. ApHID/Alexa 647 ratios measured in fixed cells incubated in pH 4.5 or pH 6.0 buffers containing membrane-permeant equilibrators were assigned as minimum and maximum ratios, respectively. In the ratio images, blue hue indicates mildly acidic pH whereas tones toward green and red correspond to acidic pH.

#### Statistical data analysis

Statistical analyses were performed using GraphPad Prism version 10.3.1 for Windows (GraphPad Software, Boston, Massachusetts, USA, www.graphpad.com). Experiments in solution were repeated independently at least twice, and averages ±SEM are shown (Figures 1, 2, 4, and S3). For buffer equilibration assays in fixed cells, experiments were repeated three times; 2 wells were imaged per buffer condition and 3 fields were acquired per well (Figures S1 and S2). Cytotoxicity experiments were repeated three times; 2 to 4 wells were imaged per experiment and probe, and 3 fields were acquired per well. Averaged normalized cell counts per well ±SEM are shown (Figures 5B–5D). Differences in cell count means between conditions were assessed using the two-tailed unpaired Student’s t test. Photostability measurements in fixed cells were repeated twice; 2 dishes were imaged per probe and experiment, and 4 fields were acquired per dish. Photostability measurements in live cell culture were repeated three times; 2 wells were imaged per condition and experiment, and 3 fields were acquired per well. Averaged F/F_0_ per well ±SEM is shown (Figure 3). For ratiometric imaging of fixed or live cells, experiments were repeated 2 to 3 times; 2 to 4 wells were imaged per condition and experiment (6–12 wells in total) and 3 to 6 fields were acquired per well (Figures 5, 6, and 7). Averaged LE/Ly pH per well or per cell ±SEM, or averaged pH per LE/Ly compartment ±SD are shown. Differences between LE/Ly pH means per well between probes and conditions were assessed using the unpaired Welch and Brown-Forsythe one-way ANOVA followed by Dunnett’s multiple comparison test with 95% confidence interval (Figures 6 and 7). Forsythe’s and Bartlett’s tests were used to assess differences in the data’s standard deviations between conditions. P-values are shown as *p* > 0.05 (ns), *p* ≤ 0.05 (∗), *p* ≤ 0.01 (∗∗), *p* ≤ 0.001 (∗∗∗), and *p* ≤ 0.0001 (∗∗∗∗).

## Supplemental information

Document S1. Figures S1–S3, Tables S1–S20, Methods S1, and Data S1

Document S2. Article plus supplemental information
