# Real-time pH imaging of macrophage lysosomes using the pH-sensitive probe ApHID

**DOI:** 10.1016/j.crmeth.2025.101240

**Published:** 2025-10-24

**Authors:** Santiago Solé-Domènech, Pradeep Kumar Singh, Lucy Funes, Cheng-I J. Ma, J. David Warren, Frederick R. Maxfield

## Main text

(Cell Reports Methods *5*, 101203; October 20, 2025)

In the originally published version of this paper in Figure 7J and supporting supplemental table ST20, the authors mistakenly performed the Brown-Forsythe and Welch ANOVA tests with multiple comparison between a subset of the depicted experimental conditions. The calculations in ST20 and the annotation of the *p* values in Figure 7J now incorporate all necessary comparisons and have been updated online. The previously reported trends and individual comparisons meeting the significance threshold of 0.05 have not changed. These updated results confirm the original conclusions of the figure. The authors apologize for any inconvenience.

**Figure 7J:**
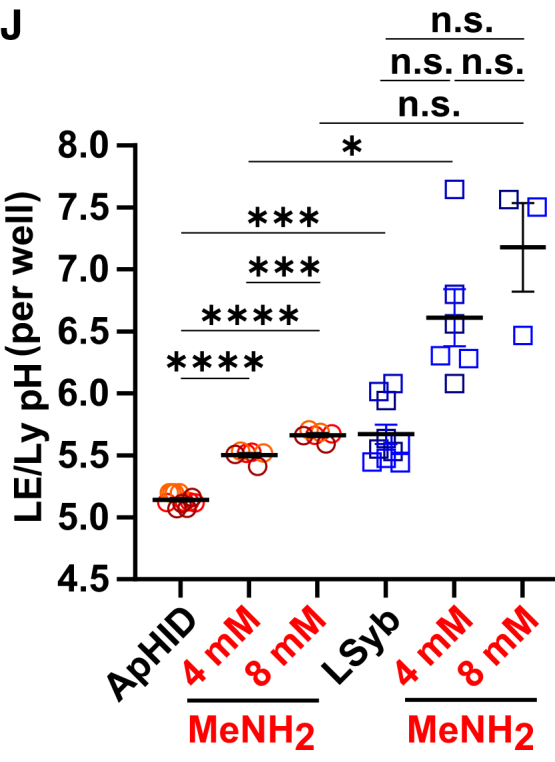
ApHID reports endolysosomal pH in stable manner over prolonged periods of time and is sensitive to subtle alkalinization in real time (corrected)

**Figure 7J:**
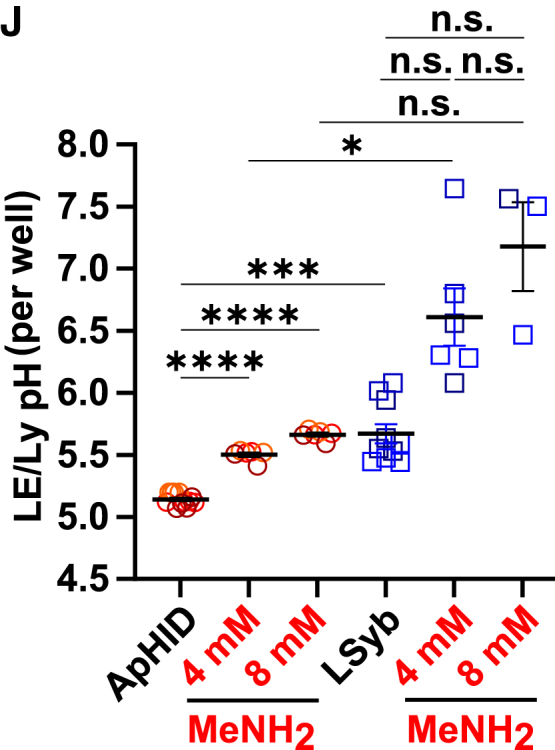
ApHID reports endolysosomal pH in stable manner over prolonged periods of time and is sensitive to subtle alkalinization in real time (original)

