# Supplementary material for: Real-time pH imaging of macrophage lysosomes using the pH-sensitive probe ApHID
Source: Cell Rep Methods. 2025 Oct 14;5(10):101203. doi: 10.1016/j.crmeth.2025.101203 (PMC12570321; doi:10.1016/j.crmeth.2025.101203)
Supplement: Document S1. Figures S1–S3, Tables S1–S20, Methods S1, and Data S1 [file mmc1.pdf]

Cell Reports Methods, Volume *5*

Supplemental information

Real-time pH imaging of macrophage lysosomes

using the pH-sensitive probe ApHID

Santiago Solé-Domènech, Pradeep Kumar Singh, Lucy Funes, Cheng-I J. Ma, J. David Warren, and Frederick R. Maxfield

SUPPLEMENTARY SECTION

SUPPLEMENTARY FIGURES

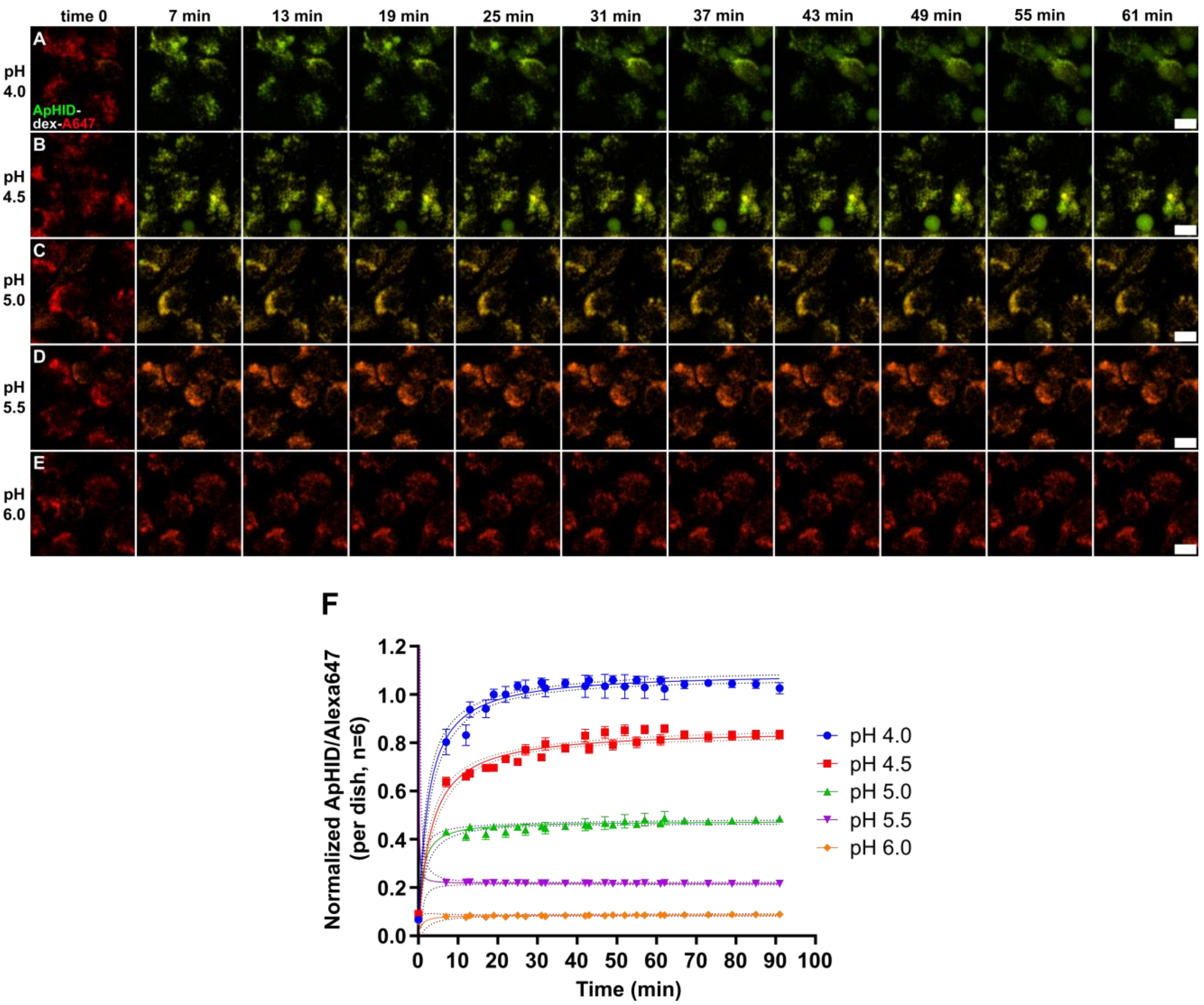

Suppl. Fig S1. Time course of pH equilibration in buffers containing membrane-permeant equilibrators in J774 macrophages at 27 ºC, related to Figs. 5-7. (A-E) J774 macrophage LE/Lys were loaded with amino-dextrans labeled with NHS-ApHID and NHS-Alexa 647 (pH-independent) and fixed with 0.5% PFA. The cells were initially imaged in 1X PBS (A-E, time 0 column) and then incubated with various pH-adjusted buffers (pH 4.0 to 6.0, for 1 h and imaged every 6 mins at 27 °C using confocal microscopy (A-E, 7 to 61 min columns). The buffers contained 40 mM methylamine, 40 mM sodium acetate 40 µM monensin as membrane-permeant pH equilibrators. Overlays showing ApHID (green) and Alexa 647 (red) channels are shown for each buffer condition. Cells in 1X PBS (pH 7.4) showed almost no ApHID signal (A-E, time 0 column). When pH-adjusted buffers were added, the ApHID signal increased over time, proportionally to buffer acidity (A-E, 7 to 61 min columns). As expected, the ApHID signal was brightest at pH 4.0. However, at that pH, some cells started to show membrane swelling and cytosolic dextran fluorescence (A). This is likely due to LE/Ly membrane damage, which caused dextran leakage. This effect was also seen, although to a lesser extent, when pH 4.5 buffer was used (B). Cells incubated in pH 5.0-6.0 buffers did not show any membrane damage or fluorescence leakage over time (C-E). **(F)** ApHID/Alexa 647 fluorescence ratios calculated for each buffer condition were normalized to pH 4.0 condition (20 min time point) and plotted against buffer incubation time. Ratio equilibration reached a stable plateau in pH 5.0-6.0 buffers within 30 mins of incubation (C-E), whereas equilibration was slower in pH 4.0 and pH 4.5 buffers (A-B). Based on these data, the use of 50 mM TRIS maleate pH 5.0 buffer seems optimal for our calibration protocol. Data were fit to a rectangular hyperbola for visualization purposes only. Two wells per buffer pH condition were imaged, and 3 fields were acquired per well. The experiment was repeated three times. Geometrical objects and bars indicate averages ± SEM. Scale bars: 10 µm. Abbreviations: ‘A647’: Alexa Fluor 647.

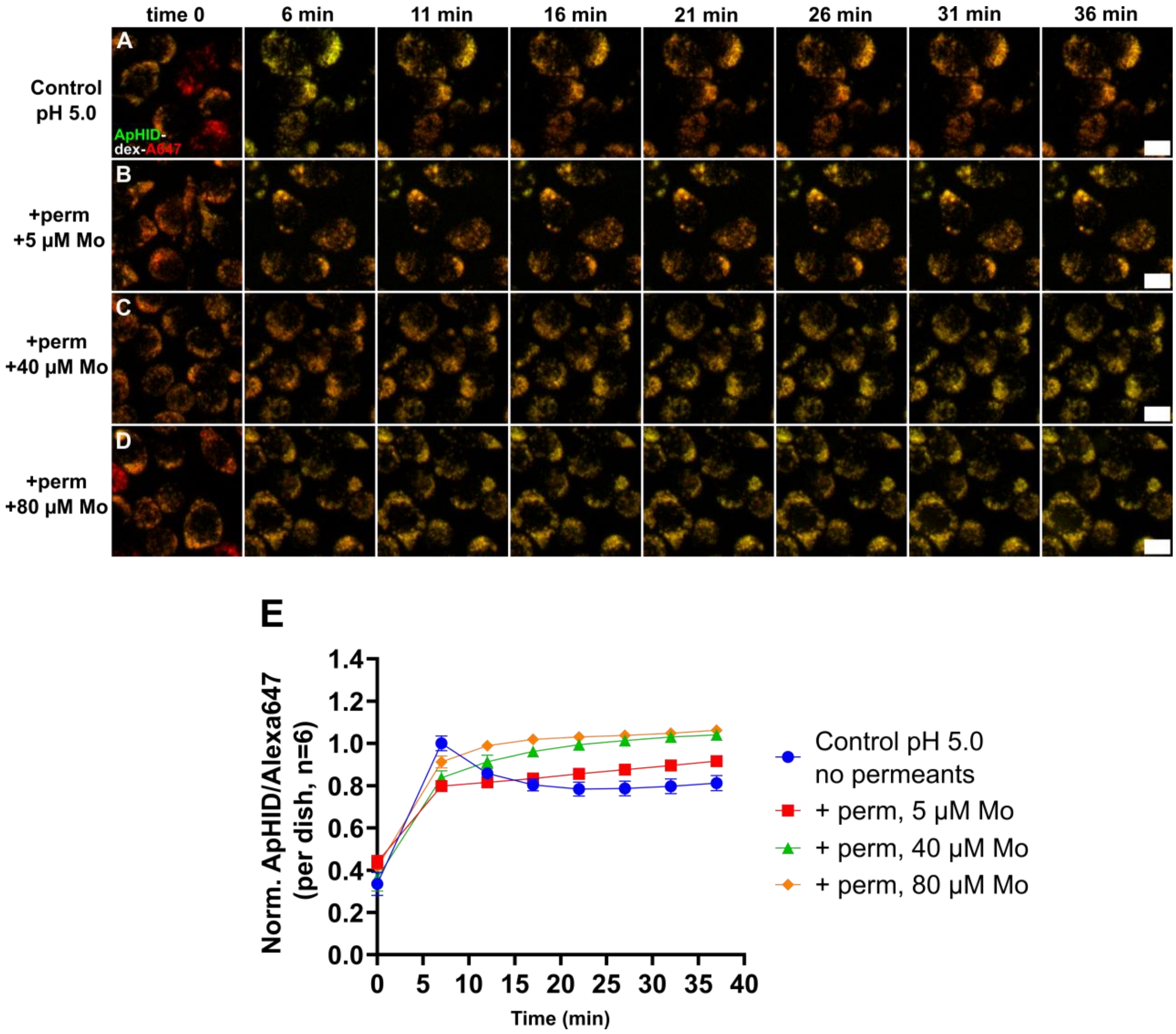

Suppl. Fig S2. Supplementation of TRIS maleate pH 5.0 buffer with monensin facilitates buffer equilibration across membranes at 37 ºC, related to Figs. 5-7. To determine the optimal concentration of monensin to add to TRIS maleate pH 5.0 buffer used in our calibrations, we incubated fixed J774 macrophages, loaded with ApHID-Alexa 647 dextrans, with the buffer (see earlier section) supplemented with various concentrations of monensin, and measured ApHID/Alexa 647 fluorescence ratio at 37 ºC over time. **(A-D)** Cells were imaged in 1X PBS (A-D, time 0 column) and then in 50 mM TRIS maleate pH 5.0 buffer alone (A) or supplemented with 40 mM methylamine, 40 mM sodium acetate and 5 µM (B), 40 µM (C), or 80 µM (D) monensin (time 6 min to 36 min columns). Cells were imaged every 5 min for 40 min. Overlays show ApHID (green) and Alexa 647 (red) channels for each buffer condition and timepoint. Monensin supplementation led to increased ApHID fluorescence relative to cells in buffer without membrane permeants (B-D vs A). **(E)** ApHID/Alexa 647 fluorescence ratios measured for each buffer condition were normalized to 80 µM monensin condition (36 min time point) and plotted against incubation time. 40 µM and 80 µM monensin addition led to ApHID/Alexa 647 ratio stabilization within 30 mins of incubation (C-D), whereas the absence of membrane permeants altogether led to incomplete ratio equilibration (A). According to these data, the supplementation of the TRIS maleate pH 5.0 buffer with 40 or 80 µM monensin, together with 40 mM sodium acetate and 40 mM methylamine hydrochloride, ensured optimal buffer equilibration across membranes in fixed J774 macrophages at 37 °C. The experiment was repeated three times. Two wells were imaged per condition, and 3 fields acquired per well. Geometrical objects and bars indicate averages ± SEM. Scale bars: 10 µm. Abbreviations: 'perm': membrane-permeant equilibrators. 'A647': Alexa Fluor 647.

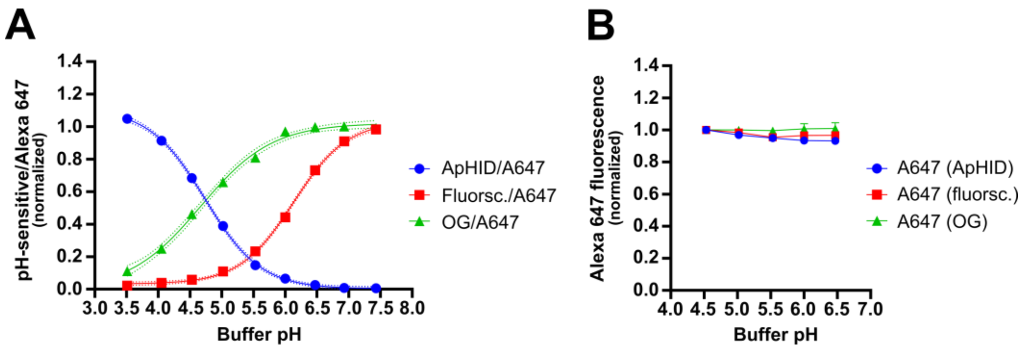

Supplementary Figure S3. Averaged pH-sensitive/Alexa 647 vs. buffer pH titrations used to prepare ratio-to-pH calibrations for ratiometric pH imaging of J774 macrophages, related to Figs. 5 to 7. (A) pH-sensitive/Alexa 647 fluorescence ratios vs. buffer pH titrations for 70 KDa amino-dextrans labeled with either NHS-probe pH sensor and NHS-Alexa 647 (pH-independent). To prepare each individual curve, dextrans were solubilized in buffers with pH adjusted between 3.5 and 7.5 containing 10% FBS, 40 mM methylamine hydrochloride, 40 mM sodium acetate and 40 µM monensin, and measured at 37 °C using a spectrophotometer (see STAR Methods section). The resulting ratio-to-pH titrations were fit to 4-component sigmoidal curves (superimposed colored curves). Titration data in solution were used to prepare full calibration curves for fluorescence ratio-to-pH interpolation (See Figs. 5-7, Suppl. Figs. S1-S2 and STAR Methods Section). **(B)** Alexa 647 fluorescence plotted against buffer pH for each dextran is generally pH-independent in the pH range of 4.5-6.5. Dextran solutions were loaded into 384-well plates in triplicate. See Suppl. Table ST4 for statistics. Measurements in solution were repeated twice. Geometric figures and bars represent averaged ratios ± SEM (most error bars fit within the symbols). Superimposed black dotted lines represent the standard error of the sigmoidal fit and are superimposed on the curves.

SUPPLEMENTARY TABLES ST1-ST3

Supplementary Table ST1 related to Figure 2. Most Common commercially available pH sensors.

pH sensor	Vendor	pKa	Ex/Em max	Brighter with acidity?	Structure	Accumulates in LE/Lys?
**ApHID**	**WCM**	**5**	**502/515 nm**	**yes**	**Weak acid**	**Yes (dextran)**
**Oregon Green**	Various	4.7	500/525 nm	no	Xanthene	Yes (dextran)
**pHlys™ Green**	Dojindo	unknown	440/539 nm	yes	Weak base?	Within 30 minutes
**LysoSensor™ y/b**	Thermo Fisher	3.6	381/521 nm	yes	Weak base	Yes (dextran)
**Fluorescein**	Various	6.4	495/520 nm	no	Xanthene	Yes (dextran)
**pHrodo™ green**	Thermo Fisher	6.5	509/533 nm	no	Undisclosed	Yes (dextran)
**Protonex™ Green**	AAT Bioquest	6.5	445/503 nm	yes	Weak base?	Within 15-120 minutes
**Magic™**	Creative	6.5	509/533 nm	yes	Undisclosed	Yes (dextran)
**BioTracker™ Orange**	Millipore Sigma	5.3, 5.88	544/565 nm	yes	Carbocyanine	Yes (dextran)
**pHrodo™ Deep red**	Thermo Fisher	5.2	640/655 nm	yes	Undisclosed	Yes (dextran)
**pHrodo™ red**	Thermo Fisher	6.5	560/587 nm	no	Undisclosed	Yes (dextran)
**CypHer5E**	Cytiva	7.3	647/667 nm	no	Cyanine	Yes (dextran)
**pHlys™ Red**	Dojindo	~4	562/586 nm	yes	Weak base?	Within 30 minutes

Supplementary Table ST1. Most common commercially available pH sensors and their properties, related to Figure 2. Vendor, pKa, excitation and emission max. are listed, together with their fluorescence behavior with acidity, structure (if disclosed) and whether they readily accumulate in endolysosomal compartments. Probe information was retrieved from each respective vendor's webpage.

Suppl. Table ST2 related to Figure 2. pKa values for Green-emitting probes.

	ApHID	Oregon Green	Fluorescein
**pH-adjusted buffers**	5.02 ± 0.02	4.74 ± 0.06	6.11 ± 0.01
**Buffers + 50 mg/mL BSA**	4.87 ± 0.01	4.41 ± 0.15	6.15 ± 0.04
**Buffers + 1 mM $MgCl_2$, $CaCl_2$**	5.02 ± 0.02	4.79 ± 0.02	6.17 ± 0.06
**Buffers + Chloride → Acetate**	4.94 ± 0.04	4.82 ± 0.03	6.26 ± 0.08
**Buffers + 0.2 mM Fe(II)**	5.32 ± 0.08	5.15 ± 0.01	6.38 ± 0.02
**Buffers + 0.1 mM •OH**	5.36 ± 0.08	5.14 ± 0.02	6.38 ± 0.06
**Buffers + 0.2 mM •OH**	5.39 ± 0.09	5.12 ± 0.06	6.34 ± 0.07

Supplementary Table ST2. Log IC_{50} (pKa) values ± SEM calculated from sigmoidal curves fit to titrations shown in Figs. 2D-2G, related to Fig 2. Buffers with pH adjusted between 1.5 and 8.5 (see STAR Methods section) alone or enriched in either BSA, $CaCl_2$ + $MgCl_2$, or sodium acetate (as a substitute for sodium chloride) were tested on fluorescent probes attached to amino-dextrans. Oxidation by •OH was tested on the hydrolyzed succinimidyl ester form of the probes. Note that the pKa of the probes is generally lower when attached to dextrans. This has been reported previously for fluorescein [S1]. The experiments were repeated twice.

Supplementary Table ST3 related to Figures. 1-7. Amino-dextran labeling and probe incorporation.

Dex. Weight (KDa)	*NHS ester excess molar ratio (reacted vs. incorporated)*							
**Labeling conc. mg/mL**	**Vendor**	**Experiment**	**x ApHID**	**x fluorescein**	**x OG**	**x Alexa 405**	**x Cy5-3xSO_3**	**x A647**
10 / 50	TS	*General spectroscopy*	2.35 (1.6)	1.43 (1.2)	1.43 (1.2)	-	-	
70 / 20	Fina	*Derivatization*	2 (1), 4 (2), 6 (3), 8 (4)	-	-	4 (2)	4 (1.4)	
70 / 20	Fina	*Net charge effect*	3 (1.5)	-	-	3 (1.3), 15 (6), 100 (22)	-	
70 / 25	TS	*Photobleaching*	3 (2.1)	3 (1.8)	3 (1.9)	-	-	
70 / 25	TS	*Ratiometric pH imaging*	3 (1.6)	3 (1.8)	-	3 (1.8)	-	
			4 (2.6)	4 (1.69)	4 (2.06)	-	4-6 (2.8-3)	3 (1.8-2.2)

Supplementary Table ST3. Dextrans used for each experiment in the study, related to Figs. 1-7. Information for each dextran is shown, including weight (KDa), concentration during labeling with probes (mg/mL), vendor, experimental use, identity of the probe attached, dextran:NHS probe molar ratio used for labeling (black characters) and resulting probe incorporation after dialysis (red characters). Abbreviations: TS (Thermo Fisher); Fina (Fina Biosolutions), KDa (kiloDalton).

SUPPLEMENTARY TABLES ST4-ST20

Supplementary Table ST4 related to Suppl. Figure S3.
Green-emitting pH-sensitive probe/Alexa 647 fluorescence ratios vs. buffer pH calibrations in presence of FBS, measured at 37 °C. 70 KDa amino-dextrans. Descriptive statistics.

	**ApHID-A647 dex +FBS @37 °C**			**Fluorsc-A647 dex +FBS @37 °C**			**OG-A647 dex +FBS @37 °C**		
**pH buffer**	Norm. ApHID/A647	± SEM	n	Norm. Fluorsc./A647	± SEM	n	Norm. OG/A647	± SEM	n
**3.51**	1.05	0.02	2	0.02	0.00	2	0.11	0.00	2
**4.05**	0.91	0.01	2	0.04	0.00	2	0.25	0.00	2
**4.53**	0.68	0.01	2	0.06	0.00	2	0.46	0.01	2
**5.02**	0.39	0.00	2	0.11	0.01	2	0.66	0.00	2
**5.53**	0.15	0.01	2	0.23	0.01	2	0.81	0.01	2
**6**	0.06	0.00	2	0.44	0.00	2	0.97	0.01	2
**6.47**	0.03	0.00	2	0.73	0.00	2	1.00	0.01	2
**6.93**	0.01	0.00	2	0.91	0.01	2	1.00	0.00	2
**7.43**	0.01	0.00	2	0.98	0.01	2	0.99	0.00	2

	**ApHID-A647 dex +FBS @37 °C**			**Fluorsc-A647 dex +FBS @37 °C**			**OG-A647 dex +FBS @37 °C**		
**pH buffer**	Norm. Alexa 647 fluo.	± SEM	n	Norm. Alexa 647 fluo.	± SEM	n	Norm. Alexa 647 fluo.	± SEM	n
**3.51**	1.05	0.00	2	1.06	0.03	2	1.02	0.01	2
**4.05**	1.03	0.02	2	1.02	0.02	2	1.02	0.01	2
**4.53**	1.00	0.00	2	1.00	0.00	2	1.00	0.00	2
**5.02**	0.97	0.00	2	0.98	0.02	2	1.00	0.01	2
**5.53**	0.95	0.02	2	0.95	0.02	2	1.00	0.02	2
**6**	0.93	0.00	2	0.97	0.02	2	1.01	0.03	2
**6.47**	0.93	0.01	2	0.97	0.02	2	1.01	0.04	2
**6.93**	0.92	0.00	2	0.96	0.01	2	0.97	0.01	2
**7.43**	0.91	0.00	2	0.99	0.01	2	0.98	0.02	2

Supplementary Table ST4. Normalized average pH-sensitive/Alexa 647 ratios and Alexa 647 fluorescence intensity vs. buffer pH titrations measured for dextrans in solution, related to Suppl. Figure S3. Ratio titrations for each probe (from two measurements in solution) presented here were used to interpolate fluorescence ratios to pH for experiments using J774 macrophages (Figure 6), and similar calibrations were used to interpolate pH for Figures 5 and 7 (not shown). Average Alexa 647 intensity is generally pH-independent. Abbreviations: fluorsc, fluorescein; OG, Oregon Green; A647, Alexa 647.

Supplementary Table ST5 related to Figure 1A
Descriptive statistics

ApHID φf vs buffer pH titration (n=2)

Buffer pH	ApHID φf	± SEM	n
3.96	0.52	0.002	2
4.47	0.47	0.02	2
4.96	0.42	0.02	2
5.51	0.25	0.02	2
5.99	0.13	0.01	2

ApHID φf at pH 3.0 (n=2)

Buffer pH	ApHID φf	± SEM	n
3.00	0.642	0.046	2

ApHID ε at pH 3.0 (n=2)

Buffer pH	ApHID ε	± SEM	n
3.00	99710	23.5	2

Supplementary Section Table ST5. Quantum yield and extinction coefficient for ApHID (hydrolyzed NHS ester form), measured in solution, **related to Figure 1A.** Quantum yield was measured in several pH-adjusted buffers using fluorescein as a reference at in 0.01 M NaOH (see STAR Methods section). The measurements were repeated twice. Average quantum yield or extinction coefficient ± SEM are presented. Abbreviations: φf: quantum yield; ε: extinction coefficient.

Supplementary Table ST6 related to Figure 2A. Absorbance vs buffer pH. 10 KDa amino-dextrans. Descriptive statistics.												
	ApHID			Fluorescein			Oregon Green			LysoSensor™ y/b		
**Buffer pH**	Norm. Abs.	± SEM	n	Norm. Abs.	± SEM	n	Norm. Abs.	± SEM	n	Norm. Abs.	± SEM	n
**1.5**										0.96	0.044	2
**2**							0.12	0.020	2	0.98	0.019	2
**2.5**	1.00	0.000	2				0.08	0.004	2	0.88	0.035	2
**3**	0.92	0.022	2				0.14	0.011	2	0.79	0.067	2
**3.5**	0.92	0.002	2	0.11	0.004	2	0.15	0.023	2	0.67	0.057	2
**4**	0.80	0.011	2	0.18	0.020	2	0.32	0.008	2	0.47	0.060	2
**4.5**	0.77	0.040	2	0.23	0.008	2	0.52	0.025	2	0.35	0.048	2
**5**	0.75	0.025	2	0.26	0.008	2	0.76	0.013	2	0.29	0.006	2
**5.5**	0.75	0.039	2	0.35	0.012	2	0.92	0.011	2	0.25	0.005	2
**6**	0.72	0.039	2	0.52	0.019	2	0.97	0.011	2	0.25	0.015	2
**6.5**	0.68	0.016	2	0.70	0.006	2	1.00	0.000	2	0.18	0.049	2
**7**	0.71	0.001	2	0.87	0.004	2	0.96	0.017	2			
**7.5**				0.95	0.049	2						
**8**				0.95	0.052	2						
**8.5**				0.96	0.003	2						

Supplementary Table ST6. Absorbance vs pH of green-emitting pH-sensitive probes, related to Figure 2A. Absorbance was measured for ApHID, fluorescein, Oregon Green and LysoSensor™ yellow/blue (attached to 10 KDa amino-dextrans) in solution using a spectrophotometer, plotted against buffer pH. Averaged normalized absorbance ± SEM is shown. The experiment was repeated twice.

Suppl. Table ST7 related to Figure 2B. Fluorescence vs buffer pH. 10 KDa amino-dextrans. Descriptive statistics.									
	ApHID			Fluorescein			Oregon Green		
**Buffer pH**	Norm. Fluo.	± SEM	n	Norm. Fluo.	± SEM	n	Norm. Fluo.	± SEM	n
**2**				0.01	0.001	2	0.02	0.000	2
**2.5**	1.00	0.000	2	0.01	0.000	2	0.02	0.000	2
**3**	0.99	0.004	2	0.02	0.000	2	0.04	0.003	2
**3.5**	0.93	0.015	2	0.04	0.002	2	0.09	0.017	2
**4**	0.81	0.005	2	0.07	0.004	2	0.22	0.015	2
**4.5**	0.67	0.037	2	0.10	0.001	2	0.40	0.006	2
**5**	0.39	0.028	2	0.14	0.004	2	0.58	0.006	2
**5.5**	0.18	0.000	2	0.27	0.014	2	0.77	0.025	2
**6**	0.06	0.009	2	0.47	0.013	2	0.93	0.082	2
**6.5**	0.02	0.001	2	0.71	0.006	2	0.91	0.015	2
**7**	0.01	0.000	2	0.82	0.047	2	0.97	0.043	2
**7.5**				0.93	0.003	2	0.97	0.039	2
**8**				1.00	0.000	1			
**8.5**				0.99	0.018	2			

Fluorescence ratio vs. buffer pH – sigmoidal fit and pKa			
	**ApHID**	**Fluorescein**	**Oregon Green**
**log IC_{50} (pKa)**	4.79	6.10	4.74
**R^2**	0.9981	0.9978	0.9947

Supplementary Table ST7. Fluorescence vs pH for green-emitting pH-sensitive probes, related to Figure 2B. Fluorescence was measured for ApHID, fluorescein, Oregon Green and LysoSensor™ yellow/blue (attached to 10 KDa amino-dextrans) in solution using a spectrophotometer. Averaged normalized fluorescence ± SEM is shown. Fluorescence was plotted against buffer pH (Fig. 2B) and yielded titrations that were fitted to 4-component sigmoidal curves, from which log IC_{50} was calculated for each probe corresponding to their pKa. The adjusted R-squared of the sigmoidal fit (R^2) is shown for each calculated pKa. The experiment was repeated twice.

Supplementary Table ST8 related to Figure 2C. Fluorescence vs. buffer pH for green, orange and red-emitting pH-sensitive probes – 70 KDa dextrans. Descriptive statistics.

	ApHID			BioTracker™ Orange			pHrodo™ Deep Red			LysoSensor™ y/b		
**Buffer pH**	Norm. Fluo.	± SEM	n	Norm. Fluo.	± SEM	n	Norm. Fluo.	± SEM	n	Norm. Fluo.	± SEM	n
**2**										1.00		2
**2.5**										0.98	0.005	2
**3**	1.00		2	1.00		2				0.89	0.016	2
**3.5**	0.96	0.001	2	0.96	0.012	2				0.70	0.003	2
**4**	0.82	0.005	2	0.94	0.012	2	0.93	0.016	2	0.40	0.013	2
**4.5**	0.65	0.017	2	0.87	0.002	2	1.00	0.000	2	0.22	0.006	2
**5**	0.41	0.024	2	0.79	0.038	2	0.73	0.048	2	0.11	0.002	2
**5.5**	0.17	0.008	2	0.67	0.007	2	0.50	0.047	2	0.06	0.000	2
**6**	0.07	0.003	2	0.41	0.004	2	0.19	0.001	2	0.05	0.000	2
**6.5**	0.02	0.002	2	0.23	0.006	2	0.05	0.007	2	0.05	0.002	2
**7**	0.01	0.004	2	0.09	0.001	2	0.04	0.019	2	0.05	0.004	2
**7.5**				0.03	0.001	2						
**8**				0.01	0.002	2						

Fluorescence ratio vs buffer pH - sigmoidal fit and pKa

	ApHID	BioTracker™ Orange	pHrodo™ Deep Red	LysoSensor™ y/b
**log IC_{50} (pKa)**	4.77	5.88	5.47	3.79
**R^2**	0.998	0.996	0.981	0.999

Supplementary Table ST8. Fluorescence vs pH for green, orange and red-emitting pH-sensitive probes, related to Figure 2C. Fluorescence was measured for ApHID, BioTracker™ Orange, pHrodo™ Deep Red (attached to 70 KDa amino-dextrans) and LysoSensor™ yellow/blue (attached to 10 KDa dextrans) in solution using a spectrophotometer. Averaged normalized fluorescence ± SEM is shown. Fluorescence was plotted against buffer pH (Fig. 2C) and yielded titrations that were fitted to 4-component sigmoidal curves, from which log IC_{50} was calculated for each probe corresponding to their pKa. The adjusted R-squared of the sigmoidal fit (R^2) is shown for each calculated pKa. The experiment was repeated twice.

Suppl. Table ST9 related to Figure 2B-2C. Dynamic range in the pH 4.0-6.0 window, renormalized to pH 6.0, for all probes (10 or 70 KDa dextrans). Descriptive statistics.

Green probes — $F/F_{(pH\ 6.0)}$

**Buffer pH**	ApHID	Fluorescein	Oregon Green
**4**	12.80	0.14	0.23
**4.5**	10.62	0.21	0.44
**5**	6.16	0.29	0.63
**5.5**	2.91	0.57	0.83
**6**	1.00	1.00	1.00

Green – orange – red probes — $F/F_{(pH\ 6.0)}$

**Buffer pH**	ApHID	BioTracker™ Orange	pHrodo™ Deep Red	LysoSensor™ y/b
**4**	12.25	2.26	5.01	7.29
**4.5**	9.59	2.10	5.36	4.05
**5**	6.12	1.91	3.93	1.97
**5.5**	2.53	1.62	2.69	1.16
**6**	1.00	1.00	1.00	1.00

Supplementary Table ST9. Fluorescence dynamic range for all pH-sensitive probes renormalized to pH 6.0, related to Figure 2B-2C. Fluorescence measured in the pH 4.0-6.0 window for green, orange and red-emitting probes attached to 10 or 70 KDa amino-dextrans using a spectrophotometer, plotted against buffer pH (Suppl. Tables ST7 and ST8) and later renormalized to pH 6.0 for each corresponding probe. F/F(pH 6.0) vs buffer pH is shown.

Suppl. Table ST10 related to Figure 2D. Effect of ROS on probe fluorescence and pKa. Hydrolyzed NHS ester form of the probes. Descriptive statistics.

	ApHID + 0.2 mM Fe(II)			ApHID + 0.1 mM Fe(II) + 1 mM H_2O_2			ApHID + 0.2 mM Fe(II) + 1 mM H_2O_2		
**Buffer pH**	Norm. Fluo	± SEM	n (exp)	Norm. Fluo	± SEM	n (exp)	Norm. Fluo	± SEM	n (exp)
**4**	1.00	-	2	0.92	0.03	2	0.93	0.03	2
**4.5**	0.94	0.02	2	0.85	0.01	2	0.86	0.02	2
**5**	0.74	0.04	2	0.69	0.01	2	0.71	0.02	2
**5.5**	0.46	0.02	2	0.43	0.02	2	0.46	0.01	2
**6**	0.19	0.01	2	0.21	0.00	2	0.22	0.02	2
**6.5**	0.06	0.00	2	0.11	0.00	2	0.12	0.01	2

	Fluorescein + 0.2 mM Fe(II)			Fluorescein + 0.1 mM Fe(II) + 1 mM H_2O_2			Fluorescein + 0.2 mM Fe(II) + 1 mM H_2O_2		
**Buffer pH**	Norm. Fluo	± SEM	n (exp)	Norm. Fluo	± SEM	n (exp)	Norm. Fluo	± SEM	n (exp)
**5**	0.14	0.03	2	0.13	0.03	2	0.09	0.03	2
**5.5**	0.18	0.04	2	0.17	0.03	2	0.14	0.03	2
**6**	0.33	0.05	2	0.37	0.06	2	0.33	0.03	2
**6.5**	0.67	0.00	2	0.68	0.00	2	0.66	0.00	2
**7**	0.85	0.00	2	0.92	0.01	2	0.97	0.03	2
**7.5**	1.00	-	2	1.10	0.01	2	1.05	0.02	2

	OG + 0.2 mM Fe(II)			OG + 0.1 mM Fe(II) + 1 mM H_2O_2			OG + 0.2 mM Fe(II) + 1 mM H_2O_2		
**Buffer pH**	Norm. Fluo	± SEM	n (exp)	Norm. Fluo	± SEM	n (exp)	Norm. Fluo	± SEM	n (exp)
**3.5**	0.09	0.02	2	0.08	0.01	2	0.07	0.02	2
**4**	0.17	0.02	2	0.15	0.01	2	0.12	0.02	2
**4.5**	0.33	0.00	2	0.29	0.00	2	0.28	0.01	2
**5**	0.49	0.02	2	0.48	0.02	2	0.49	0.00	2
**5.5**	0.67	0.01	2	0.71	0.04	2	0.69	0.03	2
**6**	0.91	0.00	2	0.92	0.03	2	0.94	0.06	2
**6.5**	1.00	-	2	0.98	0.03	2	0.98	0.01	2

Fluorescence ratio vs buffer pH - sigmoidal fit and pKa

	ApHID		
	**0.2 mM Fe(II)**	**0.1 mM Fe(II) + 1 mM H_2O_2**	**0.2 mM Fe(II) + 1 mM H_2O_2**
**log IC_{50} - pKa (R^2)**	5.40 (0.994)	5.38 (0.996)	5.41 (0.993)
	**Fluorescein**		
	**0.2 mM Fe(II)**	**0.1 mM Fe(II) + 1 mM H_2O_2**	**0.2 mM Fe(II) + 1 mM H_2O_2**
**log IC_{50} - pKa (R^2)**	6.39 (0.985)	6.45 (0.987)	6.38 (0.993)
	**Oregon Green**		
	**0.2 mM Fe(II)**	**0.1 mM Fe(II) + 1 mM H_2O_2**	**0.2 mM Fe(II) + 1 mM H_2O_2**
**log IC_{50} - pKa (R^2)**	5.24 (0.994)	5.14 (0.991)	5.11 (0.987)

Supplementary Table ST10. Effect of reactive oxygen species on the fluorescence and pKa of green-emitting pH sensors, related to Figure 2D. Fluorescence measured for ApHID, fluorescein and Oregon Green (hydrolyzed NHS esters) in solution, exposed to various amounts of reactive oxygen species (ROS) for 24h at 37 °C. ROS were generated in mixtures containing Fe (II) and H_2O_2. After incubation, fluorescence was measured using a spectrophotometer, and fluorescence was plotted against buffer pH (Fig. 2D), yielding titrations that were fitted to 4-component sigmoidal curves, from which log IC_{50} was calculated for each probe corresponding to their pKa. The adjusted R-squared of the sigmoidal fit (R^2) is shown for each calculated pKa. The experiment was repeated twice.

Supplementary Table ST11 related to Figure 2E-2G
Effect of salts on green probe fluorescence and pKa - 10 KDa amino-dextrans. Descriptive statistics.

	ApHID in buffers			ApHID +50 mg/mL BSA			ApHID +1 mM $MgCl_2$, $CaCl_2$			ApHID, chloride → acetate		
**Buffer pH**	Norm. Fluo.	± SEM	n	Norm. Fluo.	± SEM	n	Norm. Fluo.	± SEM	n	Norm. Fluo.	± SEM	n
**4**	1.00	0.00	2	0.84	0.00	2	1.03	0.01	2	0.96	0.01	2
**4.5**	0.84	0.00	2	0.68	0.00	2	0.87	0.00	2	0.78	0.02	2
**5**	0.55	0.01	2	0.37	0.00	2	0.57	0.00	2	0.51	0.01	2
**5.5**	0.25	0.01	2	0.15	0.02	2	0.27	0.00	2	0.25	0.00	2
**6**	0.10	0.00	2	0.04	0.01	2	0.10	0.00	2	0.10	0.00	2
**6.5**	0.03	0.00	2	0.02	0.00	2	0.03	0.00	2	0.03	0.00	2

	Fluorsc. in buffers			Fluorsc. +50 mg/mL BSA			Fluorsc. +1 mM $MgCl_2$, $CaCl_2$			Fluorsc., chloride → acetate		
**Buffer pH**	Norm. Fluo.	± SEM	n	Norm. Fluo.	± SEM	n	Norm. Fluo.	± SEM	n	Norm. Fluo.	± SEM	n
**5**	0.15	0.00	2	0.16	0.01	2	0.15	0.00	2	0.15	0.00	2
**5.5**	0.28	0.00	2	0.24	0.02	2	0.26	0.00	2	0.25	0.01	2
**6**	0.54	0.00	2	0.46	0.01	2	0.52	0.00	2	0.48	0.01	2
**6.5**	0.81	0.01	2	0.70	0.02	2	0.76	0.04	2	0.74	0.00	2
**7**	1.00	0.00	2	0.86	0.02	2	0.96	0.02	2	0.95	0.03	2

	OG in buffers			OG +50 mg/mL BSA			OG +1 mM $MgCl_2$, $CaCl_2$			OG, chloride → acetate		
**Buffer pH**	Norm. Fluo.	± SEM	n	Norm. Fluo.	± SEM	n	Norm. Fluo.	± SEM	n	Norm. Fluo.	± SEM	n
**2.5**	0.02	0.00	2	0.03	0.00	2	0.02	0.00	2	0.02	0.00	2
**3**	0.04	0.00	2	0.08	0.00	2	0.04	0.00	2	0.05	0.00	2
**3.5**	0.09	0.01	2	0.15	0.00	2	0.11	0.00	2	0.11	0.00	2
**4**	0.22	0.00	2	0.27	0.04	2	0.19	0.02	2	0.21	0.00	2
**4.5**	0.43	0.02	2	0.47	0.02	2	0.42	0.00	2	0.42	0.00	2
**5**	0.65	0.02	2	0.70	0.03	2	0.64	0.01	2	0.61	0.01	2
**5.5**	0.81	0.02	2	0.78	0.06	2	0.81	0.01	2	0.79	0.03	2
**6**	0.98	0.02	2	0.84	0.11	2	0.99	0.00	2	0.96	0.01	2
**6.5**	1.00	0.00	2	0.86	0.09	2	1.01	0.01	2	1.00	0.04	2

Fluorescence ratio vs buffer pH - sigmoidal fit and pKa				
	**ApHID**			
	**Buffer**	**+50 mg/mL BSA**	**+1 mM $MgCl_2$, $CaCl_2$**	**chloride → acetate**
**log IC_{50} - pKa (R^2)**	5.01 (1.00)	4.86 (0.998)	5.01 (1.00)	4.94 (0.999)
	**Fluorescein**			
	**Buffer**	**+50 mg/mL BSA**	**+1 mM $MgCl_2$, $CaCl_2$**	**chloride → acetate**
**log IC_{50} - pKa (R^2)**	6.11 (1.00)	6.14 (0.995)	6.14 (0.992)	6.24 (0.995)
	**Oregon Green**			
	**Buffer**	**+50 mg/mL BSA**	**+1 mM $MgCl_2$, $CaCl_2$**	**chloride → acetate**
**log IC_{50} - pKa (R^2)**	4.73 (0.997)	4.42 (0.965)	4.78 (0.997)	4.81 (0.996)

Supplementary Table ST11. Effect of salts on green-emitting probe fluorescence and pKa, related to Figure 2E-2G. Fluorescence measured for ApHID, fluorescein and Oregon Green (attached to 10 KDa dextrans) in pH-adjusted buffers containing 50 mg/mL bovine serum albumin (BSA), 1 mM $MgCl_2$ and $CaCl_2$ salts or in solutions for which all chloride salts had been replaced by acetate. The probes were incubated in each respective condition for 20 h at 37 °C. After incubation, fluorescence was measured using a spectrophotometer and plotted against buffer pH (Fig. 2D), yielding titrations that were fitted to 4-component sigmoidal curves, from which log IC_{50} was calculated for each probe corresponding to their pKa. The adjusted R-squared of the sigmoidal fit (R^2) is shown for each calculated pKa. The experiment was repeated twice.

Supplementary Table ST12 related to Figure 3. Photostability of ApHID, fluorescein, Oregon Green and LysoSensor™ yellow/blue in fixed or live cells. 70 KDa amino-dextrans. Descriptive statistics.

Cycle	*Fixed J774 macrophages at 37 °C*						*Live J774 macrophages at 37 °C and 5% CO_2*				n (wells)
	**ApHID**		**Fluorescein**		**Oregon Green**		**ApHID**		**LysoSensor™ y/b**		
	Norm. F_0/F	± SEM	Norm. F_0/F	± SEM	Norm. F_0/F	± SEM	Norm. F_0/F	± SEM	Norm. F_0/F	± SEM	
1	1.00	-	1.00	-	1.00	-	1.00	-	1.00	-	4
2	1.00	0.001	0.90	0.011	0.91	0.007	1.00	0.010	1.00	0.009	4
3	1.00	0.002	0.83	0.017	0.83	0.012	1.01	0.011	1.00	0.007	4
4	0.99	0.003	0.76	0.021	0.77	0.015	1.01	0.013	1.00	0.007	4
5	0.99	0.004	0.71	0.023	0.71	0.017	1.02	0.010	1.00	0.013	4
6	0.99	0.004	0.66	0.024	0.67	0.018	1.03	0.011	1.00	0.018	4
7	0.99	0.005	0.62	0.025	0.63	0.019	1.04	0.009	1.01	0.016	4
8	0.98	0.005	0.59	0.025	0.60	0.020	1.04	0.007	1.01	0.013	4
9	0.98	0.006	0.56	0.026	0.56	0.021	1.04	0.005	1.00	0.008	4
10	0.98	0.006	0.53	0.025	0.54	0.021	1.05	0.006	1.01	0.009	4
11	0.98	0.007	0.50	0.025	0.51	0.021	1.05	0.007	1.00	0.012	4
12	0.97	0.007	0.48	0.025	0.49	0.021	1.05	0.008	1.00	0.008	4
13	0.97	0.007	0.46	0.025	0.47	0.021	1.05	0.009	1.00	0.005	4
14	0.97	0.007	0.44	0.024	0.45	0.021	1.05	0.011	1.00	0.009	4
15	0.97	0.008	0.42	0.024	0.43	0.021	1.04	0.011	1.00	0.012	4
16	0.96	0.008	0.41	0.023	0.42	0.021	1.04	0.008	1.00	0.016	4
17	0.96	0.008	0.39	0.023	0.40	0.021	1.04	0.012	1.00	0.017	4
18	0.96	0.009	0.38	0.022	0.39	0.020	1.04	0.012	1.01	0.021	4
19	0.96	0.009	0.36	0.022	0.37	0.020	1.04	0.011	1.00	0.022	4
20	0.95	0.009	0.35	0.022	0.36	0.020	1.04	0.008	0.99	0.018	4
21	0.95	0.009	0.34	0.021	0.35	0.020	1.04	0.008	1.00	0.017	4
22	0.95	0.009	0.33	0.021	0.34	0.020	1.04	0.009	1.00	0.018	4
23	0.94	0.009	0.32	0.020	0.33	0.019	1.05	0.010	1.00	0.014	4
24	0.94	0.009	0.31	0.020	0.32	0.019	1.04	0.011	1.00	0.019	4
25	0.94	0.009	0.30	0.020	0.31	0.019	1.04	0.011	1.01	0.016	4
26	0.94	0.009	0.29	0.019	0.30	0.019	1.04	0.011	1.01	0.018	4
27	0.93	0.010	0.28	0.019	0.29	0.019	1.04	0.012	1.01	0.022	4
28	0.93	0.010	0.27	0.018	0.29	0.018	1.04	0.010	1.01	0.016	4
29	0.93	0.009	0.27	0.018	0.28	0.018	1.04	0.009	1.01	0.015	4
30	0.92	0.010	0.26	0.018	0.27	0.018	1.03	0.012	1.00	0.014	4
31	0.92	0.010	0.25	0.017	0.26	0.017	1.03	0.012	1.01	0.015	4
32	0.92	0.010	0.25	0.017	0.26	0.017	1.02	0.016	1.01	0.014	4
33	0.92	0.010	0.24	0.017	0.25	0.017	1.02	0.016	1.02	0.016	4
34	0.91	0.011	0.24	0.017	0.25	0.017	1.01	0.019	1.01	0.021	4
35	0.91	0.010	0.23	0.016	0.24	0.017	1.01	0.018	1.02	0.020	4
36	0.91	0.010	0.22	0.016	0.24	0.016	1.00	0.017	1.02	0.020	4
37	0.91	0.011	0.22	0.016	0.23	0.016	1.00	0.018	1.02	0.020	4
38	0.90	0.011	0.21	0.015	0.23	0.016	1.00	0.022	1.03	0.022	4
39	0.90	0.011	0.21	0.015	0.22	0.016	0.99	0.021	1.02	0.022	4
40	0.90	0.011	0.21	0.015	0.22	0.016	1.00	0.020	1.02	0.021	4
41	0.90	0.011	0.20	0.015	0.21	0.016	1.00	0.020	1.02	0.025	4
42	0.89	0.012	0.20	0.014	0.21	0.015	1.00	0.025	1.02	0.027	4
43	0.89	0.012	0.19	0.014	0.20	0.015	0.99	0.028	1.03	0.027	4
44	0.89	0.013	0.19	0.014	0.20	0.015	0.99	0.025	1.03	0.026	4
45	0.89	0.012	0.19	0.014	0.20	0.015	0.99	0.024	1.03	0.027	4
46	0.89	0.012	0.18	0.013	0.19	0.015	0.99	0.025	1.04	0.027	4
47	0.88	0.012	0.18	0.013	0.19	0.015	0.99	0.026	1.03	0.024	4
48	0.88	0.013	0.17	0.013	0.19	0.014	0.99	0.026	1.04	0.023	4
49	0.88	0.013	0.17	0.013	0.18	0.014	0.99	0.026	1.04	0.027	4
50	0.88	0.013	0.17	0.013	0.18	0.014	1.00	0.025	1.04	0.025	4

Supplementary Section Table ST12. Photostability of green-emitting pH sensors in fixed or live cells, related to Figure 3. J774 macrophages were incubated overnight with 70 KDa amino-dextrans labeled with NHS-probe pH sensors, or 10 KDa dextrans labeled with LysoSensor™ yellow/blue, at 0.5 or 2 mg/mL, respectively, in complete DMEM medium, followed by a 4 h chase. LE/Lys were imaged in fixed cells (in pH 5.0 buffer containing methylamine, nigericin and monensin as membrane-permeant equilibrators) or live cells (in complete DMEM medium), irradiated with a 488 nm or a 405 nm laser for 50 cycles (0.5-0.6 seconds per pulse) in a confocal microscope incubation chamber equilibrated at 37 °C. Green fluorescence intensity was measured at time 0 (F_0) and after each irradiation cycle, separated by 1 to 8 second intervals (F). Fluorescence was normalized to time 0 (F_0) measurement. The experiment was repeated twice; 2 dishes or wells were measured per condition, and 3 to 4 fields were imaged per dish or well. Average F/F_0 fluorescence ± SEM is presented.

Supplementary Table ST13 related to Figure 4. Effect of dextran derivatization and charge on ApHID pKa
70 KDa Fina amino-dextrans. Descriptive statistics.

Buffer pH	ApHID:Alexa 405 1:2		ApHID:Alexa 405 1:1		ApHID:Alexa 405 3:2		ApHID:Alexa 405 2:1		n (exp)
	Av. ApHID/A405	± SEM	Av. ApHID/A405	± SEM	Av. ApHID/A405	± SEM	Av. ApHID/A405	± SEM	
**4**	1.00	0.082	3.10	0.032	4.89	0.071	8.10	0.104	3
**4.5**	0.83	0.054	2.53	0.007	4.09	0.081	6.62	0.124	3
**5**	0.55	0.047	1.71	0.035	2.73	0.048	4.31	0.086	3
**5.5**	0.28	0.031	0.88	0.026	1.32	0.026	2.04	0.041	3
**6**	0.06	0.014	0.25	0.012	0.36	0.013	0.56	0.008	3
**Buffer pH**	***normalized to pH 5.0***								
**4**	1.00	0.017	1.00	0.032	0.98	0.006	1.03	0.013	
**4.5**	0.84	0.030	0.82	0.031	0.82	0.014	0.84	0.016	
**5**	0.55	0.011	0.55	0.011	0.55	0.011	0.55	0.011	
**5.5**	0.28	0.006	0.28	0.012	0.27	0.008	0.26	0.005	
**6**	0.06	0.008	0.08	0.002	0.07	0.002	0.07	0.001	

Buffer pH	ApHID:Cy5 1.2:1.4		ApHID:Cy5 2.3:1.4		ApHID:Cy5 3.2:1.4		ApHID:Cy5 4.6:1.4		n (exp)
	Av. ApHID/Cy5	± SEM	Av. ApHID/Cy5	± SEM	Av. ApHID/Cy5	± SEM	Av. ApHID/Cy5	± SEM	
**4**	1.00	0.046	2.39	0.189	3.36	0.273	5.01	0.378	3
**4.5**	0.87	0.074	2.14	0.274	2.91	0.381	4.41	0.477	3
**5**	0.57	0.026	1.30	0.125	1.85	0.157	2.86	0.260	3
**5.5**	0.30	0.005	0.67	0.028	0.87	0.041	1.28	0.066	3
**6**	0.08	0.008	0.21	0.005	0.27	0.006	0.39	0.031	3
**Buffer pH**	***normalized to pH 5.0***								
**4**	1.00	0.097	1.05	0.074	1.03	0.092	0.99	0.075	
**4.5**	0.87	0.117	0.93	0.114	0.89	0.122	0.87	0.094	
**5**	0.57	0.051	0.57	0.051	0.57	0.051	0.57	0.051	
**5.5**	0.30	0.021	0.29	0.012	0.27	0.014	0.25	0.013	
**6**	0.08	0.004	0.09	0.002	0.08	0.002	0.08	0.006	

Buffer pH	ApHID		ApHID, 1.33x A405		ApHID, 6x A405		ApHID, 22x A405		n (exp)
	Av. ApHID/A405	± SEM	Av. ApHID/A405	± SEM	Av. ApHID/A405	± SEM	Av. ApHID/A405	± SEM	
**4**	1.00	0.000	1.00	0.000	1.00	0.000	1.00	0.000	2
**4.5**	0.86	0.000	0.80	0.012	0.86	0.014	0.91	0.012	2
**5**	0.58	0.026	0.56	0.001	0.61	0.020	0.79	0.013	2
**5.5**	0.27	0.018	0.26	0.004	0.29	0.006	0.47	0.008	2
**6**	0.11	0.008	0.08	0.001	0.10	0.002	0.27	0.048	2
**6.5**	0.03	0.000	0.03	0.002	0.04	0.006	0.14	0.089	2
**7**	0.01	0.002	0.01	0.002	0.01	0.001	0.06	0.010	2

Fluorescence ratio vs buffer pH - sigmoidal fit and pKa for each experiment

ApHID:A405	ApHID pKa	R^2	ApHID:Cy5•3SO3^{-}	ApHID pKa	R^2	Charge (ApHID:A405)	ApHID pKa	R^2
**1 : 2**	5.08	0.099	**1.2 : 1.4**	5.10	0.916	**Control no A405**	5.07	0.999
**1 : 1**	5.04	0.990	**2.3 : 1.4**	5.03	0.937	**1 : 1.33**	5.00	0.999
**3 : 2**	5.06	0.999	**3.2 : 1.4**	5.04	0.924	**1 : 6**	5.12	0.999
**2 : 1**	5.00	0.998	**4.6 : 1.4**	5.07	0.946	**1 : 22**	5.42	0.980

Supplementary Section Table ST13. Effect of dextran derivatization on ApHID pKa, related to Figure 4. The effect of various amounts of Alexa 405 and Cy5•3SO3^{-} dextran derivatization, as well as various dextran charge densities, on ApHID pKa was investigated. Charge density was introduced by labeling the polymers with various amounts of NHS-Alexa 405, which carries three negatively charged sulfate groups. 70 KDa amino-dextrans labeled with probes were diluted in buffers with pH adjusted between 4 and 7.0. The fluorescence of the probes was measured in solution using a spectrophotometer and ApHID/pH-independent fluorescence ratios were calculated and plotted against buffer pH, yielding titrations that were fitted to 4-component sigmoidal curves, from which log IC_{50} was calculated for each probe corresponding to their pKa. The amounts of Cy5•3SO3^{-} and Alexa 405 per molecule of dextran, relative to ApHID, are presented in the tables above, together with the resulting ApHID pKa (IC_{50}) calculated for that particular dextran. The adjusted R-squared of the sigmoidal fit (R^2) is shown for each calculated pKa. The experiment was repeated two or three times.

Supplementary Table ST14 related to Figure 5B-5D.
Cytotoxicity of ApHID toward J774 macrophages measured by cell count (Hoechst staining). 70 KDa amino-dextrans. Descriptive statistics.

Well #	Control no dextran 24h	ApHID-dextran 24h
	Norm. cell count (Hoechst staining)	
1	0.90	0.90
2	0.99	1.15
3	0.98	1.13
4	1.13	0.93
5	0.97	0.94
6	0.99	0.96
7	1.04	0.96
8	0.96	0.91
9	1.05	1.11
10	0.98	1.07
**Average ± SEM**	1.00 ± 0.02	1.006 ± 0.03

Two-tailed unpaired Student's t test

Comparison	*Mean 1*	*Mean 2*
CRTL no dex 24h	1.00	
ApHID-dex 24h		1.006
Mean Diff.	***p value***	***Significance***
0.006	0.88	n.s

Supplementary Table ST14. Cytotoxicity of ApHID toward J774 macrophages, related to Figure 5B-5D. J774 macrophages were incubated with 0.5 mg/mL amino-dextrans (70 KDa) labeled with NHS-ApHID, or left untreated in complete DMEM medium for an overnight period, followed by a 4 h chase in fresh complete DMEM. Cell nuclei were labeled with Hoechst and imaged live in a confocal microscope incubation chamber equilibrated at 37 °C in 5% CO_2. The experiment was repeated three times; 10 wells were imaged in total, and 16 fields were acquired per well. The total number of cells per well was calculated by summing the nuclei counts from all imaged fields in each well and normalized to the untreated condition. Normalized cell count is shown for each well together with the resulting averaged cell count ± SEM. Statistical differences between the control no dextran condition and the dextran-treated condition were assessed using the two-tailed, unpaired Student's t-test ($p>0.05$).

Supplementary Table ST15 related to Figure 5E-5I.
Effect of overnight exposure to acidic LE/Ly environment on ApHID fluorescence and pKa.
70 KDa amino-dextrans. Descriptive statistics.

Buffer pH	J774 (confocal imaging)			Spectrophotometer (in solution)		
	Av. ApHID/Alexa 647	± SEM	n	Av. ApHID/Alexa 647	± SEM	n
4.05	1.00		3	1.00		2
4.53	0.77	0.02	3	0.79	0.00	2
5.02	0.45	0.00	3	0.48	0.01	2
5.53	0.22	0.00	3	0.19	0.01	2
6	0.08	0.00	3	0.08	0.00	2
**log IC_{50} (pKa)**	4.77			4.89		
**R^2**	0.998			0.999		

Supplementary Table ST15. Effect of overnight exposure to acidic LE/Ly environment on ApHID fluorescence and pKa, related to Figure 5E-5I. Dextrans labeled with ApHID and Alexa 647 were loaded into J774 macrophage LE/Ly compartments during an overnight incubation, followed by a 4 h chase in fresh complete medium. Cells were thereafter fixed in 0.5% PFA, followed by the addition of buffers with pH adjusted between 4.0 and 6 containing membrane-permeant equilibrators. The cells were equilibrated in buffer for 20-30 min at 37 °C inside a confocal microscope incubation chamber, followed by confocal imaging. The experiment was repeated three times, 2 wells were imaged for each buffer pH condition, and 3 fields were acquired per well. The same dextran used to incubate J774 macrophages was also measured in solution at 37 °C, using a spectrophotometer (see Suppl. Fig. S3A). Average ApHID/Alexa 647 ± SEM for each buffer pH is shown. Fluorescence ratios measured for fixed J774 macrophages or in solution were plotted against buffer pH, yielding titrations that were fitted to a 4-component sigmoidal curve from which log IC_{50} was calculated for each probe corresponding to their pKa. The adjusted R-squared of the sigmoidal fit (R^2) is shown for each calculated pKa.

Supplementary Table ST16 related to Figure 5J-5Q. LE/Ly pH and ratios measured using 70 KDa ApHID-Alexa 647 amino-dextrans, per cell and experiment. Descriptive statistics.

	Interpolated pH						ApHID/Alexa 647 ratio					
	Per cell											
	untreated			20 mM $MeNH_2$			untreated			20 mM $MeNH_2$		
	Exp 1	*Exp2*	*Exp 3*	*Exp 1*	*Exp2*	*Exp 3*	*Exp 1*	*Exp2*	*Exp 3*	*Exp 1*	*Exp2*	*Exp 3*
**n (cells)**	208	275	120	195	336	152	208	275	120	195	336	152
**Av. LE/Ly pH or ratio**	5.02	5.11	5.07	6.44	6.25	6.24	0.513	0.501	0.511	0.0282	0.0495	0.048
**SD (cells)**	0.0614	0.0633	0.0643	0.0398	0.0628	0.0771	0.0476	0.0501	0.0522	0.00259	0.00698	0.00794
**SEM (cells)**	0.00426	0.00382	0.00587	0.00285	0.00343	0.00625	0.0033	0.00302	0.00476	0.00019	0.00038	0.00064
**Total n (cells)**	603			683			603			683		
**Av. pH or ratio ± SEM**	5.07 ± 0.003			6.30 ± 0.004			0.51 ± 0.002			0.04 ± 0.0004		
	Per LE/Ly compartment											
	untreated			20 mM $MeNH_2$			untreated			20 mM $MeNH_2$		
	Exp 1	*Exp2*	*Exp 3*	*Exp 1*	*Exp2*	*Exp 3*	*Exp 1*	*Exp2*	*Exp 3*	*Exp 1*	*Exp2*	*Exp 3*
**n (LE/Lys)**	46872	104313	35825	38505	104407	41925	46872	104313	35825	38505	104407	41925
**Av. LE/Ly pH or ratio**	4.99	5.13	5.08	6.44	6.28	6.31	0.47	0.45	0.45	0.03	0.04	0.04
**SD (LE/Lys)**	0.202	0.214	0.226	0.207	0.218	0.340	0.135	0.146	0.153	0.009	0.017	0.020
**Total n (LE/Lys)**	187010			184837			187010			184837		
**Av. pH or ratio ± SD**	5.08 ± 0.22			6.32 ± 0.26			0.46 ± 0.15			0.04 ± 0.02		
**LE/Lys imaged/cell**	310			271			310			271		

Supplementary Table ST16. High-resolution LE/Ly pH imaging in J774 macrophages using ApHID, related to Figure 5J-5Q. Cells were incubated with ApHID-Alexa 647 amino-dextrans (70 KDa) overnight and chased in fresh complete DMEM medium the following morning. LE/Lys were imaged by confocal microscopy using a 63X air objective. Some cells were treated with 20 mM methylamine to alkalinize compartments. ApHID/Alexa 647 ratios were calculated per cell or per LE/Ly compartment and interpolated to pH using a ratio-to-pH calibration prepared in fixed cells. To generate the calibration, cells loaded with dextrans were fixed in 0.5% PFA and imaged in 50 mM TRIS maleate pH 5.0 buffer containing membrane-permeant equilibrators. The fluorescence ratios corresponding to pH 5.0 were used to generate a full calibration using titration data previously obtained in solution (see Figures 5E-5I and Suppl. Fig. S3A). The experiment was repeated three times, 3 wells were imaged per condition and 4 fields were acquired per well, for a total of 9 wells and 36 fields. Overall, 603 and 683 cells (187,010 and 184,837 LE/Ly compartments) were quantified for the untreated and methylamine-treated conditions, respectively. Average LE/Ly pH or ApHID/Alexa 647 ratio ± SEM or ± SD are presented. Abbreviations: $MeNH_2$: methylamine; Exp: Experiment.

Supplementary Table ST17 related to Figure 6J. Continuous LE/Ly pH imaging of J774 macrophages. Descriptive statistics.

	ApHID			Fluorescein			Oregon Green		
**Time (min)**	Av. LE/Ly pH	± SEM	n (wells)	Av. LE/Ly pH	± SEM	n (wells)	Av. LE/Ly pH	± SEM	n (well)
**20**	5.03	0.07	6	5.05	0.06	6	4.93	0.11	6
**25**	5.02	0.10	6	5.07	0.04	6	4.98	0.06	6
**30**	5.01	0.11	6	5.07	0.03	6	4.99	0.03	6
**35**	4.99	0.10	6	5.07	0.03	6	4.99	0.02	6
**40**	4.99	0.09	6	5.07	0.03	6	4.99	0.02	6
**45**	4.99	0.09	6	5.07	0.03	6	4.99	0.02	6
**50**	4.99	0.08	6	5.07	0.03	6	4.98	0.02	6
**55**	4.99	0.08	6	5.06	0.03	6	4.98	0.02	6
**60**	4.99	0.08	6	5.06	0.03	6	4.98	0.02	6
**65**	4.99	0.08	6	5.06	0.02	6	4.98	0.02	6
**70**	4.99	0.08	6	5.06	0.02	6	4.98	0.02	6
**75**	4.99	0.08	6	5.06	0.03	6	4.97	0.02	6

Supplementary Section Table ST17. Continuous LE/Ly pH imaging of J774 macrophages over the course of a 75 min acquisition, related to Figure 6J. Cells were incubated with 70 KDa amino-dextrans labeled with NHS-ApHID, NHS-fluorescein or NHS-Oregon Green and NHS-Alexa 647 (pH-independent). Images were acquired every 5 min. Fluorescence ratios were calculated and interpolated to pH using a ratio-to-pH calibration prepared in fixed cells as described above. The experiment was repeated three times, 2 wells were imaged per experiment, and 4 fields were acquired per well. Average pH ± SEM per well is shown.

Supplementary Table ST18 related to Figure 6K. Measuring LE/Ly pH in J774 macrophages using ApHID, fluorescein and Oregon Green (1 h equilibration at 37 °C). 70 KDa amino-dextrans. Descriptive statistics.						
	**ApHID**	**ApHID + $MeNH_2$**	**Fluorescein**	**Fcein + $MeNH_2$**	**Oregon Green**	**OG + $MeNH_2$**
**Average pH ± SEM (per well)**	4.99 ± 0.03	6.42 ± 0.01	5.06 ± 0.01	6.28 ± 0.01	4.98 ± 0.01	6.56 ± 0.13
**Average probe/A647 ratio ± SEM**	0.78 ± 0.07	0.05 ± 0.01	0.22 ± 0.03	1.3 ± 0.14	1.35 ± 0.14	-
**n (wells)**	6	6	6	6	6	-

One-way Welch and Brown-Forsythe ANOVA (Dunnett's multiple comparison test) - n: wells					
Comparison	***Mean 1***	***Mean 2***	***Mean Diff.***	***p value***	***Significance***
ApHID vs. Fluorescein	4.99	5.06	-0.07	0.3	n.s
ApHID vs. OG	4.99	4.98	0.02	0.97	n.s.
ApHID vs +MeNH2	4.99	6.42	-1.43	<0.0001	****
Fluorescein vs. +MeNH2	5.06	6.28	-1.21	<0.0001	****
OG vs. +MeNH2	4.98	6.56	-1.6	<0.001	***

Supplementary Table ST18. LE/Ly pH reported for J774 macrophages using ApHID, fluorescein or Oregon Green, related to Figure 6K. Cells were incubated with 70 KDa amino-dextrans tagged with NHS-ApHID, NHS-fluorescein or NHS-Oregon Green and NHS-Alexa 647 (pH-independent) overnight and chased for 4 h in fresh DMEM medium the next morning. Probe/Alexa 647 ratios were calculated and interpolated to pH using a ratio-to-pH calibration as described above. The experiment was repeated three times, 2 wells were imaged for each condition and experiment, and four fields were acquired per well. Average LE/Ly pH ± SEM per well is shown. Differences in LE/Ly pH means between conditions were assessed using the unpaired Welch and Brown-Forsythe one-way ANOVA followed by Dunnett’s multiple comparison test with 95% confidence interval. P-values shown as p>0.05 (ns), p≤0.001 (***), and p≤0.0001 (****). Abbreviations: ‘OG’: Oregon Green; ‘Fcein’: fluorescein; $MeNH_2$: methylamine.

Supplementary Table ST19 related to Figure 7A-7C. Continuous LE/Ly pH imaging of murine bone marrow-derived macrophages using ApHID. 70 KDa amino-dextrans. Descriptive statistics.					
**Time (hours)**	**av. LE/Ly pH**	**± SEM**	**ApHID/A647 ratio**	**± SEM**	**n (wells)**
**2**	5.13	0.10	0.84	0.02	6
**3**	5.12	0.09	0.86	0.01	6
**4**	5.13	0.08	0.83	0.02	6
**5**	5.14	0.08	0.83	0.02	6
**6**	5.16	0.08	0.81	0.02	6
**7**	5.15	0.07	0.82	0.03	6
**8**	5.15	0.07	0.82	0.03	6
**9**	5.16	0.07	0.81	0.03	6
**10**	5.16	0.07	0.81	0.03	6
**11**	5.16	0.07	0.81	0.03	6
**12**	5.17	0.07	0.80	0.02	6
**13**	5.15	0.07	0.82	0.03	6
**14**	5.17	0.07	0.79	0.03	6
**15**	5.17	0.06	0.79	0.03	6

Supplementary Table ST19. Continuous 15h imaging of LE/Ly pH in live primary bone marrow-derived macrophages using ApHID, related to Figure 7A-7C. Murine bone marrow-derived macrophages (BMMs) were incubated with 70 KDa amino-dextrans labeled with NHS-ApHID and NHS-Alexa 647 overnight and chased for 4 h in fresh DMEM the following morning. Cells were then equilibrated inside a confocal microscope incubation chamber at 37 °C in 5% CO_2 for 2 h prior to imaging. ApHID/Alexa 647 ratios were calculated and interpolated to pH using a ratio-to-pH calibration as described above. The experiment was repeated twice, 3 wells were imaged per experiment, and 6 fields were acquired per well. Fields were acquired every hour. Average LE/Ly ± SEM per well is shown.

Supplementary Table ST20 related to Figure 7D-7J. LE/Ly pH imaging of J774 macrophages using ApHID or LysoSensor™ yellow/blue. 70 KDa or 10 KDa amino-dextrans. Descriptive statistics and comparisons.

	ApHID untreated	ApH +4 mM $MeNH_2$	ApH +8 mM $MeNH_2$	LSyb untreated	LSyb +4 mM $MeNH_2$	LSyb +8 mM $MeNH_2$
**n (wells)**	12	6	6	10	6	3
**Average LE/Ly pH ± SEM**	5.14 ± 0.01	5.50 ± 0.02	5.66 ± 0.02	5.67 ± 0.08	6.61 ± 0.23	7.18 ± 0.36
**Average pH-s/pH-I ratio ± SEM**	0.91 ± 0.06	0.48 ± 0.05	0.35 ± 0.04	0.74 ± 0.02	0.64 ± 0.02	0.54 ± 0.03

One-way Welch and Brown-Forsythe ANOVA (Dunnett's multiple comparison test) - n: wells (LE/Ly pH)

Comparison	***Mean 1***	***Mean 2***	***Mean Diff.***	***p value***	***Significance***
ApHID untreated vs 4 mM $MeNH_2$	5.14	5.5	-0.36	<0.0001	****
ApHID untreated vs 8 mM $MeNH_2$	5.14	5.66	-0.52	<0.0001	****
ApHID vs LSyb untreated	5.14	5.67	-0.53	<0.001	***
ApHID vs LSyb 4 mM $MeNH_2$	5.14	6.61	-1.11	0.03	*
ApHID vs LSyb 8 mM $MeNH_2$	5.14	7.18	-1.51	0.16	ns
LSyb vs 4 mM $MeNH_2$	5.67	6.61	0.94	0.045	*
LSyb vs 8 mM $MeNH_2$	5.67	7.18	-1.51	0.17	ns

Supplementary Table ST20. LE/Ly pH imaging of J774 macrophages using ApHID and LysoSensor™ yellow/blue in parallel, related to Figure 7D-7J. Cells were incubated with ApHID-Alexa 647 amino-dextrans (70 KDa) or LysoSensor™ yellow/blue (10 KDa dextrans) overnight followed by a 2-6 h chase in fresh DMEM medium. The plates containing the cells were equilibrated inside a confocal microscope incubation chamber for at least 20 min at 37 °C and 5% CO_2 prior to imaging. Some cells were briefly incubated with 4 mM or 8 mM methylamine to induce subtle compartment alkalinization. LSyb was imaged ratiometrically. The probe was excited using a 405 nm solid-state laser. Its pH-sensitive emission mode (LSyb pH-s) was recorded by acquiring fluorescence between 500-600 nm, whereas its pH-independent mode (LSyb pH-i) was recorded by acquiring fluorescence between 410-494 nm. ApHID/Alexa 647 and LSyb pH-s/pH-I fluorescence ratios were calculated and interpolated to pH using ratio-to-pH calibrations as described above. The experiment was repeated three times, 2 to 4 wells were imaged per condition, and 6 fields were acquired per well. Average LE/Ly pH ± SEM per well is shown. Differences in pH means between probes and conditions were assessed using the unpaired one-way Welch and Brown-Forsythe ANOVA followed by Dunnett's multiple comparison test with 95% confidence interval. P-values shown as $p>0.05$ (ns), $p\leq0.05$ (*), $p\leq0.001$ (***), and $p\leq0.0001$ (****). Abbreviations: ApH: ApHID; LSyb: LysoSensor™ yellow/blue; pH-s/pH-I: pH-sensitive/pH-independent; $MeNH_2$: methylamine.

Methods S1. ApHID synthetic protocol, related to STAR Methods.

Synthesis of known precursors

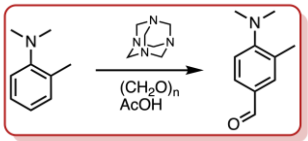

Reference: Gawinecki et *al.,* 1998 [S2].

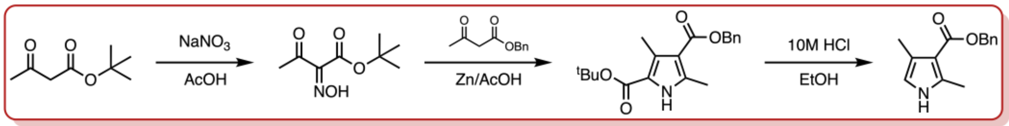

Reference: Li et *al*., 2015 [S3].

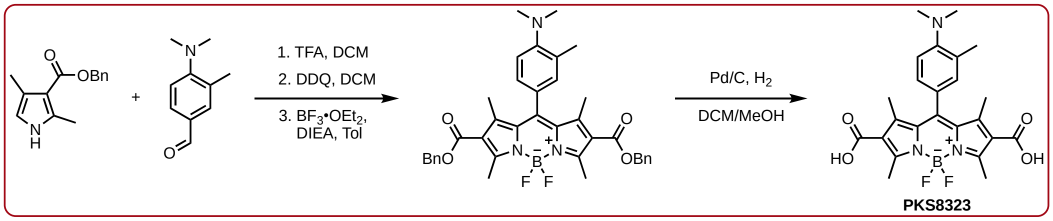

Reference: Maeda et *al*., 2016 [S4].

Synthesis of new compounds

Synthesis of PKS8324

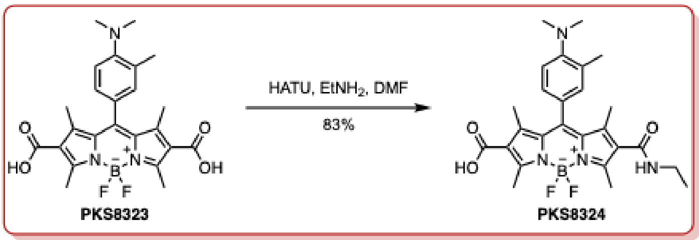

Synthesis of PKS8325

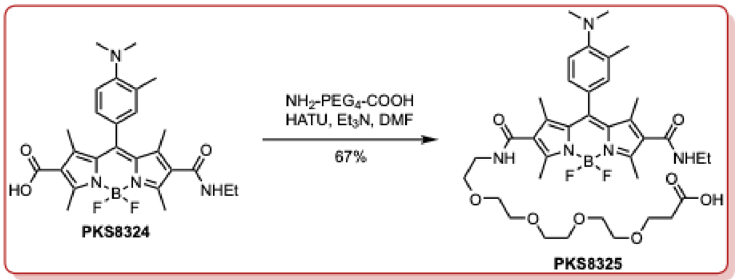

Synthesis of PKS8326

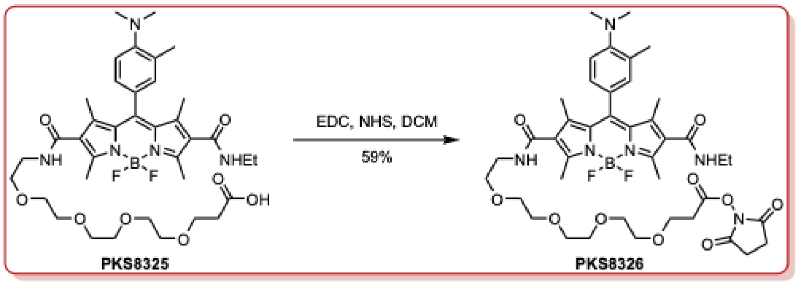

Data S1. NMR Spectroscopy of ApHID and its precursors, related to STAR Methods.

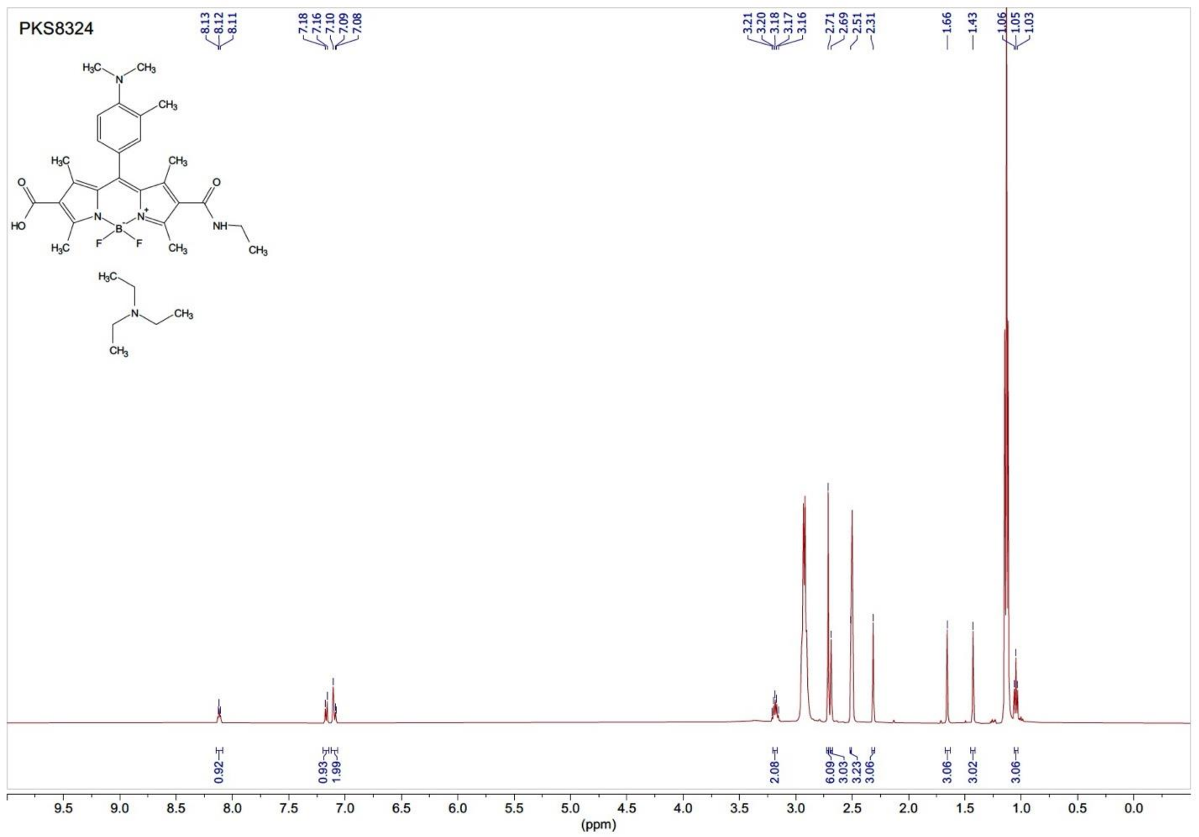

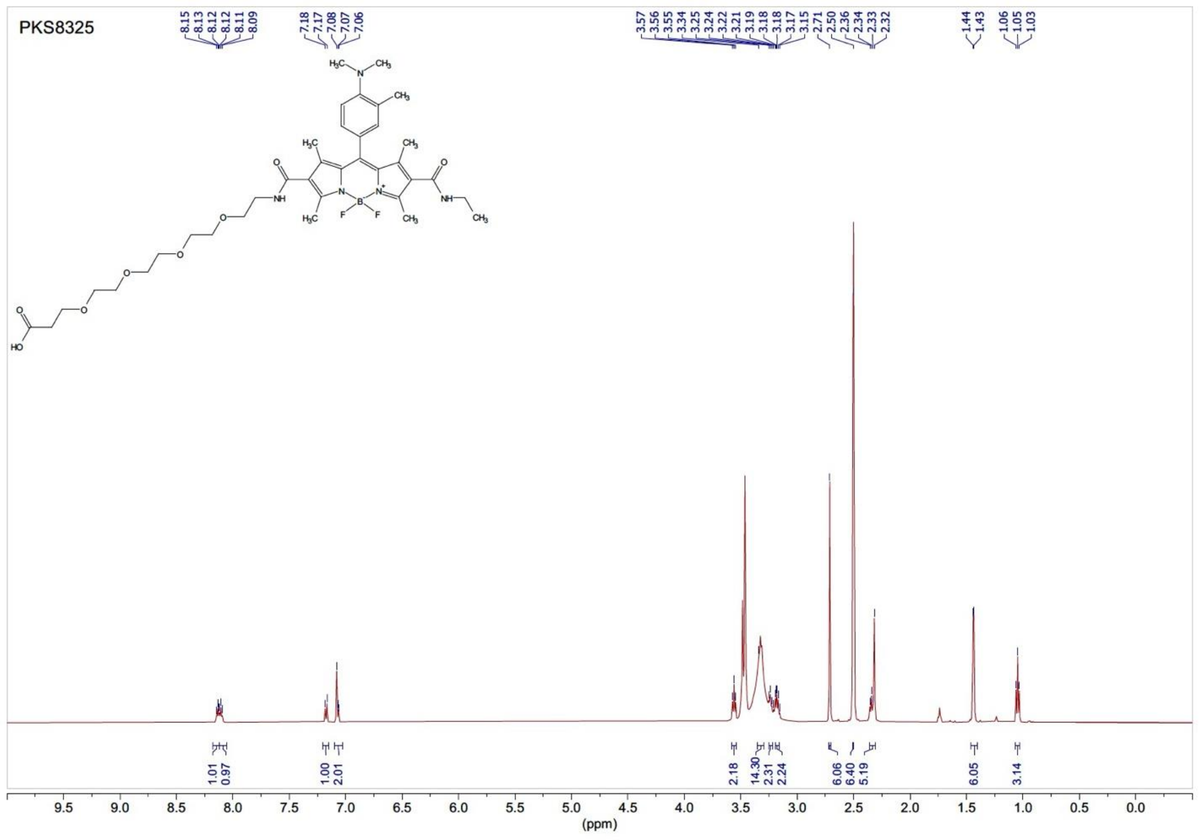

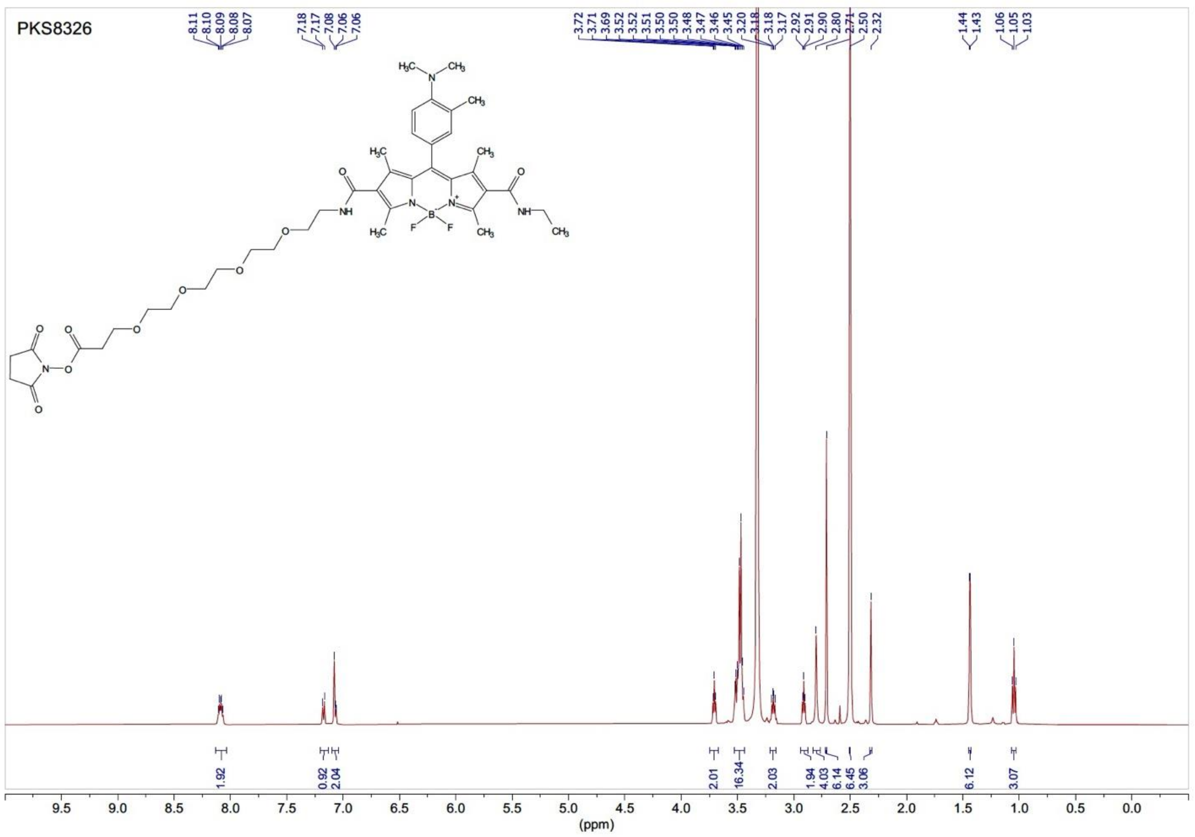
PKS8326
(ppm)

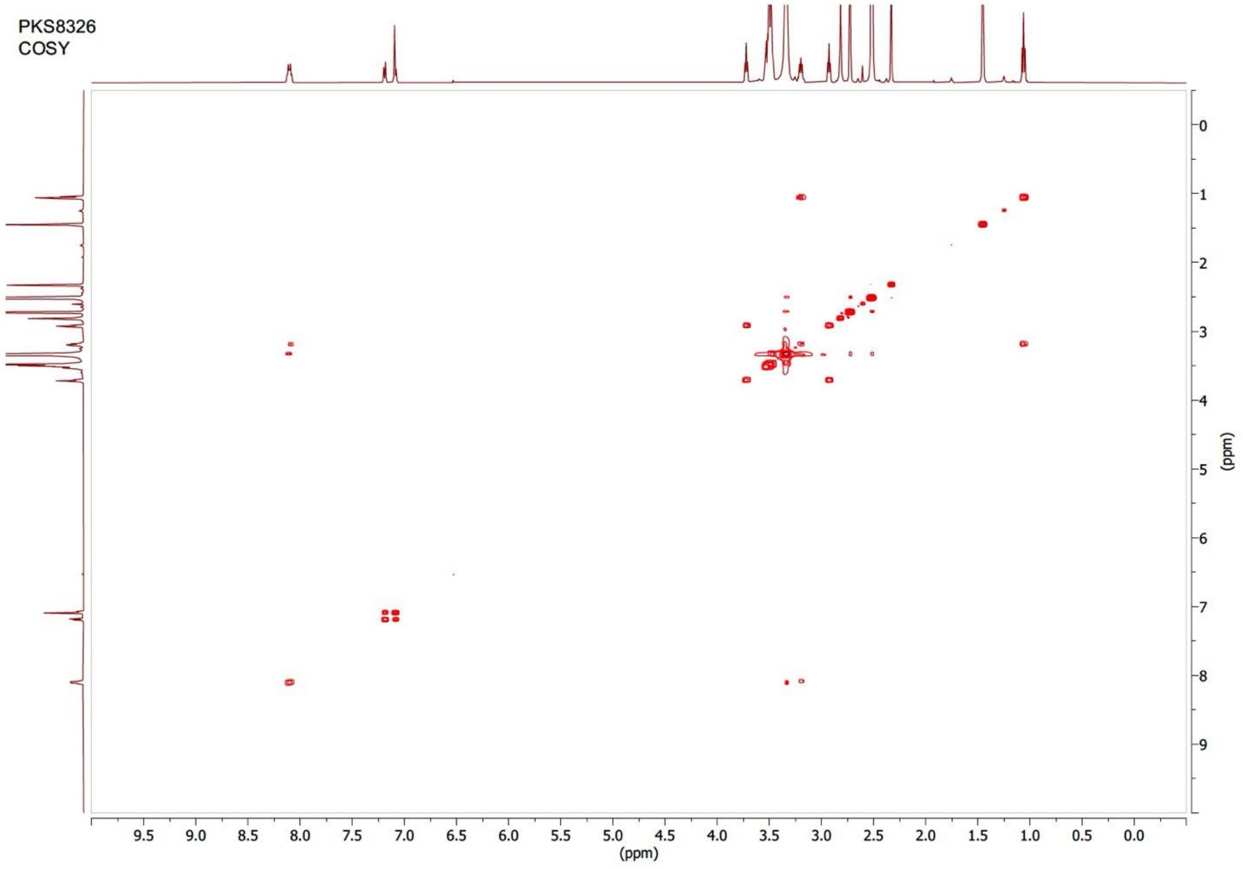
PKS8326
COSY
(ppm)

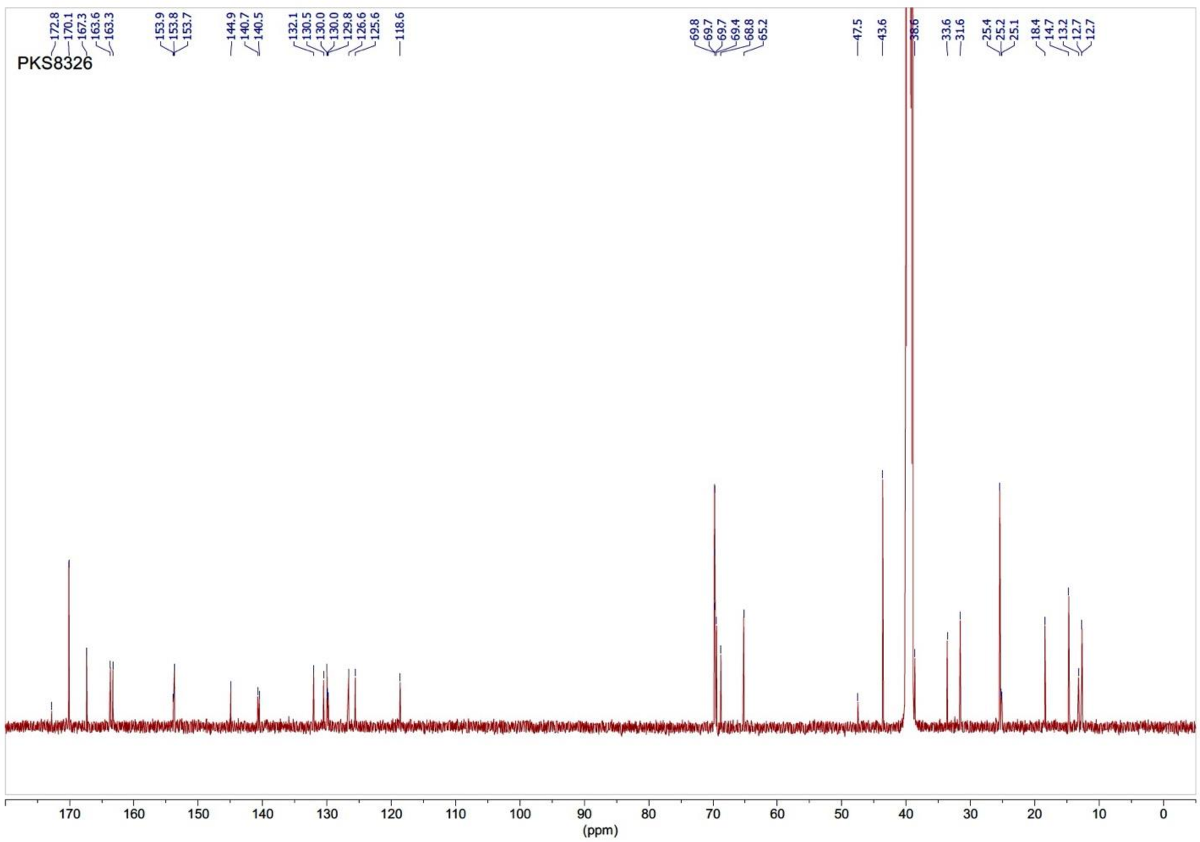
PKS8326
172.8
170.1
167.3
163.6
163.3
153.9
153.8
153.7
144.9
140.7
140.5
132.1
130.5
130.0
130.0
129.8
126.6
125.6
118.6
69.8
69.7
69.7
69.4
68.8
65.2
47.5
43.6
38.6
33.6
31.6
25.4
25.2
25.1
18.4
14.7
13.2
12.7
12.7
(ppm)

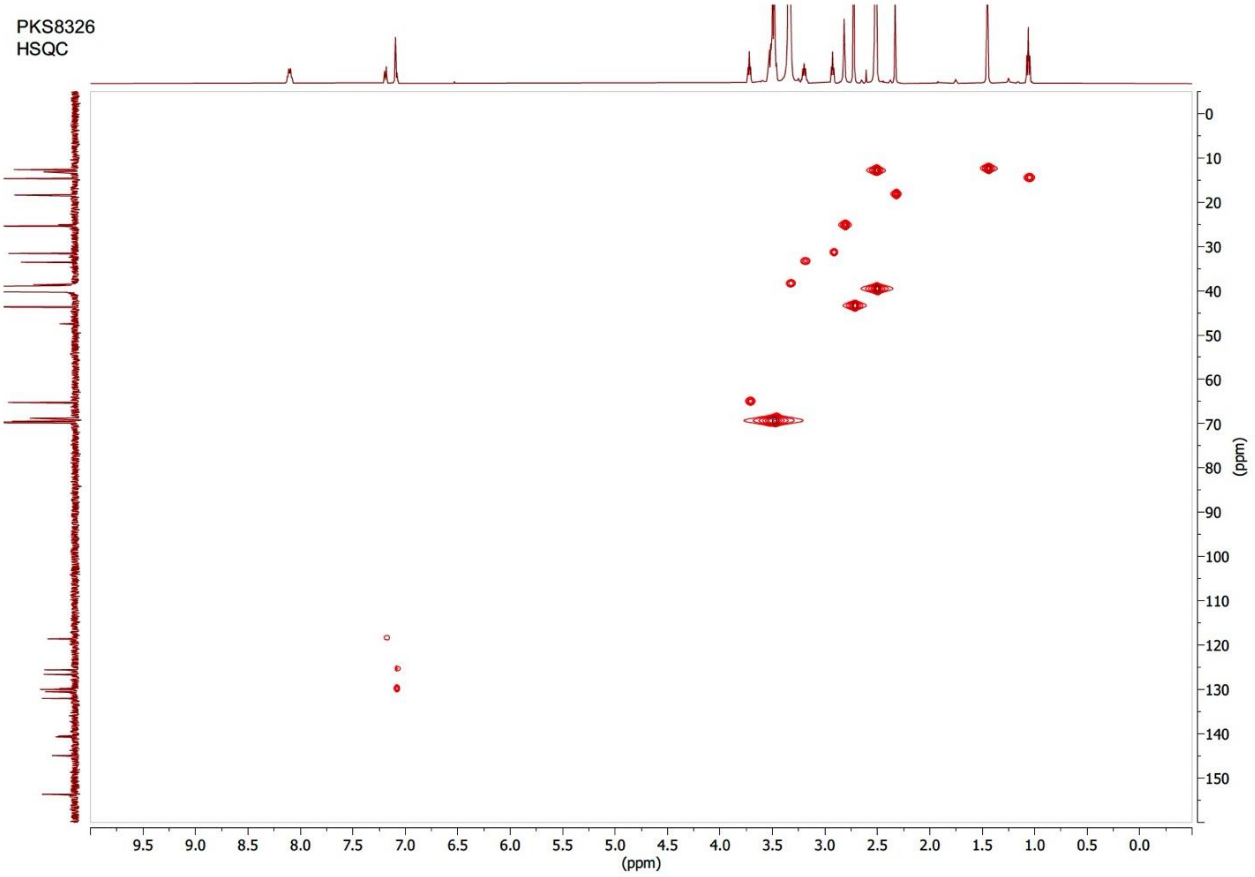
PKS8326
HSQC
(ppm)
(ppm)

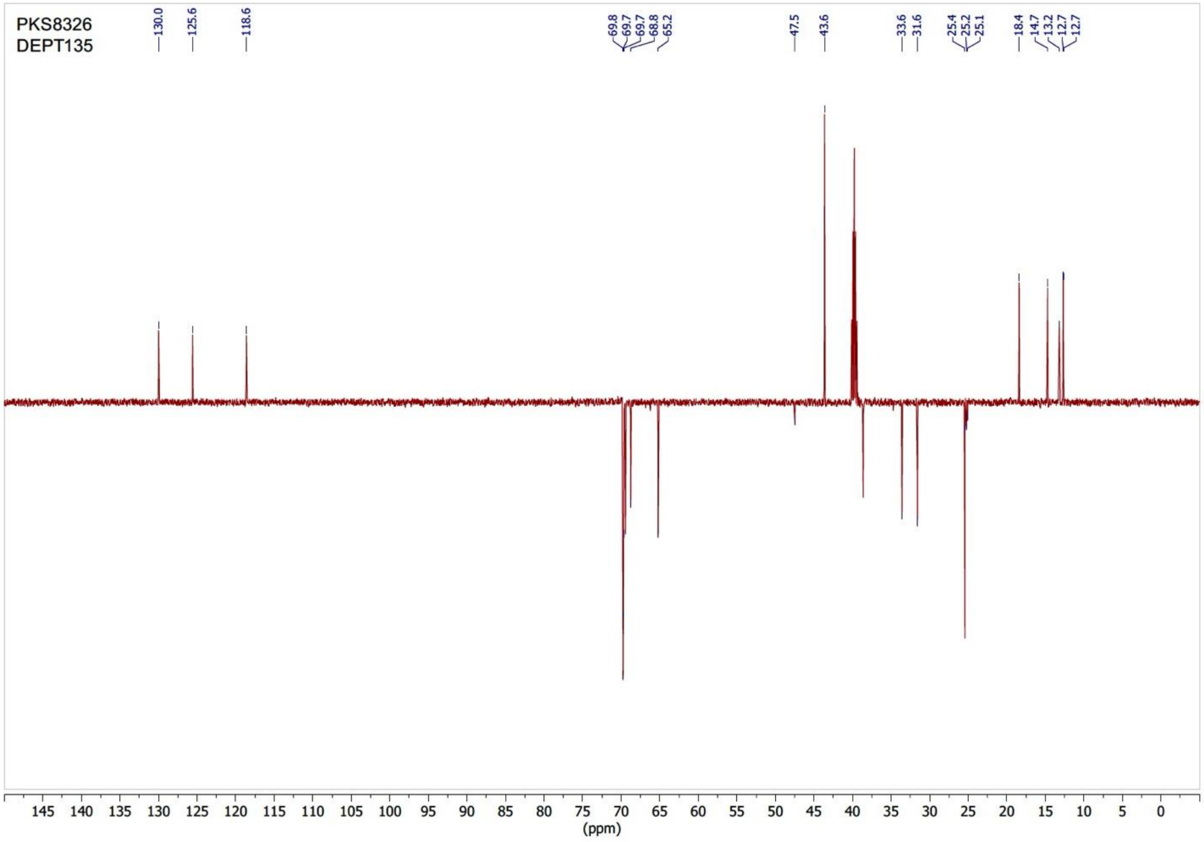
PKS8326
DEPT135
130.0
125.6
118.6
69.8
69.7
69.7
68.8
65.2
47.5
43.6
33.6
31.6
25.4
25.2
25.1
18.4
14.7
13.2
12.7
12.7
145 140 135 130 125 120 115 110 105 100 95 90 85 80 75 70 65 60 55 50 45 40 35 30 25 20 15 10 5 0
(ppm)

SUPPLEMENTARY REFERENCES

[S1] Hoffmann, B., and Kosegarten, H. (1995). FITC-dextran for measuring apoplast pH and apoplastic pH gradients between various cell types in sunflower leaves. Physiol Plantarum *95*, 327–335. https://doi.org/10.1111/j.1399-3054.1995.tb00846.x.

[S2] Gawinecki, R., Andrzejak, S., and Puchala, A. (1998). Efficiency of the Vilsmeier-Haack method in the synthesis of p-aminobenzaldehydes. Organic Preparations and Procedures International *30*, 455–460. Doi 10.1080/00304949809355310.

[S3] Li, M., Yao, Y., Ding, J., Liu, L., Qin, J., Zhao, Y., Hou, H., and Fan, Y. (2015). Spectroscopic and crystallographic investigations of novel BODIPY-derived metal-organic frameworks. Inorg Chem *54*, 1346–1353. 10.1021/ic502219y.

[S4] Maeda, H., Kowada, T., Kikuta, J., Furuya, M., Shirazaki, M., Mizukami, S., Ishii, M., and Kikuchi, K. (2016). Real-time intravital imaging of pH variation associated with osteoclast activity. Nat Chem Biol *12*, 579–585. 10.1038/nchembio.2096.
